# Proteomic associations with fluctuation and long‐term changes in BMI: A 40‐year follow‐up study

**DOI:** 10.1111/dom.16448

**Published:** 2025-05-08

**Authors:** Alvaro Obeso, Gabin Drouard, Teemu Palviainen, Xiaoling Wang, Miina Ollikainen, Karri Silventoinen, Jaakko Kaprio

**Affiliations:** ^1^ Department of Genetics, Physical Anthropology and Animal Physiology Faculty of Science and Technology, University of the Basque Country Bilbao Spain; ^2^ Helsinki Institute for Demography and Population Health University of Helsinki Helsinki Finland; ^3^ Institute for Molecular Medicine Finland, HiLIFE University of Helsinki Helsinki Finland; ^4^ Medical College of Georgia Augusta University Augusta Georgia USA; ^5^ Minerva Foundation Institute for Medical Research Helsinki Finland

**Keywords:** cohort study, database research, obesity care, weight control

## Abstract

**Introduction:**

While some studies have explored associations between weight change and blood proteins, most have been intervention‐based, offering limited insight into proteomic associations with long‐term weight gain. It remains unclear whether plasma proteins are related to BMI fluctuation over time. This study investigates associations of long‐term BMI changes and fluctuations with over 1000 plasma proteins involved in cardiometabolic and inflammation functions.

**Data and Methods:**

The study included 304 Finnish adult twins (117 men) born before 1958 from the Older Finnish Twin Cohort, with BMI data spanning five time points (1975, 1981, 1990, 2011 and 2012–2014). Proteomic data were derived from blood samples collected at the last BMI measurement. Linear mixed‐effects models analysed individual BMI trajectories, producing intercepts (baseline BMI) and slopes (BMI change rates). BMI fluctuation was calculated as the average squared deviation from expected BMI across time points. Associations between BMI changes/fluctuation and (i) 1231 plasma proteins related to cardiometabolic and inflammatory functions and (ii) polygenic risk scores for BMI (PRS_BMI_), as well as interaction effects between PRS_BMI_ and baseline BMI on protein‐BMI relationships were studied. Within‐pair analyses using monozygotic twins were conducted to account for shared confounding factors.

**Results:**

A total of 135 proteins were associated with changes in BMI over 40 years, while 17 proteins were linked to fluctuation in BMI: 12 associations (10 with BMI changes and 2 with fluctuation) remained significant in within‐twin pair analyses. PRS_BMI_ was associated with BMI changes but not with fluctuation. PRSBMI‐protein interactions explaining BMI changes or fluctuation were found, though a single interaction between the antigen CD72 protein and baseline BMI was observed.

**Conclusion:**

This study highlights significant associations between plasma proteins and long‐term BMI changes and fluctuations, with no evidence of PRS_BMI_ ‐protein interactions influencing BMI trends. These findings underscore the substantial role of environmental factors in shaping proteome‐BMI associations over adulthood.

## 1 INTRODUCTION

Obesity is considered a major global public health challenge, with the proportion of adults with obesity more than doubling from 7% to 16% over the past three decades.^1^ While obesity is primarily caused by energy consumption exceeding energy expenditure, the underlying factors are multifaceted and influenced by environmental, behavioural and genetic factors, as well as their interactions.^2^ Body mass index (BMI) has been widely used to classify overweight and obesity in epidemiological studies. However, obesity is a multifaceted concept, and the definition depends on whether it is used for epidemiological or clinical settings. In clinical settings, the definition of obesity needs to include other anthropometric and metabolic measures, emphasising the need to understand how BMI is associated with other biological traits.^3^ Nevertheless, measuring BMI remains a key part of the diagnostic process, and results based on using BMI are likely applicable to obesity research. This is supported by the strong correlations of BMI with other obesity indicators, such as fat mass and body fat percentage,^3^ as well as reported associations of BMI with obesity development.^4^ BMI changes and fluctuation are two variables that have shown special importance in the study of obesity development. Studying weight change, particularly weight gain, may help identify individuals at risk of obesity in the near‐to‐long term.^5^ Understanding how and why some individuals gain more weight than others could inform early prevention strategies, especially since obesity often persists, highlighting the importance of identifying at‐risk individuals. Moreover, BMI fluctuation has significant public health and clinical implications, as frequent weight cycling may increase the risk of future weight gain.^6^, ^7^

The proteome refers to a set of proteins produced by an organism or present within an organism or tissue at a given time. It can be measured in various tissues, but it is most commonly examined in blood in human studies. The proteome is dynamic and intricate, undergoing changes over time in response to environmental factors that affect genome function, with genetic makeup also playing a significant role.^8^ Recent developments in proteomics research enable the examination of the connections between changes in BMI and protein levels.

The associations between plasma proteins and obesity have been extensively studied, with a number of proteins involved in cardiometabolic^9^ and inflammatory processes identified.^10^, ^11^, ^12^ However, longitudinal proteomic studies are still rare, with the majority being interventions.^13^, ^14^, ^15^, ^16^ These studies have compared blood protein levels before and after weight loss, reporting various associations. Previous studies have demonstrated that certain blood proteins initially fluctuated in response to the acute energy‐deficient state, returning to their original levels once weight maintenance was achieved.^13^ Conversely, another group of proteins showed a decline in concentration during weight loss, with levels remaining stable during weight maintenance.^13^ The associations between cardiovascular and inflammatory biomarkers and BMI levels before and during the weight loss process have also been found to undergo a transformation, with the emergence of new markers associated with this period of weight loss.^17^ This suggests that blood proteins may exhibit disparate responses to short‐term weight changes, which can be caused by dietary alterations. Some of these changes may reflect the weight loss process, while others may also reflect weight status. Additionally, weight loss typically involves a loss of both fat and lean body mass, while weight gain is primarily due to an accumulation of fat mass.^18^, ^19^, ^20^ Therefore, the physiological and metabolic processes underlying weight gain and weight loss likely differ.

However, it is important to consider the time span of short‐term weight loss vs. weight gain over years or decades. Further investigation is needed to determine if associations between plasma proteins and long‐term changes in BMI exist, and if these proteins are influenced by baseline weight. Finally, long‐term studies of weight change have shown that individuals may lose weight in the short term but gain weight in the long term.^21^, ^22^ These fluctuations in BMI (i.e., BMI fluctuation over time) may be due to various reasons, including dieting and subsequent rebound. BMI fluctuation may also indicate a disease state, with erratic weight changes potentially indicating poor health status. For example, studies have shown negative effects of fluctuation in blood pressure and weight on coronary heart disease incidence and mortality, and all‐cause mortality.^23^, ^24^ To our knowledge, no study has examined how long‐term fluctuation in BMI may be reflected at the proteome level.

BMI and its changes are influenced by both genetic and environmental factors. Heritability estimates have ranged between 0.47–0.90 in twin studies and between 0.24–0.81 in family studies.^25^, ^26^, ^27^, ^28^ Additionally, genome‐wide association studies (GWAS) have identified multiple genetic variants associated with BMI during adulthood.^29^ These variants can be aggregated as polygenic risk scores (PRS), which can be used to estimate an individual's genetic predisposition to traits such as BMI.^30^ Furthermore, some of these genetic associations are pleiotropic, meaning that they are associated with multiple traits. Connections between proteins and genetic variants associated with BMI have also been identified.^31^, ^32^ In summary, while there is evidence of connections between genetic variants, BMI and the proteome, the extent to which proteins are associated with BMI changes over time remains underexplored.

To gain a deeper understanding of the associations between BMI changes and the proteome, we studied a sample of Finnish twins over a 40‐year follow‐up for whom plasma proteomic data was available. Additionally, we conducted within‐pair analyses on monozygotic (MZ) twin pairs to investigate whether the identified associations persisted when controlling for shared environmental and genetic factors between the co‐twins. Finally, an association analysis including interaction effects was carried out to study how genetic predisposition to BMI (PRS_BMI_) modulates the associations between BMI change and the proteome. This analysis reveals whether these associations are accentuated in individuals with a higher PRS_BMI_.

## 2 DATA AND METHODS

### 2.1 Cohort

The data was obtained from the Older Finnish Twin Cohort (FTC), a population‐based study consisting of twins born before 1958 in Finland. The twins completed up to 4 survey questionnaires in 1975, 1981, 1990 and 2011 and reported their weight and height used to calculate BMI (kg/m^2^).^33^ A fifth wave of data was derived from a subset of the twins born 1945 to 1957 who participated in the Essential Hypertension Epigenetics (EH‐Epi) study. Twin pairs discordant for blood pressure were invited to take part in the EH‐Epi study, as described in detail earlier.^34^ During 2012–2014, research nurses conducted interviews and measured height, weight and waist circumference. They were also asked about their current medication usage and the purpose for which they had been prescribed, coded using the Anatomical Therapeutic Chemical Classification System (ATC). This information was used to determine whether participants used anti‐hypertensive medication (yes/no). Physical activity and sedentary behaviour during work, commuting and leisure were assessed through 14 questions. The participants completed a brief food‐frequency questionnaire. The first item on the questionnaire asked for their typical portion size in relation to a typical 400 g ready‐to‐eat dish size, which is commonly used in Finland. A series of pictures was used to illustrate alternatives (see supplement for the question and the response alternatives). The respondent was asked to indicate whether they ate small portions (a third of the standard dish), moderate portions (two‐thirds size), fairly large portions (400 g) or large portions (4/3 of the standard dish).

Fasting venous blood samples were collected from the EH‐Epi twins at a mean age of 62 (age range: 55–69 years) to generate proteomic and genetic data for genome‐wide analyses. Participants with complete BMI measurements for all five waves were selected for the current analyses. The correlation between measured and self‐reported BMI values was 0.95 for both men and women in the EH‐Epi study, showing high reliability of self‐reported BMI.^35^ A linear regression model with inclusion/exclusion as a binary independent variable was conducted to assess the randomness of participant selection (Supplementary Table S1). While BMI was not a criterion for selection, participants had lower BMI in the later surveys (3rd, 4th and 5th surveys) while in the earlier surveys (1st and 2nd surveys), their BMI was higher compared with the twins of the older FCT who were not selected for the current study. However, these differences were small and not statistically significant, suggesting that the EH‐Epi sample is broadly representative of the full cohort.

The research was conducted according to the principles of the Declaration of Helsinki, and the data collection was approved by the ethics committee of the Hjelt Institute, University of Helsinki and the ethics committee of the Helsinki and Uusimaa Hospital District, Finland.

### 2.2 Quantification of plasma proteins

The proteome was measured from plasma samples of 415 twins from the EH‐Epi using the Olink platform (Olink Explore 3072, Olink Proteomics AB, Uppsala, Sweden). A detailed description of the data and the pre‐processing steps can be found elsewhere.^8^ Briefly, the data are presented as Normalized Protein eXpression (NPX) values, where NPX is Olink's unit for quantifying relative protein concentrations on a log2 scale. Proteins detected in less than 80% of the samples were excluded. NPX values of remaining proteins that were below the limit of detection (LoD), representing less than 1% of all data points, were replaced with the LoD value of the corresponding plate. Outlier samples (*N* = 14) were identified using three distinct methods, as detailed previously.^8^ Each method was applied separately to individual protein panels^1^: Principal Component Analysis (PCA),^2^ assessment of the median and^3^ evaluation of the interquartile range (IQR) of NPX across proteins. Samples were excluded if they met any of the following criteria^1^: at least 1 of the first 2 standardized principal component values exceeded 5 standard deviations (SD) from the mean,^2^ the median NPX was more than 5 SD above or below the mean sample median or^3^ the NPX IQR was more than 5 SD above or below the mean IQR. Data were extracted from this sample for the current study participants, all of whom had complete proteomic data (*N* = 304). Only proteins from the Cardiometabolic (I/II) and Inflammation (I/II) panels were used (736 and 737 proteins in Cardiometabolic and Inflammation panels, respectively). Protein descriptions are shown in the supplementary material (Supplementary Table S2).

### 2.3 Polygenic risk score of BMI

PRS_BMI_ was calculated to test its interactions with changes and fluctuations in BMI during adulthood. The technical details of genotyping, imputation and PRS_BMI_ calculations have been described elsewhere.^36^ The PRS_BMI_ was derived using GWAS summary statistics for BMI.^29^ The total number of single nucleotide polymorphisms (SNPs) used for the PRS_BMI_ calculations was 996 919. The number of individuals included in the GWAS was 692 578. The PRS_BMI_ was regressed against the top ten genetic principal components to correct for population stratification.^37^ The residuals were obtained and scaled to a mean of zero and unit variance, and subsequently used in relevant analyses without further processing. The distribution of PRS_BMI_ values for the sample used (consisting of individuals with BMI measurements from the 5 surveys conducted; *N* = 304) was similar to the distribution of PRS_BMI_ values in the Older FTC (those not meeting the criteria for having BMI measurements from all 5 surveys; *N* = 143) (see Supplementary Figures S1 PRS_BMI_ of included (A) and excluded (B) samples, respectively).

### 2.4 Calculation of changes in BMI

Linear mixed‐effects (LME) modelling, i.e., models that include both fixed and random effects,^38^ was applied to longitudinal BMI data from the five surveys (1975, 1981, 1990, 2011 and 2012–2014) to calculate the linear trends and changes in BMI over time. LME models first estimate a general intercept and slope that apply to all participants, representing the average trend at the population level. However, individuals vary in both their baseline BMI and the rate at which they gain weight over time. To account for this variability, we included random effects on both the intercept and slope, allowing each participant to deviate from the fixed population‐level estimates. This resulted in individual‐specific intercept (i.e., fitted baseline BMI) and slope (i.e., rate of change) values. The analyses were performed using R software (version 4.2.3) packages lme4 (version 1.1–34), lmerTest (version 3.1–3), modelsummary (version 1.4.3), dplyr (version 1.1.4) and optimx (version 2023–10.21).

### 2.5 Calculation of the fluctuation in BMI

The expected BMI for each measurement point was calculated using the formula *y = xn + m*, where *n* is the change in BMI expressed as kg/m^2^ per year (i.e., slope), *m* is the baseline BMI (i.e., BMI at the first measure in 1975; age range 18–30 years) and *x* is the difference between the age of interest and the baseline age. Once the expected BMI values at each time point had been obtained, the difference between each of these expected BMI values and the corresponding observed BMI values was calculated. Finally, the differences between the observed and fitted BMI values were squared, and the resulting values were then averaged.

### 2.6 Associations between proteins, changes or fluctuations in BMI and PRS_BMI_

LME models were used to investigate the associations between BMI changes and fluctuation with protein levels. This was achieved through the implementation of two distinct models (Models displayed in the caption of the Table 2 and supplementary Table S3). We then assessed whether changes or fluctuation in BMI were associated with PRS_BMI_. The subsequent step was to examine interactions between protein levels and PRS_BMI_ in the case of a significant association in previous analyses using the same model as the one for assessing the associations between changes or fluctuation in BMI and PRS_BMI_ (models displayed in the caption of Supplementary Table S6), but with the addition of a protein and the interaction between the protein and PRS_BMI_ as fixed effects. Next, interactions between protein levels and BMI at baseline (i.e., BMI at age range 18–30 years) were also examined. For proteins found to be associated with BMI changes and fluctuation, the Reactome pathway database^39^, ^40^ was utilised to examine which pathways they belong to (pathways with an adjusted *p* value (FDR) < 0.05 were considered significant).

Finally, we conducted sensitivity analyses. For analyses focusing on changes in BMI, we first repeated them after excluding individuals with BMI > 30 kg/m^2^ in any of the surveys. This was done to investigate whether the identified associations were not limited to the participants with obesity. Secondly, we conducted an analysis of the associations between BMI changes and proteomics, taking into account several potential confounders: (i) hypertension (having anti‐hypertensive medication or not) due to the high percentage of individuals with hypertension (about 60% in both sexes), (ii) physical activity and (iii) diet (in terms of usual portion size of food), since they are known to be associated with the development of obesity.^41^, ^42^ For analyses focusing on BMI fluctuation, BMI trajectories (i.e., slope and intercept) were incorporated as covariates into the models to assess whether the significant associations between BMI fluctuation and plasma proteins were attenuated when the BMI trajectories were taken into account. In all LME models used to examine associations between variables, random effects were applied to family IDs. This allowed us to account for the dependence of observations in the data due to family relatedness.

All the analyses were performed using the R software (version 4.2.3) and the R package lme4 (version 1.1–34). The models used are displayed in the caption of the main and supplementary tables.

### 2.7 Within‐pair analysis

To assess the influence of environmental factors and to rule out the effect of genetic differences on the significant associations between changes and fluctuation in BMI with proteins, within‐pair analyses were conducted. These analyses were carried out using MZ twin pairs only (N of pairs = 50). Since MZ twins in a pair share, in practice, identical DNA sequences, any differences in their BMI, rate of BMI change or BMI fluctuation suggest environmental influence. For example, if MZ twins who experience greater weight gain than their co‐twins also exhibit higher levels of a specific protein, this association would likely be unrelated to genetic factors. We calculated within‐pair differences (referred to with a ∆ symbol below) between the changes in BMI, BMI fluctuation and the protein levels we previously identified as significantly associated with these two variables. We also calculated the mean of the baseline BMI values for each pair.

All the analyses were performed using the R software (version 4.2.3) and the R package lme4 (version 1.1–34), and the models used are displayed in the caption of the main and supplementary tables.

## 3 RESULTS

### 3.1 Cohort and BMI trajectories

A total of 305 Finnish twins (118 men) with complete BMI and proteomic information were included in the current study. The mean BMI increased over the years in both men (mean of 5.27 kg/m^2^) and women (mean of 5.73 kg/m^2^). This corresponds to an annual increase of 0.13 kg/m^2^ in men and 0.14 kg/m^2^ in women, equating to around 400 g weight gain per year for a 170 cm tall person. We observed sex differences in BMI, height and weight, as well as changes in BMI from the earliest stages of the follow‐up, and this pattern maintained throughout (Table 1). Baseline BMI (i.e., intercept) and changes in BMI over the 40‐year follow‐up period (i.e., slope) were positively correlated in both men (r = 0.22, p value = 0.01) and women (r = 0.23, p value <0.01). Regarding the results of the classification of individuals into different BMI categories, we found that 31% of participants were of normal weight, 48% were overweight and 21% were classified as obese. See Table 1 for the distribution by sex. The proportions of participants with prescribed anti‐hypertensive medication were similar in men (57%) and women (56%). The longitudinal changes in BMI are illustrated in Supplementary Figure S2.

**TABLE 1:** Descriptive and trajectory information of Older Finnish Twin Cohort by sex.

	Men (*n* = 118)		Women (*n* = 186)		*p* value
	Mean	SD	Mean	SD	
Wave 1 (1975)					
Age	24	3,65	24	3,73	0,57
Height	1,78	0,06	1,63	0,05	<2,00E‐16
Weight	70	10,82	55	6,9	<2,00E‐16
BMI	22,34	2,84	20,24	2,53	1,10E‐09
Wave 2 (1981)					
Age	30,11	3,6	30,13	3,75	0,95
Height	1,78	0,06	1,63	0,05	<2,00E‐16
Weight	75,5	11,67	56	7,18	<2,00E‐16
BMI	23,63	3,1	21,06	2,79	3,41E‐09
Wave 3 (1990)					
Age	39,34	3,54	38,92	3,75	0,22
Height	1,78	0,06	1,63	0,05	<2,00E‐16
Weight	80	13,01	60	8,82	<2,00E‐16
BMI	24,89	3,5	22,22	3,39	1,92E‐10
Wave 4 (2011)					
Age	60,15	3,59	60,14	3,81	0,86
Height	1,78	0,06	1,62	0,05	<2,00E‐16
Weight	84,5	14,44	67	11,43	<2,00E‐16
BMI	26,75	4,01	24,91	4,31	3,78E‐05
Wave 5 (2015)					
Age	62,19	3,83	62,41	3,83	0,08
Height	1,77	0,07	1,61	0,05	<2,00E‐16
Weight	86,25	15,27	68	12,96	<2,00E‐16
BMI	27,61	4,51	25,97	5,11	4,34E‐03
BMI trajectories					
Slope	0,13	0,06	0,14	0,08	4,28E‐04
Intercept	22,54	2,84	20,73	2,53	4,79E‐08
BMI categories	***N***	**%**	***N***	**%**	**Total *N* (Total %)**
Normal	25	21%	70	37%	95 (31%)
Overweight	58	50%	87	47%	145 (48%)
Obese	34	29%	30	16%	64 (21%)

*Note*: Age, height, weight and BMI mean values for each wave along with BMI trajectories (i.e., Slope and Intercept) are displayed along with the standard deviation, and the p value obtained from t‐test to assess sex differences.

Abbreviation: SD, standard deviation.

### 3.2 Associations between proteins and changes in BMI

After correcting significance levels for multiple testing using the Bonferroni method (number of tests = 1231, corrected p value = 4,06 e‐05), a total of 135 out of 1231 proteins were significantly associated with changes in BMI: 73/736 proteins belonged to the cardiometabolic panel and 62/737 proteins to the inflammation panel. A negative association with changes in BMI was observed for 31 proteins, while a positive association was observed for 104 proteins (Supplementary Table S3). The strongest negative association was observed with the protein Apolipoprotein F (coefficient: −0.14; *p* = 5.88e‐10), indicating that one unit increase in apolipoprotein F expression was associated with a 0.14 kg/m^2^ decrease in changes in BMI per year. The strongest positive association was observed between changes in BMI and the expression of the Pigment epithelium‐derived factor (coefficient: 0.18; *p* = 1.86e‐11), indicating that a one unit increase in Pigment epithelium‐derived factor expression was associated with a 0.18 kg/m^2^ increase in BMI changes per year. The full summary is available in the supplementary material (Supplementary Table S3). Four different pathways were significantly related to the proteins associated with BMI changes. The first pathway was regulation of Insulin‐like Growth Factor (IGF) transport and uptake by Insulin‐like Growth Factor Binding Proteins (IGFBPs) (FDR of entities = 1.18e‐05), where 12 different proteins were found to be involved in this pathway. For the proteins associated with BMI fluctuation, two were identified. The pathway with the strongest significance was the transcriptional regulation of white adipocyte differentiation (FDR of entities = 0.01) where 3 proteins were found to be involved in this pathway.

In the sensitivity analyses excluding participants with BMI values greater than 30 kg/m^2^ in any of the surveys, the number of proteins associated with changes in BMI decreased to 40. Approximately half of these proteins belonged to the cardiometabolic panel, and the other half belonged to the inflammatory panel (19 and 21 proteins, respectively). Pigment epithelium‐derived factor remained the protein showing the strongest positive association with changes in BMI (coefficient: 0.11; Bonferroni *p* = 9.90e‐04), while serum Paraoxonase/Lactonase 3 was the protein with the strongest negative association (coefficient: −0.09; Bonferroni *p* = 8.41e‐05) (Supplementary Table S5). Additionally, analysis including anti‐hypertensive medication (yes or no), physical activity and diet as confounders resulted in 116 proteins that remained significantly associated with BMI changes. From these 116 proteins, approximately half belonged to the cardiometabolic panel, and the other half belonged to the inflammatory panel (63 and 53 respectively). Results are displayed in Supplementary Table S6.

### 3.3 Associations between proteins and fluctuation of BMI

Fluctuation of BMI over time was associated with 17 proteins: 10 belonged to the inflammation panel and 7 to the cardiometabolic panel (Table 2). All proteins were found to be positively associated with BMI fluctuation, with the strongest association observed for Interleukin‐10 receptor subunit beta (coefficient: 1.50; *p* = 1.61e‐02). The pathway enrichment analysis results found two different pathways with significant results for the proteins that were associated with BMI fluctuation (Supplementary Table S7). The most important was the transcriptional regulation of white adipocyte differentiation (FDR of entities = 0.01) where 3 different proteins were found to be involved in this pathway. In a sensitivity analysis, where BMI slope and BMI intercept were included as covariates, the associations were non‐significant (Supplementary Table S8).

**TABLE 2:** Proteins showing a significant association with fluctuation in BMI after carrying out simple linear mixed effects (LME).

Protein Description	Protein ID	Estimate	SE	*p* value		Protein panel
				Nominal	Bonferroni	
Leptin	P41159	0,46	0,08	2,30E‐08	2,84E‐05	Cardiometabolic
Interleukin‐1 receptor antagonist protein	P18510	0,7	0,12	2,41E‐08	2,97E‐05	Inflammation
Fatty acid‐binding protein, adipocyte	P15090	0,8	0,15	3,61E‐07	4,45E‐04	Cardiometabolic
Angiopoietin‐related protein 4	Q9BY76	1,13	0,23	9,69E‐07	1,19E‐03	Inflammation
Disintegrin and metalloproteinase domain‐containing protein 12	O43184	0,94	0,19	1,99E‐06	2,45E‐03	Inflammation
Na(+)/H(+) exchange regulatory cofactor NHE‐RF3	Q5T2W1	0,59	0,13	3,55E‐06	4,37E‐03	Inflammation
Growth/differentiation factor 15	Q99988	1,03	0,22	3,85E‐06	4,74E‐03	Cardiometabolic
Bile salt sulfotransferase	Q06520	0,51	0,11	8,00E‐06	9,86E‐03	Inflammation
2‐iminobutanoate/2‐iminopropanoate deaminase	P52758	0,62	0,14	8,24E‐06	1,02E‐02	Inflammation
Coiled‐coil domain‐containing protein 80	Q76M96	1,03	0,23	8,33E‐06	1,03E‐02	Cardiometabolic
Interleukin‐10 receptor subunit beta	Q08334	1,5	0,34	1,30E‐05	1,61E‐02	Inflammation
Aflatoxin B1 aldehyde reductase member 4	Q8NHP1	0,43	0,1	1,41E‐05	1,74E‐02	Inflammation
Pterin‐4‐alpha‐carbinolamine dehydratase	P61457	0,67	0,15	1,71E‐05	2,11E‐02	Inflammation
Scavenger receptor cysteine‐rich type 1 protein M130	Q86VB7	0,85	0,19	1,74E‐05	2,14E‐02	Cardiometabolic
Pantetheinase	O95497	0,53	0,12	2,05E‐05	2,52E‐02	Inflammation
Glutathione S‐transferase A1	P08263	0,34	0,08	2,27E‐05	2,79E‐02	Cardiometabolic
E‐selectin	P16581	0,68	0,16	3,60E‐05	4,43E‐02	Cardiometabolic

*Note*: Significant association results displayed from the use of LME model: BMI_fluctuation ~ Sex + Age at blood sample + Protein + (1|Family ID) with the fluctuation in BMI as an outcome, the protein level, sex and the age when the blood sample was taken as fixed effects. Estimate, standard error, nominal p value and p value after Bonferroni correction are displayed for the proteins (characterised by the protein ID, protein description and belonging panel) that showed significant association with BMI fluctuation.

Abbreviation: SE: Standard error.

### 3.4 Associations between PRS_BMI_ with BMI changes and fluctuation of BMI

The associations between changes and fluctuation in BMI and the PRS_BMI_ were quantified using LME models. PRS_BMI_ was positively associated with changes in BMI (estimate: 0.01, p value = 0.01) but not with fluctuation in BMI (Supplementary Table S9).

### 3.5 BMI changes–protein associations with interactions

**FIGURE 1:**
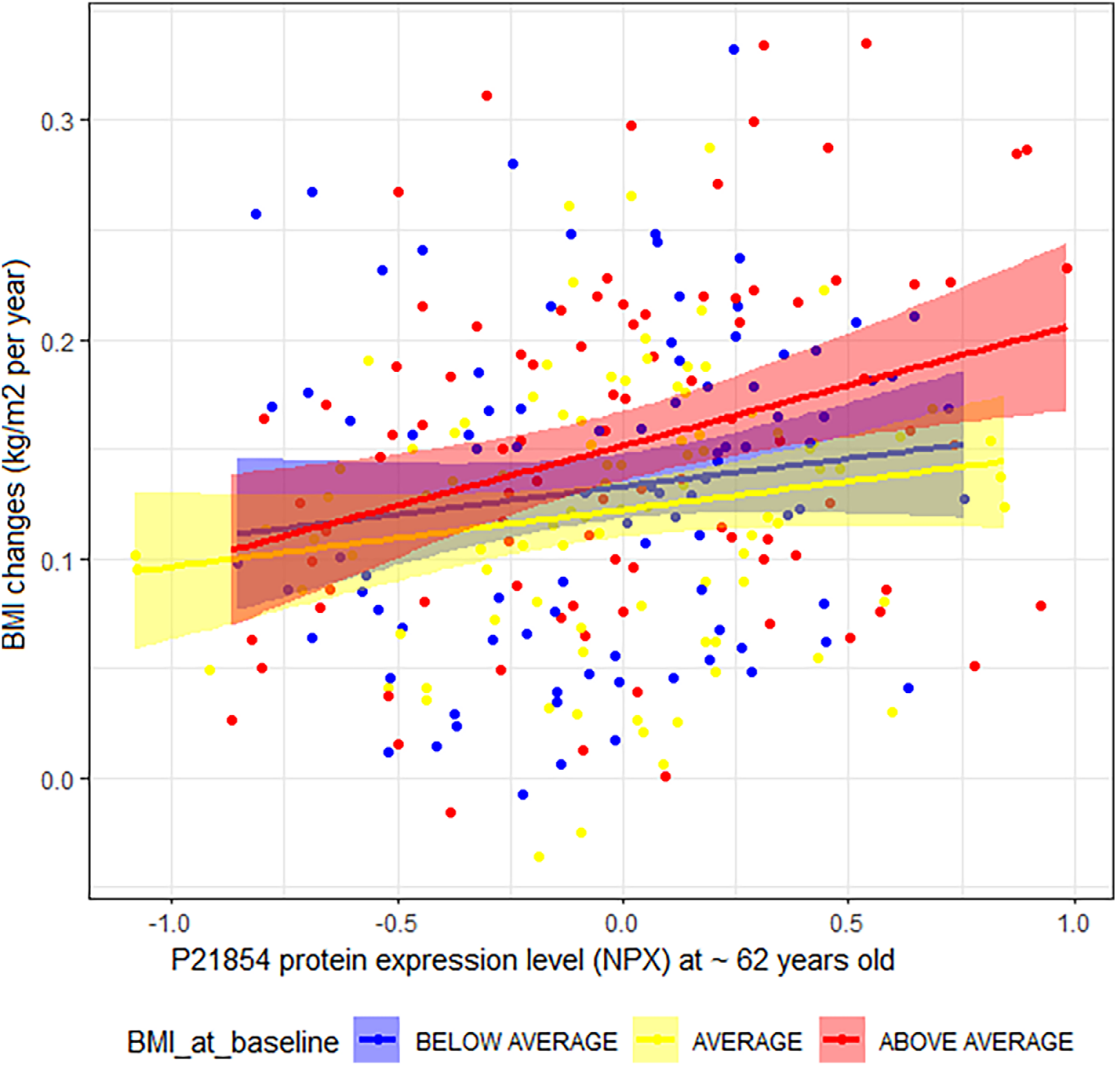
Graphical illustration of interactions between plasma protein levels with BMI baseline in predicting BMI changes during adulthood. Graphical illustration of the association between changes in BMI during adulthood (from ~24 to ~62 years of age) and the interaction between protein B cell differentiation antigen CD72 and BMI baseline (at ~24 years old) (interaction coefficient: 0.01, p = 0.05).

After introducing an interaction term between PRS_BMI_ and proteins associated with changes in BMI, no significant interaction was found. However, when investigating whether baseline BMI and protein levels interact in predicting changes in BMI, one significant interaction was identified (Table 3 and Figure 1). This interaction involved the B cell differentiation antigen CD72 protein with a positive estimate of 0.01 (Bonferroni *p* = 0.05). We did not perform interaction analyses on BMI fluctuation, since we did not identify any associations between BMI fluctuation and PRS_BMI_.

**TABLE 3:** Linear mixed‐effects models to assess associations of BMI changes with interactions between the BMI baseline and the proteins that previously appeared to be significantly associated with the changes in BMI.

Protein ID	Protein description	BMI baseline				Protein				BMI baseline:protein			
		Estimate	SE	*p* value	Bonferroni	Estimate	SE	*p* value	Bonferroni	Estimate	SE	*p* value	Bonferroni
P21854	B cell differentiation antigen CD72	0.004	0.001	7.47E‐03	1.000	−0.200	0.07	4.58E‐03	0.610	0.010	0.003	4.26E‐04	0.05

*Note*: Significant association results displayed from the use of LME model: Change_BMI ~ Sex + Age at blood sample + Protein + Protein:Baseline_BMI + Baseline_BMI + (1|Family ID) where changes in BMI are the outcome and the protein level at PRS ~ 62 years old, the age of blood sampling the baseline BMI (~24 years old) and the interaction between protein level and the baseline BMI were fixed effects.

Abbreviation: SE: Standard error.

### 3.6 Within‐pair analyses

Ten proteins associated with BMI change showed significant associations within MZ twin pairs: seven were identified as part of the cardiometabolic panel (Leptin, High affinity immunoglobulin alpha and Immunoglobulin mu Fc receptor, IGF‐binding protein 2, Creatine kinase B‐type, Somatotropin, BPI fold‐containing family B member 1 and IGF‐binding protein 1) and three as part of the inflammation panel (Apolipoprotein F, Growth hormone receptor and Ectonucleotide pyrophosphatase/phosphodiesterase family member 7) (Supplementary Table S10). The protein with the most robust positive association was Growth hormone receptor (coefficient: 0.14; *p* = 4.00e‐3), while the one with the most pronounced negative association was Apolipoprotein F (coefficient: −0.31; *p* = 1.61e‐3). Only 2 of the 17 proteins associated with BMI fluctuation showed significant associations in within‐pair analyses (see Supplementary Table S11). These proteins were E‐selectin and Na(+)/H(+) exchange regulatory cofactor NHE‐RF3 protein.

## 4 DISCUSSION

The current study aimed to investigate (i) the associations between changes in BMI and BMI fluctuation over time during adulthood with plasma proteins, (ii) whether these associations persisted after controlling for shared confounding between MZ co‐twins and (iii) whether the associations between changes and fluctuation in BMI with protein levels are modulated by PRS_BMI_. Overall, 135 proteins were associated with changes in BMI, while 17 were associated with fluctuation in BMI. When conducting within‐pair analyses in MZ pairs, 10 and 2 proteins remained significantly associated with BMI changes and BMI fluctuation, respectively. These results suggest that genetic factors are not the sole drivers of the observed associations, but rather highlight the importance of the environment not shared by co‐twins. In addition, PRS_BMI_ was significantly associated with BMI changes. Nevertheless, no significant interactions between PRS_BMI_ and protein levels in explaining changes in BMI were observed. However, we identified one significant interaction between baseline BMI and the B cell differentiation antigen CD72 protein in explaining changes in BMI. This indicates that the association between the cell differentiation antigen CD72 protein and changes in BMI was stronger in individuals whose baseline BMI was high, independent of the direct effect of baseline BMI on BMI change.

Several proteins were identified as being associated with BMI changes during adulthood. Some of these proteins, including those belonging to the Complement factors (specifically Complement factor I, B, D and H), Sex Hormone Binding Protein (SHBP), Galectin‐3 binding protein (Gal‐3BP), Afamin and Antithrombin III, have been documented in intervention studies focusing on proteome changes after weight loss in individuals with overweight or obesity as a starting point.^13^, ^14^, ^15^, ^16^

Although the proteins found in the current study matched those in previous studies, the direction of the association was different from that found in our work. For instance, in several studies, the authors observed that Complement factors, Gal‐3BP and Afamin were negatively associated with weight loss, while SHBP and Antithrombin III were positively associated with weight loss.^13^, ^14^, ^15^, ^16^ In contrast, our study found opposite directions in these associations with weight gain, which corroborates findings from intervention studies on weight loss.

Other proteins, such as IGFBPs (*IGFBP1* and *IGFBP2*) and serum amyloid P, have also been reported in the literature on weight loss, although with less frequency. In the case of IGFBPs, previously published studies demonstrated a positive association between these proteins and weight loss.^15^, ^16^ Our findings indicate negative correlations between these proteins and changes in BMI. Regarding serum Amyloid P, previous studies reported a negative association between this protein and weight loss.^13^, ^16^ However, we found a positive association with changes in BMI. In both cases (the IGFBPs and serum Amyloid P), the results are consistent, as the direction of BMI change aligns.

Finally, when comparing our results with a previous longitudinal study that examined plasma proteome changes over a 10‐year period, several reported proteins were also identified in the current study as being associated with changes in BMI over a period of 40 years.^43^ These proteins include Leptin, Tissue‐type plasminogen activator, Cathepsin D and Hepatocyte Growth Factor (HGF), all of which exhibited a positive association with BMI changes.^43^ This is consistent with the findings of the current study, indicating that these proteins are likely good candidate biomarkers for long‐term weight gain in adults. Additionally, another previously conducted longitudinal study where the long‐term associations between BMI changes during adolescence and protein levels were examined found some Complement factors (I, B and H), a serum Amyloid P component and SHBP to be associated with the BMI changes.^44^ Those proteins exhibited similar patterns of association to those observed in the current study. Since weight gain in adults is mainly in fat mass, this suggests that the above proteins may be associated with changes in body fat rather than lean mass during adolescence.

We identified several proteins associated with BMI fluctuation. Most of them (except for Fatty acid‐binding protein, Adipocyte, 2‐iminobutanoate/2‐iminopropanoate deaminase, Aflatoxin B1 aldehyde reductase member 4 and Pantetheinase) were previously reported to be associated with weight loss.^16^ The majority of the proteins showed a negative association with weight loss, except for Na(+)/H(+) exchange regulatory cofactor NHE‐RF3, Coiled‐coil domain‐containing protein 80 and Pterin‐4‐alpha‐carbinolamine dehydratase, which exhibited a positive association. A previously published intervention study also identified a negative association between Pantetheinase and weight loss.^15^ Additionally, a few of these proteins (Leptin and E‐selectin) were identified as positively associated with long‐term changes in BMI in a previously published study.^16^ However, to the best of our knowledge, no previous studies have examined the associations between longitudinal BMI fluctuation and protein levels, which hinders our ability to compare our results with existing literature on BMI fluctuation. Further studies investigating the proteomic associations with BMI fluctuation are warranted.

The pathway enrichment analysis suggests that proteins associated with BMI fluctuation may be collectively involved in metabolic regulation, adipose tissue differentiation and immune signalling. Growth factor pathways, particularly IGFs, regulate nutrient metabolism, energy utilization and fat storage, highlighting their role in body composition changes.^45^, ^46^ Additionally, post‐translational modifications, such as protein phosphorylation, fine‐tune metabolic responses, thus affecting Insulin sensitivity and energy homeostasis.^47^ Moreover, the enrichment of immune‐related pathways suggests a link between inflammation and metabolism, where cytokine signalling and complement activation may contribute to adipose tissue function and BMI fluctuation.^11^ Chronic low‐grade inflammation is known to influence fat accumulation, Insulin resistance and metabolic disorders, reinforcing the interplay between metabolic and immune processes in weight regulation.^48^ These results are also reinforced by the findings of previous studies on the link between inflammation and the development of obesity.^49^, ^50^, ^51^ However, evidence linking these pathways to the observed associations between proteins and BMI fluctuation remains limited, as only a few proteins have been identified in relation to these pathways.

From the previously identified associations between proteins with changes or fluctuation in BMI, several remained significant in within‐pair analyses conducted in MZ twin pairs. This suggests that some associations between protein levels and BMI trajectories persist when controlling for genetic factors, indicating either an environmental influence on these associations or possible causal relationships. Environmental correlations between protein levels and BMI as well as changes in BMI during adolescence have been previously reported,^42^ and our study complements the literature with a longer follow‐up over adulthood. Additionally, to our knowledge, no previous study has investigated the environmental effects underlying associations between the plasma proteome and fluctuation in BMI. We demonstrate that two proteins are associated (Na(+)/H(+) exchange regulatory cofactor NHE‐RF3 and E‐selectin) with fluctuation in BMI independently of genetic factors.

Finally, only one interaction between baseline BMI and protein levels was positive and significant in predicting changes in BMI. This interaction involved the protein B cell differentiation antigen CD72. In individuals with the same protein level, those with a higher BMI at baseline gained more BMI during adulthood, in addition to the individual effect of baseline BMI on changes in BMI. To the best of our knowledge, no previous studies have reported the B cell differentiation antigen CD72 protein as being associated with changes in BMI alone, or as interacting with baseline BMI.

Our study has several strengths. The longitudinal data derived from a twin cohort with five BMI measurements enabled the characterization of BMI trajectories with a high level of depth. To the best of our knowledge, our study represents the longest follow‐up period of any proteomic study investigating changes and fluctuations in BMI to date. However, it is important to note that our study faces limitations. One potential limitation of our study is the representativeness of the sample, as the last on‐site visit (5th wave) was designed to include twin pairs discordant for blood pressure. However, recruitment was not restricted to such pairs, and discordance does not imply that the EH‐Epi twins have higher blood pressure than the general population. A recent study on the history of hypertension in Finland reported that over 50% of individuals in their sample, born in the 1940s, used anti‐hypertensive medication—a prevalence similar to that observed in the EH‐Epi sample.^52^ Moreover, the measured blood pressure values in our twins align closely with those reported in the FINRISK 2012 study^53^ for the same age group population estimates (Supplementary Table S13). This suggests that, despite the recruitment strategy, the EH‐Epi twins reasonably represent their age group in the population. Another limitation is the relatively small sample size, which may have reduced the statistical power of the study and increased the uncertainty of the obtained estimates. Furthermore, since protein levels were measured only once, we could not study temporal changes in protein expression. Additionally, BMI is a measure that is less informative about a person's obesity status than other measures such as body fat mass or adiposity. Nevertheless, the collection of more sophisticated measurements of body fat is challenging within the scope of long‐term observational studies. Finally, although we included anti‐hypertensive medication, physical activity and diet as covariates in the sensitivity analysis, other potential factors may confound associations. However, we conducted within‐pair analyses which, by design, correct for unmeasured genetic confounding and unknown environmental factors shared by the co‐twins. Thus, significant associations identified in within‐pair analyses may be of interest for further causal and biomarker investigations.

## 5 CONCLUSION

The current study identified numerous associations between long‐term changes and fluctuations in BMI with various cardiometabolic and inflammation‐related protein levels. These proteins may be of interest to identify individuals whose BMI may increase sharply over time. Our findings suggest that genetic factors are not the only drivers of the associations between the blood plasma proteome and BMI trajectories but highlight the role of the environment in shaping long‐term BMI change and its link with the older age proteome. From a clinical perspective, these results could assist in the development of personalised treatment strategies targeting specific metabolic pathways to prevent unhealthy fluctuations in BMI. In public health, the early identification of at‐risk individuals using protein biomarkers could aid in designing targeted lifestyle and dietary interventions to mitigate the long‐term consequences of weight cycling. Nevertheless, since this study focused on adults, more longitudinal studies of weight change and the associated proteins in children and adolescents are needed. Follow‐up studies starting at earlier ages would enable the identification of proteins that could serve as markers for monitoring and preventing the development of obesity from a young age.

## AUTHOR CONTRIBUTIONS

The study design was developed and discussed by AO, GD and JK. The statistical analyses were performed by AO. The phenotype data was processed by AO and the proteomic data processing was performed by GD. JK, XW and MO participated in the data collection. Polygenic risk scores were calculated by TP. AO wrote the original manuscript. AO, GD, JK, XW, MO, TP and KS participated in the improvement of the manuscript by critically revising it and read and approved the final version of the manuscript.

## FUNDING INFORMATION

AO, KS and JK have been supported by the European Union's Horizon Europe Research and Innovation programme under Grant Agreement number 101080117 (BETTER4U). Views and opinions expressed are, however, those of the author(s) only and do not necessarily reflect those of the European Union. Neither the European Union nor the granting authority can be held responsible for them. The FTC has been supported by the Academy of Finland (grants 312073 & 336832 to Jaakko Kaprio and 297908 to Miina Ollikainen) and the Sigrid Juselius Foundation (to Miina Ollikainen). The Essential Hypertension Epigenetics study was supported by NIH/NHLBI grant HL104125 to Xiaoling Wang.

## CONFLICT OF INTEREST STATEMENT

The authors declared no potential conflicts of interest with respect to the research, authorship and /or publication of this article.

## PEER REVIEW

The peer review history for this article is available at https://www.webofscience.com/api/gateway/wos/peer-review/10.1111/dom.16448.

## Supporting information


**Figure S1.** Graphical illustration of the distribution of the Polygenic Risk Score of Body Mass Index (A) of the samples included in the study and (B) of the samples not included in the study. Density plot of the Polygenic Risk Score of Body Mass Index of the individuals of the older Finnish twin cohort (A) included in the study (*N* = 305) and (B) not included in the study(*N* = 175). BMI: body mass index; PRS: Polygenic risk score.

**Figure S2.** Spaghetti plot of individual BMI changes over time. Caption: BMI has been self‐reported at 5 different waves comprising a 40‐year period.

**Table S1.** Linear regression model to assess selection bias based on the Body masss index.
**Table S2.** List of proteins used in the current study obtained from Olink® Explore.
**Table S3.** Linear mixed‐effects models to assess associations of proteins at ~62 years old with BMI changes during adulthood (from 24 to 62 years old).
**Table S4.** Significant biological pathways of the proteins at ~62 years old significantly associated with BMI changes during adulthood (24 to 62 years old).
**Table S5.** Linear mixed‐effects models to assess associations of proteins at ~62 years old with BMI changes during adulthood (from 24 to 62 years old) including only individuals with BMI less than 30 kg/m^2^.
**Table S6.** Linear mixed‐effects models to assess associations of proteins at ~62 years old with BMI changes during adulthood (from 24 to 62 years old) including the intake of anti‐hypertensive medication, physical activity and diet as a covariate.
**Table S7.** Significant biological pathways of the proteins at ~62 years old significantly associated with BMI fluctuations during adulthood (24 to 62 years old).
**Table S8.** Linear mixed‐effects models to assess associations of BMI fluctuation and proteins at ~62 years old including BMI slope and BMI intercept as covariates.
**Table S9.** Linear mixed‐effects models to assess associations of BMI changes and fluctuations with polygenic risk score (PRS) for BMI.
**Table S10.** Within‐pair analysis assessing which of the previously identified associations between proteins and BMI changes during adulthood remained significant when controlling for all genetic confounding.
**Table S11.** Within‐pair analysis assessing which of the previously identified associations between proteins and BMI fluctuation during adulthood remained significant when controlling for genetic confounding.
**Table S12.** Information on diet and physical activity in the included sample by sex and in total.
**Table S13.** Systolic and Diastolic blood pressure mean and SD in the sample of the study and FINRISK 2012 by sex.

## Data Availability

The Finnish Twin Cohort data used in the analysis is deposited in the Biobank of the Finnish Institute for Health and Welfare (https://thl.fi/en/web/thl-biobank/forresearchers). It is available to researchers after written application and following the relevant Finnish legislation.
