# Supplementary figures and images for: Proteomic associations with fluctuation and long‐term changes in BMI: A 40‐year follow‐up study
Source: Diabetes Obes Metab. 2025 May 8;27(8):4192–202. doi: 10.1111/dom.16448 (PMC12232349; doi:10.1111/dom.16448)

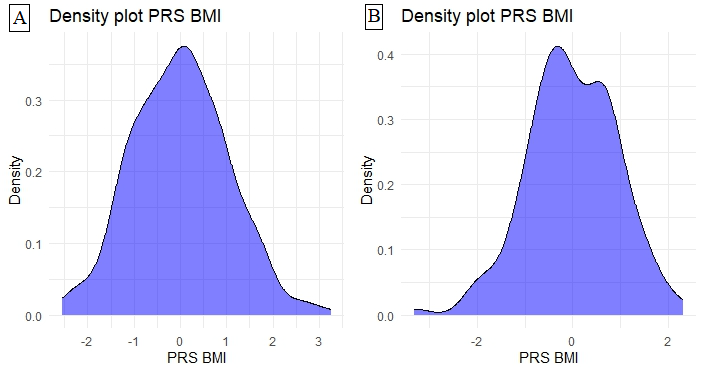

Supplement: Supplementary file 1 — Figure S1. Graphical illustration of the distribution of the Polygenic Risk Score of Body Mass Index (A) of the samples included in the study and (B) of the samples not included in the study. Density plot of the Polygenic Risk Score of Body Mass Index of the individuals of the older Finnish twin cohort (A) included in the study (N = 305) and (B) not included in the study(N = 175). BMI: body mass index; PRS: Polygenic risk score. [file DOM-27-4192-s002.tiff]

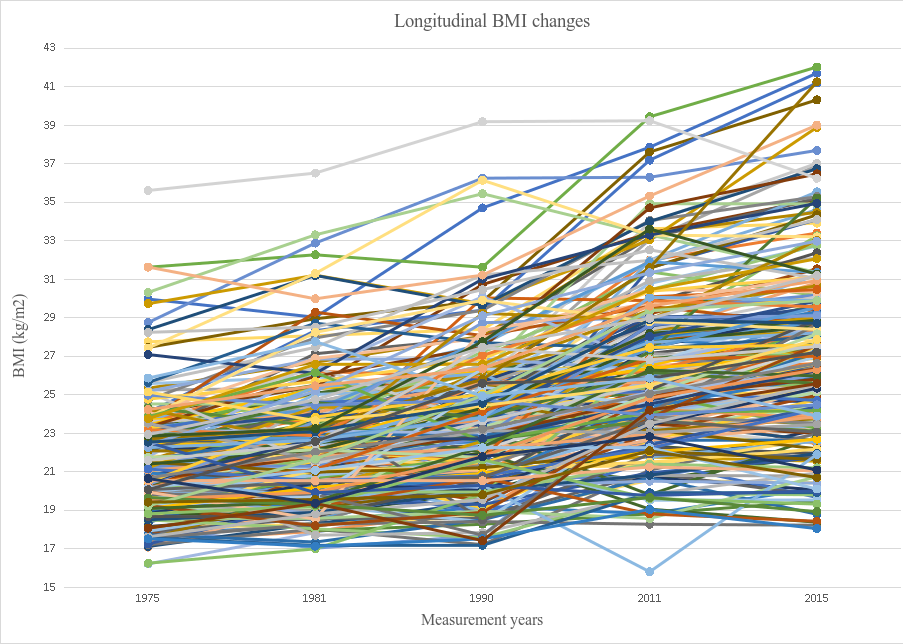

Supplement: Supplementary file 2 — Figure S2. Spaghetti plot of individual BMI changes over time. Caption: BMI has been self‐reported at 5 different waves comprising a 40‐year period. [file DOM-27-4192-s003.tiff]
