# Supplementary material for: Proteomic associations with fluctuation and long‐term changes in BMI: A 40‐year follow‐up study
Source: Diabetes Obes Metab. 2025 May 8;27(8):4192–202. doi: 10.1111/dom.16448 (PMC12232349; doi:10.1111/dom.16448)
Supplement: Supplementary file 3 — Table S1. Linear regression model to assess selection bias based on the Body masss index. Table S2. List of proteins used in the current study obtained from Olink® Explore. Table S3. Linear mixed‐effects models to assess associations of proteins at ~62 years old with BMI changes during adulthood (from 24 to 62 years old). Table S4. Significant biological pathways of the proteins at ~62 years old significantly associated with BMI changes during adulthood (24 to 62 years old). Table S5. Linear mixed‐effects models to assess associations of proteins at ~62 years old with BMI changes during adulthood (from 24 to 62 years old) including only individuals with BMI less than 30 kg/m2. Table S6. Linear mixed‐effects models to assess associations of proteins at ~62 years old with BMI changes during adulthood (from 24 to 62 years old) including the intake of anti‐hypertensive medication, physical activity and diet as a covariate. Table S7. Significant biological pathways of the proteins at ~62 years old significantly associated with BMI fluctuations during adulthood (24 to 62 years old). Table S8. Linear mixed‐effects models to assess associations of BMI fluctuation and proteins at ~62 years old including BMI slope and BMI intercept as covariates. Table S9. Linear mixed‐effects models to assess associations of BMI changes and fluctuations with polygenic risk score (PRS) for BMI. Table S10. Within‐pair analysis assessing which of the previously identified associations between proteins and BMI changes during adulthood remained significant when controlling for all genetic confounding. Table S11. Within‐pair analysis assessing which of the previously identified associations between proteins and BMI fluctuation during adulthood remained significant when controlling for genetic confounding. Table S12. Information on diet and physical activity in the included sample by sex and in total. Table S13. Systolic and Diastolic blood pressure mean and SD in the sample of the study and FINRIS [file DOM-27-4192-s001.docx]

**Supplementary table 1.** Linear regression model to assess selection bias based on the Body masss index.

| Linear Regression results | | | | |
| --- | --- | --- | --- | --- |
| BMI measure | Independent Variable | Estimate (kg/m2) | p value | R-squared |
| BMI 1975 | Inclusion | 0,10 | 0,72 | 0,16 |
| BMI 1981 | Inclusion | 0,35 | 0,33 | 0,17 |
| BMI 1990 | Inclusion | -1,34 | 0,08 | 0,12 |
| BMI 2011 | Inclusion | -0,53 | 0,27 | 0,03 |
| BMI 2015 | Inclusion | -0,62 | 0,26 | 0,01 |

**Caption:** Linear regression results from all the surveys with inclusion/exclusion as independent variable, with the estimate, the p value and the R^2^ values displayed. **Abbreviations:** BMI: Body mass index.

**Supplementary table 2**. List of proteins used in the current study obtained from Olink® Explore.

| **UniProt ID** | **Protein name** | **Gene name** | **Explore 384 panel** |
| --- | --- | --- | --- |
| P16860 | Natriuretic peptides B | NPPB | Cardiometabolic |
| P19429 | Troponin I, cardiac muscle | TNNI3 | Cardiometabolic |
| P61978 | Heterogeneous nuclear ribonucleoprotein K | HNRNPK | Cardiometabolic |
| P17676 | CCAAT/enhancer-binding protein beta | CEBPB | Cardiometabolic |
| P08670 | Vimentin | VIM | Cardiometabolic |
| O96017 | Serine/threonine-protein kinase Chk2 | CHEK2 | Cardiometabolic |
| P34998 | Corticotropin-releasing factor receptor 1 | CRHR1 | Cardiometabolic |
| Q969D9 | Thymic stromal lymphopoietin | TSLP | Cardiometabolic |
| P55082 | Microfibril-associated glycoprotein 3 | MFAP3 | Cardiometabolic |
| O95988 | T-cell leukemia/lymphoma protein 1B | TCL1B | Cardiometabolic |
| Q9NRD8 | Dual oxidase 2 | DUOX2 | Cardiometabolic |
| Q16619 | Cardiotrophin-1 | CTF1 | Cardiometabolic |
| P17516 | Aldo-keto reductase family 1 member C4 | AKR1C4 | Cardiometabolic |
| Q9NRV9 | Heme-binding protein 1 | HEBP1 | Cardiometabolic |
| P36952 | Serpin B5 | SERPINB5 | Cardiometabolic |
| P52789 | Hexokinase-2 | HK2 | Cardiometabolic |
| P34947 | G protein-coupled receptor kinase 5 | GRK5 | Cardiometabolic |
| Q96N03 | V-set and transmembrane domain-containing protein 2-like protein | VSTM2L | Cardiometabolic |
| P31483 | Nucleolysin TIA-1 isoform p40 | TIA1 | Cardiometabolic |
| Q9Y2B0 | Protein canopy homolog 2 | CNPY2 | Cardiometabolic |
| O43186 | Cone-rod homeobox protein | CRX | Cardiometabolic |
| O95183 | Vesicle-associated membrane protein 5 | VAMP5 | Cardiometabolic |
| Q6UWL2 | Sushi domain-containing protein 1 | SUSD1 | Cardiometabolic |
| Q9UKL0 | REST corepressor 1 | RCOR1 | Cardiometabolic |
| P01375 | Tumor necrosis factor | TNF | Cardiometabolic |
| P35218 | Carbonic anhydrase 5A, mitochondrial | CA5A | Cardiometabolic |
| P51161 | Gastrotropin | FABP6 | Cardiometabolic |
| Q15831 | Serine/threonine-protein kinase STK11 | STK11 | Cardiometabolic |
| O60635 | Tetraspanin-1 | TSPAN1 | Cardiometabolic |
| P62736 | Actin, aortic smooth muscle | ACTA2 | Cardiometabolic |
| P58546 | Myotrophin | MTPN | Cardiometabolic |
| O43854 | EGF-like repeat and discoidin I-like domain-containing protein 3 | EDIL3 | Cardiometabolic |
| Q13105 | Zinc finger and BTB domain-containing protein 17 | ZBTB17 | Cardiometabolic |
| P21246 | Pleiotrophin | PTN | Cardiometabolic |
| O95684 | Centrosomal protein 43 | CEP43 | Cardiometabolic |
| Q12912 | Inositol 1,4,5-triphosphate receptor associated 2 | IRAG2 | Cardiometabolic |
| P21964 | Catechol O-methyltransferase | COMT | Cardiometabolic |
| P09237 | Matrilysin | MMP7 | Cardiometabolic |
| Q15165 | Serum paraoxonase/arylesterase 2 | PON2 | Cardiometabolic |
| Q8TE57 | A disintegrin and metalloproteinase with thrombospondin motifs 16 | ADAMTS16 | Cardiometabolic |
| P55259 | Pancreatic secretory granule membrane major glycoprotein GP2 | GP2 | Cardiometabolic |
| Q92558 | Wiskott-Aldrich syndrome protein family member 1 | WASF1 | Cardiometabolic |
| Q99549 | M-phase phosphoprotein 8 | MPHOSPH8 | Cardiometabolic |
| Q8NHS0 | DnaJ homolog subfamily B member 8 | DNAJB8 | Cardiometabolic |
| Q9H5Y7 | SLIT and NTRK-like protein 6 | SLITRK6 | Cardiometabolic |
| O15354 | Prosaposin receptor GPR37 | GPR37 | Cardiometabolic |
| P20718 | Granzyme H | GZMH | Cardiometabolic |
| P13807 | Glycogen [starch] synthase, muscle | GYS1 | Cardiometabolic |
| P40225 | Thrombopoietin | THPO | Cardiometabolic |
| Q8NC01 | C-type lectin domain family 1 member A | CLEC1A | Cardiometabolic |
| O75354 | Ectonucleoside triphosphate diphosphohydrolase 6 | ENTPD6 | Cardiometabolic |
| P05231 | Interleukin-6 | IL6 | Cardiometabolic |
| P31997 | Carcinoembryonic antigen-related cell adhesion molecule 8 | CEACAM8 | Cardiometabolic |
| P25815 | Protein S100-P | S100P | Cardiometabolic |
| O75356 | Ectonucleoside triphosphate diphosphohydrolase 5 | ENTPD5 | Cardiometabolic |
| Q9BYF1 | Angiotensin-converting enzyme 2 | ACE2 | Cardiometabolic |
| P07585 | Decorin | DCN | Cardiometabolic |
| Q04760 | Lactoylglutathione lyase | GLO1 | Cardiometabolic |
| Q9NWQ8 | Phosphoprotein associated with glycosphingolipid-enriched microdomains 1 | PAG1 | Cardiometabolic |
| Q13444 | Disintegrin and metalloproteinase domain-containing protein 15 | ADAM15 | Cardiometabolic |
| P40818 | Ubiquitin carboxyl-terminal hydrolase 8 | USP8 | Cardiometabolic |
| P22004 | Bone morphogenetic protein 6 | BMP6 | Cardiometabolic |
| Q9UKP3 | Integrin beta-1-binding protein 2 | ITGB1BP2 | Cardiometabolic |
| P09668 | Pro-cathepsin H | CTSH | Cardiometabolic |
| P46379 | Large proline-rich protein BAG6 | BAG6 | Cardiometabolic |
| O14793 | Growth/differentiation factor 8 | MSTN | Cardiometabolic |
| Q9BWV1 | Brother of CDO | BOC | Cardiometabolic |
| P08319 | All-trans-retinol dehydrogenase [NAD(+)] ADH4 | ADH4 | Cardiometabolic |
| P09496 | Clathrin light chain A | CLTA | Cardiometabolic |
| P34913 | Bifunctional epoxide hydrolase 2 | EPHX2 | Cardiometabolic |
| Q96A56 | Tumor protein p53-inducible nuclear protein 1 | TP53INP1 | Cardiometabolic |
| Q9Y4X3 | C-C motif chemokine 27 | CCL27 | Cardiometabolic |
| P00568 | Adenylate kinase isoenzyme 1 | AK1 | Cardiometabolic |
| P09525 | Annexin A4 | ANXA4 | Cardiometabolic |
| Q15067 | Peroxisomal acyl-coenzyme A oxidase 1 | ACOX1 | Cardiometabolic |
| NTproBNP | N-terminal prohormone of brain natriuretic peptide | NTproBNP | Cardiometabolic |
| Q05315 | Galectin-10 | CLC | Cardiometabolic |
| Q6PJW8 | Consortin | CNST | Cardiometabolic |
| P48357 | Leptin receptor | LEPR | Cardiometabolic |
| P01222 | Thyrotropin subunit beta | TSHB | Cardiometabolic |
| P31949 | Protein S100-A11 | S100A11 | Cardiometabolic |
| Q9H7M9 | V-type immunoglobulin domain-containing suppressor of T-cell activation | VSIR | Cardiometabolic |
| O14917 | Protocadherin-17 | PCDH17 | Cardiometabolic |
| P16234 | Platelet-derived growth factor receptor alpha | PDGFRA | Cardiometabolic |
| Q12864 | Cadherin-17 | CDH17 | Cardiometabolic |
| Q9Y5X1 | Sorting nexin-9 | SNX9 | Cardiometabolic |
| Q16620 | BDNF/NT-3 growth factors receptor | NTRK2 | Cardiometabolic |
| Q03154 | Aminoacylase-1 | ACY1 | Cardiometabolic |
| O60496 | Docking protein 2 | DOK2 | Cardiometabolic |
| P21549 | Serine--pyruvate aminotransferase | AGXT | Cardiometabolic |
| Q13043 | Serine/threonine-protein kinase 4 | STK4 | Cardiometabolic |
| P21980 | Protein-glutamine gamma-glutamyltransferase 2 | TGM2 | Cardiometabolic |
| Q9UHL4 | Dipeptidyl peptidase 2 | DPP7 | Cardiometabolic |
| O60664 | Perilipin-3 | PLIN3 | Cardiometabolic |
| O94903 | Pyridoxal phosphate homeostasis protein | PLPBP | Cardiometabolic |
| Q9UMF0 | Intercellular adhesion molecule 5 | ICAM5 | Cardiometabolic |
| P10644 | cAMP-dependent protein kinase type I-alpha regulatory subunit | PRKAR1A | Cardiometabolic |
| P40189 | Interleukin-6 receptor subunit beta | IL6ST | Cardiometabolic |
| Q9H773 | dCTP pyrophosphatase 1 | DCTPP1 | Cardiometabolic |
| Q6GTS8 | N-fatty-acyl-amino acid synthase/hydrolase PM20D1 | PM20D1 | Cardiometabolic |
| Q53H82 | Endoribonuclease LACTB2 | LACTB2 | Cardiometabolic |
| Q13158 | FAS-associated death domain protein | FADD | Cardiometabolic |
| Q99674 | Cell growth regulator with EF hand domain protein 1 | CGREF1 | Cardiometabolic |
| P10145 | Interleukin-8 | CXCL8 | Cardiometabolic |
| P09382 | Galectin-1 | LGALS1 | Cardiometabolic |
| Q8WX77 | Insulin-like growth factor-binding protein-like 1 | IGFBPL1 | Cardiometabolic |
| P41218 | Myeloid cell nuclear differentiation antigen | MNDA | Cardiometabolic |
| P55285 | Cadherin-6 | CDH6 | Cardiometabolic |
| Q9UHD0 | Interleukin-19 | IL19 | Cardiometabolic |
| P16112 | Aggrecan core protein | ACAN | Cardiometabolic |
| Q9ULL4 | Plexin-B3 | PLXNB3 | Cardiometabolic |
| Q06418 | Tyrosine-protein kinase receptor TYRO3 | TYRO3 | Cardiometabolic |
| Q13541 | Eukaryotic translation initiation factor 4E-binding protein 1 | EIF4EBP1 | Cardiometabolic |
| O75340 | Programmed cell death protein 6 | PDCD6 | Cardiometabolic |
| P51693 | Amyloid-like protein 1 | APLP1 | Cardiometabolic |
| Q9NY25 | C-type lectin domain family 5 member A | CLEC5A | Cardiometabolic |
| P08263 | Glutathione S-transferase A1 | GSTA1 | Cardiometabolic |
| Q969P0 | Immunoglobulin superfamily member 8 | IGSF8 | Cardiometabolic |
| Q16820 | Meprin A subunit beta | MEP1B | Cardiometabolic |
| P18827 | Syndecan-1 | SDC1 | Cardiometabolic |
| P19022 | Cadherin-2 | CDH2 | Cardiometabolic |
| Q8NI22 | Multiple coagulation factor deficiency protein 2 | MCFD2 | Cardiometabolic |
| Q96LA6 | Fc receptor-like protein 1 | FCRL1 | Cardiometabolic |
| Q14956 | Transmembrane glycoprotein NMB | GPNMB | Cardiometabolic |
| P09417 | Dihydropteridine reductase | QDPR | Cardiometabolic |
| Q9UBU3 | Appetite-regulating hormone | GHRL | Cardiometabolic |
| P41236 | Protein phosphatase inhibitor 2 | PPP1R2 | Cardiometabolic |
| Q9Y5K6 | CD2-associated protein | CD2AP | Cardiometabolic |
| O95544 | NAD kinase | NADK | Cardiometabolic |
| P27352 | Cobalamin binding intrinsic factor | CBLIF | Cardiometabolic |
| Q9GZM7 | Tubulointerstitial nephritis antigen-like | TINAGL1 | Cardiometabolic |
| Q8WVQ1 | Soluble calcium-activated nucleotidase 1 | CANT1 | Cardiometabolic |
| Q8WTU2 | Scavenger receptor cysteine-rich domain-containing group B protein | SSC4D | Cardiometabolic |
| P78380 | Oxidized low-density lipoprotein receptor 1 | OLR1 | Cardiometabolic |
| Q9NR28 | Diablo homolog, mitochondrial | DIABLO | Cardiometabolic |
| Q86VZ4 | Low-density lipoprotein receptor-related protein 11 | LRP11 | Cardiometabolic |
| Q13275 | Semaphorin-3F | SEMA3F | Cardiometabolic |
| P41159 | Leptin | LEP | Cardiometabolic |
| P06858 | Lipoprotein lipase | LPL | Cardiometabolic |
| Q8IZP9 | Adhesion G-protein coupled receptor G2 | ADGRG2 | Cardiometabolic |
| Q9Y286 | Sialic acid-binding Ig-like lectin 7 | SIGLEC7 | Cardiometabolic |
| O95502 | Neuronal pentraxin receptor | NPTXR | Cardiometabolic |
| O75791 | GRB2-related adapter protein 2 | GRAP2 | Cardiometabolic |
| Q9HBB8 | Cadherin-related family member 5 | CDHR5 | Cardiometabolic |
| P52888 | Thimet oligopeptidase | THOP1 | Cardiometabolic |
| P35754 | Glutaredoxin-1 | GLRX | Cardiometabolic |
| P21583 | Kit ligand | KITLG | Cardiometabolic |
| Q9UKJ0 | Paired immunoglobulin-like type 2 receptor beta | PILRB | Cardiometabolic |
| Q15846 | Clusterin-like protein 1 | CLUL1 | Cardiometabolic |
| P23526 | Adenosylhomocysteinase | AHCY | Cardiometabolic |
| P12104 | Fatty acid-binding protein, intestinal | FABP2 | Cardiometabolic |
| P00797 | Renin | REN | Cardiometabolic |
| Q9UK05 | Growth/differentiation factor 2 | GDF2 | Cardiometabolic |
| P12724 | Eosinophil cationic protein | RNASE3 | Cardiometabolic |
| Q9BQB4 | Sclerostin | SOST | Cardiometabolic |
| Q9NQX5 | Neural proliferation differentiation and control protein 1 | NPDC1 | Cardiometabolic |
| Q07108 | Early activation antigen CD69 | CD69 | Cardiometabolic |
| P42830 | C-X-C motif chemokine 5 | CXCL5 | Cardiometabolic |
| P02462 | Collagen alpha-1(IV) chain | COL4A1 | Cardiometabolic |
| A6NI73 | Leukocyte immunoglobulin-like receptor subfamily A member 5 | LILRA5 | Cardiometabolic |
| Q9UEW3 | Macrophage receptor MARCO | MARCO | Cardiometabolic |
| O95841 | Angiopoietin-related protein 1 | ANGPTL1 | Cardiometabolic |
| Q9BQR3 | Serine protease 27 | PRSS27 | Cardiometabolic |
| Q99523 | Sortilin | SORT1 | Cardiometabolic |
| P35247 | Pulmonary surfactant-associated protein D | SFTPD | Cardiometabolic |
| P20711 | Aromatic-L-amino-acid decarboxylase | DDC | Cardiometabolic |
| P31431 | Syndecan-4 | SDC4 | Cardiometabolic |
| P09601 | Heme oxygenase 1 | HMOX1 | Cardiometabolic |
| O00161 | Synaptosomal-associated protein 23 | SNAP23 | Cardiometabolic |
| Q8IW75 | Serpin A12 | SERPINA12 | Cardiometabolic |
| P01241 | Somatotropin | GH1 | Cardiometabolic |
| Q9BUD6 | Spondin-2 | SPON2 | Cardiometabolic |
| Q92692 | Nectin-2 | NECTIN2 | Cardiometabolic |
| Q6WN34 | Chordin-like protein 2 | CHRDL2 | Cardiometabolic |
| Q01973 | Inactive tyrosine-protein kinase transmembrane receptor ROR1 | ROR1 | Cardiometabolic |
| Q8N1Q1 | Carbonic anhydrase 13 | CA13 | Cardiometabolic |
| Q76M96 | Coiled-coil domain-containing protein 80 | CCDC80 | Cardiometabolic |
| P09467 | Fructose-1,6-bisphosphatase 1 | FBP1 | Cardiometabolic |
| P07711 | Cathepsin L1 | CTSL | Cardiometabolic |
| Q92823 | Neuronal cell adhesion molecule | NRCAM | Cardiometabolic |
| P04792 | Heat shock protein beta-1 | HSPB1 | Cardiometabolic |
| P07204 | Thrombomodulin | THBD | Cardiometabolic |
| Q16773 | Kynurenine--oxoglutarate transaminase 1 | KYAT1 | Cardiometabolic |
| Q92520 | Protein FAM3C | FAM3C | Cardiometabolic |
| P19971 | Thymidine phosphorylase | TYMP | Cardiometabolic |
| Q8NBP7 | Proprotein convertase subtilisin/kexin type 9 | PCSK9 | Cardiometabolic |
| Q16270 | Insulin-like growth factor-binding protein 7 | IGFBP7 | Cardiometabolic |
| P07911 | Uromodulin | UMOD | Cardiometabolic |
| P55058 | Phospholipid transfer protein | PLTP | Cardiometabolic |
| Q13361 | Microfibrillar-associated protein 5 | MFAP5 | Cardiometabolic |
| P01130 | Low-density lipoprotein receptor | LDLR | Cardiometabolic |
| P22748 | Carbonic anhydrase 4 | CA4 | Cardiometabolic |
| P54760 | Ephrin type-B receptor 4 | EPHB4 | Cardiometabolic |
| P33151 | Cadherin-5 | CDH5 | Cardiometabolic |
| P23141 | Liver carboxylesterase 1 | CES1 | Cardiometabolic |
| P15090 | Fatty acid-binding protein, adipocyte | FABP4 | Cardiometabolic |
| P08833 | Insulin-like growth factor-binding protein 1 | IGFBP1 | Cardiometabolic |
| P00750 | Tissue-type plasminogen activator | PLAT | Cardiometabolic |
| P13598 | Intercellular adhesion molecule 2 | ICAM2 | Cardiometabolic |
| Q76LX8 | A disintegrin and metalloproteinase with thrombospondin motifs 13 | ADAMTS13 | Cardiometabolic |
| Q01638 | Interleukin-1 receptor-like 1 | IL1RL1 | Cardiometabolic |
| Q99988 | Growth/differentiation factor 15 | GDF15 | Cardiometabolic |
| P04054 | Phospholipase A2 | PLA2G1B | Cardiometabolic |
| Q9UM47 | Neurogenic locus notch homolog protein 3 | NOTCH3 | Cardiometabolic |
| Q14767 | Latent-transforming growth factor beta-binding protein 2 | LTBP2 | Cardiometabolic |
| Q03167 | Transforming growth factor beta receptor type 3 | TGFBR3 | Cardiometabolic |
| P04275 | von Willebrand factor | VWF | Cardiometabolic |
| Q5VY43 | Platelet endothelial aggregation receptor 1 | PEAR1 | Cardiometabolic |
| Q06141 | Regenerating islet-derived protein 3-alpha | REG3A | Cardiometabolic |
| P55808 | Glycoprotein Xg | XG | Cardiometabolic |
| P48960 | Adhesion G protein-coupled receptor E5 | ADGRE5 | Cardiometabolic |
| P04066 | Tissue alpha-L-fucosidase | FUCA1 | Cardiometabolic |
| Q9NNX6 | CD209 antigen | CD209 | Cardiometabolic |
| P02144 | Myoglobin | MB | Cardiometabolic |
| Q9UBP4 | Dickkopf-related protein 3 | DKK3 | Cardiometabolic |
| O14798 | Tumor necrosis factor receptor superfamily member 10C | TNFRSF10C | Cardiometabolic |
| P48304 | Lithostathine-1-beta | REG1B | Cardiometabolic |
| P01589 | Interleukin-2 receptor subunit alpha | IL2RA | Cardiometabolic |
| P09619 | Platelet-derived growth factor receptor beta | PDGFRB | Cardiometabolic |
| P08581 | Hepatocyte growth factor receptor | MET | Cardiometabolic |
| Q96AP7 | Endothelial cell-selective adhesion molecule | ESAM | Cardiometabolic |
| P07451 | Carbonic anhydrase 3 | CA3 | Cardiometabolic |
| Q9Y275 | Tumor necrosis factor ligand superfamily member 13B | TNFSF13B | Cardiometabolic |
| Q13740 | CD166 antigen | ALCAM | Cardiometabolic |
| P14555 | Phospholipase A2, membrane associated | PLA2G2A | Cardiometabolic |
| P08118 | Beta-microseminoprotein | MSMB | Cardiometabolic |
| P07858 | Cathepsin B | CTSB | Cardiometabolic |
| P32942 | Intercellular adhesion molecule 3 | ICAM3 | Cardiometabolic |
| Q07507 | Dermatopontin | DPT | Cardiometabolic |
| Q9NQ79 | Cartilage acidic protein 1 | CRTAC1 | Cardiometabolic |
| P15085 | Carboxypeptidase A1 | CPA1 | Cardiometabolic |
| Q86U17 | Serpin A11 | SERPINA11 | Cardiometabolic |
| Q9H2A7 | C-X-C motif chemokine 16 | CXCL16 | Cardiometabolic |
| O75326 | Semaphorin-7A | SEMA7A | Cardiometabolic |
| P17931 | Galectin-3 | LGALS3 | Cardiometabolic |
| Q9H1U4 | Multiple epidermal growth factor-like domains protein 9 | MEGF9 | Cardiometabolic |
| P31146 | Coronin-1A | CORO1A | Cardiometabolic |
| P17813 | Endoglin | ENG | Cardiometabolic |
| Q13822 | Ectonucleotide pyrophosphatase/phosphodiesterase family member 2 | ENPP2 | Cardiometabolic |
| P15086 | Carboxypeptidase B | CPB1 | Cardiometabolic |
| P16581 | E-selectin | SELE | Cardiometabolic |
| P19021 | Peptidyl-glycine alpha-amidating monooxygenase | PAM | Cardiometabolic |
| P12111 | Collagen alpha-3(VI) chain | COL6A3 | Cardiometabolic |
| Q15828 | Cystatin-M | CST6 | Cardiometabolic |
| P04080 | Cystatin-B | CSTB | Cardiometabolic |
| P80370 | Protein delta homolog 1 | DLK1 | Cardiometabolic |
| P08236 | Beta-glucuronidase | GUSB | Cardiometabolic |
| Q14162 | Scavenger receptor class F member 1 | SCARF1 | Cardiometabolic |
| P09093 | Chymotrypsin-like elastase family member 3A | CELA3A | Cardiometabolic |
| P48745 | CCN family member 3 | CCN3 | Cardiometabolic |
| Q9Y4L1 | Hypoxia up-regulated protein 1 | HYOU1 | Cardiometabolic |
| Q8N423 | Leukocyte immunoglobulin-like receptor subfamily B member 2 | LILRB2 | Cardiometabolic |
| P15907 | Beta-galactoside alpha-2,6-sialyltransferase 1 | ST6GAL1 | Cardiometabolic |
| P35590 | Tyrosine-protein kinase receptor Tie-1 | TIE1 | Cardiometabolic |
| P78324 | Tyrosine-protein phosphatase non-receptor type substrate 1 | SIRPA | Cardiometabolic |
| P42574 | Caspase-3 | CASP3 | Cardiometabolic |
| Q13332 | Receptor-type tyrosine-protein phosphatase S | PTPRS | Cardiometabolic |
| Q12860 | Contactin-1 | CNTN1 | Cardiometabolic |
| Q8TDL5 | BPI fold-containing family B member 1 | BPIFB1 | Cardiometabolic |
| P20160 | Azurocidin | AZU1 | Cardiometabolic |
| P10586 | Receptor-type tyrosine-protein phosphatase F | PTPRF | Cardiometabolic |
| P46531 | Neurogenic locus notch homolog protein 1 | NOTCH1 | Cardiometabolic |
| Q13231 | Chitotriosidase-1 | CHIT1 | Cardiometabolic |
| P04085 | Platelet-derived growth factor subunit A | PDGFA | Cardiometabolic |
| P13686 | Tartrate-resistant acid phosphatase type 5 | ACP5 | Cardiometabolic |
| P05107 | Integrin beta-2 | ITGB2 | Cardiometabolic |
| P25445 | Tumor necrosis factor receptor superfamily member 6 | FAS | Cardiometabolic |
| O15031 | Plexin-B2 | PLXNB2 | Cardiometabolic |
| Q14393 | Growth arrest-specific protein 6 | GAS6 | Cardiometabolic |
| P00533 | Epidermal growth factor receptor | EGFR | Cardiometabolic |
| Q16769 | Glutaminyl-peptide cyclotransferase | QPCT | Cardiometabolic |
| O00584 | Ribonuclease T2 | RNASET2 | Cardiometabolic |
| P10451 | Osteopontin | SPP1 | Cardiometabolic |
| Q8NHL6 | Leukocyte immunoglobulin-like receptor subfamily B member 1 | LILRB1 | Cardiometabolic |
| O75023 | Leukocyte immunoglobulin-like receptor subfamily B member 5 | LILRB5 | Cardiometabolic |
| P18065 | Insulin-like growth factor-binding protein 2 | IGFBP2 | Cardiometabolic |
| P19957 | Elafin | PI3 | Cardiometabolic |
| Q9HD89 | Resistin | RETN | Cardiometabolic |
| Q16663 | C-C motif chemokine 15 | CCL15 | Cardiometabolic |
| P24158 | Myeloblastin | PRTN3 | Cardiometabolic |
| Q12884 | Prolyl endopeptidase FAP | FAP | Cardiometabolic |
| P23284 | Peptidyl-prolyl cis-trans isomerase B | PPIB | Cardiometabolic |
| P39060 | Collagen alpha-1(XVIII) chain | COL18A1 | Cardiometabolic |
| P04746 | Pancreatic alpha-amylase | AMY2A | Cardiometabolic |
| O15467 | C-C motif chemokine 16 | CCL16 | Cardiometabolic |
| P02452 | Collagen alpha-1(I) chain | COL1A1 | Cardiometabolic |
| Q13867 | Bleomycin hydrolase | BLMH | Cardiometabolic |
| P42785 | Lysosomal Pro-X carboxypeptidase | PRCP | Cardiometabolic |
| O75594 | Peptidoglycan recognition protein 1 | PGLYRP1 | Cardiometabolic |
| P13987 | CD59 glycoprotein | CD59 | Cardiometabolic |
| P19961 | Alpha-amylase 2B | AMY2B | Cardiometabolic |
| P20062 | Transcobalamin-2 | TCN2 | Cardiometabolic |
| P05121 | Plasminogen activator inhibitor 1 | SERPINE1 | Cardiometabolic |
| P43121 | Cell surface glycoprotein MUC18 | MCAM | Cardiometabolic |
| P59665 | Neutrophil defensin 1 | DEFA1_DEFA1B | Cardiometabolic |
| Q6EMK4 | Vasorin | VASN | Cardiometabolic |
| Q13508 | Ecto-ADP-ribosyltransferase 3 | ART3 | Cardiometabolic |
| Q96KN2 | Beta-Ala-His dipeptidase | CNDP1 | Cardiometabolic |
| O95998 | Interleukin-18-binding protein | IL18BP | Cardiometabolic |
| P00740 | Coagulation factor IX | F9 | Cardiometabolic |
| P15144 | Aminopeptidase N | ANPEP | Cardiometabolic |
| A1L4H1 | Soluble scavenger receptor cysteine-rich domain-containing protein SSC5D | SSC5D | Cardiometabolic |
| Q06033 | Inter-alpha-trypsin inhibitor heavy chain H3 | ITIH3 | Cardiometabolic |
| P12830 | Cadherin-1 | CDH1 | Cardiometabolic |
| P13591 | Neural cell adhesion molecule 1 | NCAM1 | Cardiometabolic |
| P18428 | Lipopolysaccharide-binding protein | LBP | Cardiometabolic |
| Q99650 | Oncostatin-M-specific receptor subunit beta | OSMR | Cardiometabolic |
| Q12794 | Hyaluronidase-1 | HYAL1 | Cardiometabolic |
| P07339 | Cathepsin D | CTSD | Cardiometabolic |
| P24821 | Tenascin | TNC | Cardiometabolic |
| Q86VB7 | Scavenger receptor cysteine-rich type 1 protein M130 | CD163 | Cardiometabolic |
| Q14515 | SPARC-like protein 1 | SPARCL1 | Cardiometabolic |
| P14543 | Nidogen-1 | NID1 | Cardiometabolic |
| P30530 | Tyrosine-protein kinase receptor UFO | AXL | Cardiometabolic |
| P07478 | Trypsin-2 | PRSS2 | Cardiometabolic |
| Q9UBR2 | Cathepsin Z | CTSZ | Cardiometabolic |
| O00533 | Neural cell adhesion molecule L1-like protein | CHL1 | Cardiometabolic |
| Q9BXJ1 | Complement C1q tumor necrosis factor-related protein 1 | C1QTNF1 | Cardiometabolic |
| P35443 | Thrombospondin-4 | THBS4 | Cardiometabolic |
| P02786 | Transferrin receptor protein 1 | TFRC | Cardiometabolic |
| P10721 | Mast/stem cell growth factor receptor Kit | KIT | Cardiometabolic |
| Q07654 | Trefoil factor 3 | TFF3 | Cardiometabolic |
| P08709 | Coagulation factor VII | F7 | Cardiometabolic |
| Q99969 | Retinoic acid receptor responder protein 2 | RARRES2 | Cardiometabolic |
| O95445 | Apolipoprotein M | APOM | Cardiometabolic |
| Q96H15 | T-cell immunoglobulin and mucin domain-containing protein 4 | TIMD4 | Cardiometabolic |
| P05556 | Integrin beta-1 | ITGB1 | Cardiometabolic |
| P08174 | Complement decay-accelerating factor | CD55 | Cardiometabolic |
| P08571 | Monocyte differentiation antigen CD14 | CD14 | Cardiometabolic |
| P16109 | P-selectin | SELP | Cardiometabolic |
| P15529 | Membrane cofactor protein | CD46 | Cardiometabolic |
| P07359 | Platelet glycoprotein Ib alpha chain | GP1BA | Cardiometabolic |
| Q15485 | Ficolin-2 | FCN2 | Cardiometabolic |
| P98160 | Basement membrane-specific heparan sulfate proteoglycan core protein | HSPG2 | Cardiometabolic |
| Q15113 | Procollagen C-endopeptidase enhancer 1 | PCOLCE | Cardiometabolic |
| P08887 | Interleukin-6 receptor subunit alpha | IL6R | Cardiometabolic |
| P00441 | Superoxide dismutase [Cu-Zn] | SOD1 | Cardiometabolic |
| O75015 | Low affinity immunoglobulin gamma Fc region receptor III-B | FCGR3B | Cardiometabolic |
| P10646 | Tissue factor pathway inhibitor | TFPI | Cardiometabolic |
| Q16853 | Membrane primary amine oxidase | AOC3 | Cardiometabolic |
| O14786 | Neuropilin-1 | NRP1 | Cardiometabolic |
| P12318 | Low affinity immunoglobulin gamma Fc region receptor II-a | FCGR2A | Cardiometabolic |
| P80188 | Neutrophil gelatinase-associated lipocalin | LCN2 | Cardiometabolic |
| P20023 | Complement receptor type 2 | CR2 | Cardiometabolic |
| Q9NZK5 | Adenosine deaminase 2 | ADA2 | Cardiometabolic |
| P55774 | C-C motif chemokine 18 | CCL18 | Cardiometabolic |
| P19320 | Vascular cell adhesion protein 1 | VCAM1 | Cardiometabolic |
| Q9NPY3 | Complement component C1q receptor | CD93 | Cardiometabolic |
| P17936 | Insulin-like growth factor-binding protein 3 | IGFBP3 | Cardiometabolic |
| P36222 | Chitinase-3-like protein 1 | CHI3L1 | Cardiometabolic |
| P01034 | Cystatin-C | CST3 | Cardiometabolic |
| Q16627 | C-C motif chemokine 14 | CCL14 | Cardiometabolic |
| P04070 | Vitamin K-dependent protein C | PROC | Cardiometabolic |
| P03950 | Angiogenin | ANG | Cardiometabolic |
| Q9UGM5 | Fetuin-B | FETUB | Cardiometabolic |
| P49747 | Cartilage oligomeric matrix protein | COMP | Cardiometabolic |
| P27487 | Dipeptidyl peptidase 4 | DPP4 | Cardiometabolic |
| Q9Y5C1 | Angiopoietin-related protein 3 | ANGPTL3 | Cardiometabolic |
| P41222 | Prostaglandin-H2 D-isomerase | PTGDS | Cardiometabolic |
| P00915 | Carbonic anhydrase 1 | CA1 | Cardiometabolic |
| P06681 | Complement C2 | C2 | Cardiometabolic |
| P05362 | Intercellular adhesion molecule 1 | ICAM1 | Cardiometabolic |
| P13501 | C-C motif chemokine 5 | CCL5 | Cardiometabolic |
| P24592 | Insulin-like growth factor-binding protein 6 | IGFBP6 | Cardiometabolic |
| Q12805 | EGF-containing fibulin-like extracellular matrix protein 1 | EFEMP1 | Cardiometabolic |
| P05451 | Lithostathine-1-alpha | REG1A | Cardiometabolic |
| Q92820 | Gamma-glutamyl hydrolase | GGH | Cardiometabolic |
| Q15582 | Transforming growth factor-beta-induced protein ig-h3 | TGFBI | Cardiometabolic |
| P01033 | Metalloproteinase inhibitor 1 | TIMP1 | Cardiometabolic |
| Q8IZC4 | Rhotekin-2 | RTKN2 | Cardiometabolic_II |
| P78524 | DENN domain-containing protein 2B | DENND2B | Cardiometabolic_II |
| Q9H2M3 | S-methylmethionine--homocysteine S-methyltransferase BHMT2 | BHMT2 | Cardiometabolic_II |
| P55769 | NHP2-like protein 1 | SNU13 | Cardiometabolic_II |
| Q9Y2W1 | Thyroid hormone receptor-associated protein 3 | THRAP3 | Cardiometabolic_II |
| O43734 | E3 ubiquitin ligase TRAF3IP2 | TRAF3IP2 | Cardiometabolic_II |
| O00567 | Nucleolar protein 56 | NOP56 | Cardiometabolic_II |
| Q15477 | Helicase SKI2W | SKIV2L | Cardiometabolic_II |
| P25391 | Laminin subunit alpha-1 | LAMA1 | Cardiometabolic_II |
| P06753 | Tropomyosin alpha-3 chain | TPM3 | Cardiometabolic_II |
| P48507 | Glutamate--cysteine ligase regulatory subunit | GCLM | Cardiometabolic_II |
| Q9NZJ5 | Eukaryotic translation initiation factor 2-alpha kinase 3 | EIF2AK3 | Cardiometabolic_II |
| Q9Y623 | Myosin-4 | MYH4 | Cardiometabolic_II |
| P23634 | Plasma membrane calcium-transporting ATPase 4 | ATP2B4 | Cardiometabolic_II |
| O14958 | Calsequestrin-2 | CASQ2 | Cardiometabolic_II |
| O95180 | Voltage-dependent T-type calcium channel subunit alpha-1H | CACNA1H | Cardiometabolic_II |
| P54709 | Sodium/potassium-transporting ATPase subunit beta-3 | ATP1B3 | Cardiometabolic_II |
| Q13503 | Mediator of RNA polymerase II transcription subunit 21 | MED21 | Cardiometabolic_II |
| P08913 | Alpha-2A adrenergic receptor | ADRA2A | Cardiometabolic_II |
| P49755 | Transmembrane emp24 domain-containing protein 10 | TMED10 | Cardiometabolic_II |
| Q96DA2 | Ras-related protein Rab-39B | RAB39B | Cardiometabolic_II |
| P46783 | 40S ribosomal protein S10 | RPS10 | Cardiometabolic_II |
| O00291 | Huntingtin-interacting protein 1 | HIP1 | Cardiometabolic_II |
| P04141 | Granulocyte-macrophage colony-stimulating factor | CSF2 | Cardiometabolic_II |
| A6NCE7 | Microtubule-associated proteins 1A/1B light chain 3 beta 2 | MAP1LC3B2 | Cardiometabolic_II |
| Q9Y3B8 | Oligoribonuclease, mitochondrial | REXO2 | Cardiometabolic_II |
| Q6UWF7 | NXPE family member 4 | NXPE4 | Cardiometabolic_II |
| P55011 | Solute carrier family 12 member 2 | SLC12A2 | Cardiometabolic_II |
| P10109 | Adrenodoxin, mitochondrial | FDX1 | Cardiometabolic_II |
| P30049 | ATP synthase subunit delta, mitochondrial | ATP5F1D | Cardiometabolic_II |
| P33121 | Long-chain-fatty-acid--CoA ligase 1 | ACSL1 | Cardiometabolic_II |
| O60701 | UDP-glucose 6-dehydrogenase | UGDH | Cardiometabolic_II |
| Q9BY32 | Inosine triphosphate pyrophosphatase | ITPA | Cardiometabolic_II |
| Q01780 | Exosome component 10 | EXOSC10 | Cardiometabolic_II |
| Q08499 | cAMP-specific 3',5'-cyclic phosphodiesterase 4D | PDE4D | Cardiometabolic_II |
| P21817 | Ryanodine receptor 1 | RYR1 | Cardiometabolic_II |
| Q96HD9 | N-acyl-aromatic-L-amino acid amidohydrolase | ACY3 | Cardiometabolic_II |
| P35228 | Nitric oxide synthase, inducible | NOS2 | Cardiometabolic_II |
| E2RYF7 | Protein PBMUCL2 | HCG22 | Cardiometabolic_II |
| Q9NVZ3 | Adaptin ear-binding coat-associated protein 2 | NECAP2 | Cardiometabolic_II |
| Q9Y4C8 | Probable RNA-binding protein 19 | RBM19 | Cardiometabolic_II |
| Q07973 | 1,25-dihydroxyvitamin D(3) 24-hydroxylase, mitochondrial | CYP24A1 | Cardiometabolic_II |
| Q04695 | Keratin, type I cytoskeletal 17 | KRT17 | Cardiometabolic_II |
| Q15059 | Bromodomain-containing protein 3 | BRD3 | Cardiometabolic_II |
| B6SEH8 | Endogenous retrovirus group V member 1 Env polyprotein | ERVV-1 | Cardiometabolic_II |
| O95858 | Tetraspanin-15 | TSPAN15 | Cardiometabolic_II |
| Q9H347 | Ubiquilin-3 | UBQLN3 | Cardiometabolic_II |
| P06729 | T-cell surface antigen CD2 | CD2 | Cardiometabolic_II |
| Q96IW2 | SH2 domain-containing adapter protein D | SHD | Cardiometabolic_II |
| A6BM72 | Multiple epidermal growth factor-like domains protein 11 | MEGF11 | Cardiometabolic_II |
| Q9UKX7 | Nuclear pore complex protein Nup50 | NUP50 | Cardiometabolic_II |
| Q96LB8 | Peptidoglycan recognition protein 4 | PGLYRP4 | Cardiometabolic_II |
| Q9NV35 | Nucleotide triphosphate diphosphatase NUDT15 | NUDT15 | Cardiometabolic_II |
| Q10587 | Thyrotroph embryonic factor | TEF | Cardiometabolic_II |
| Q13296 | Mammaglobin-A | SCGB2A2 | Cardiometabolic_II |
| P20929 | Nebulin | NEB | Cardiometabolic_II |
| Q5TA50 | Ceramide-1-phosphate transfer protein | CPTP | Cardiometabolic_II |
| Q86UW2 | Organic solute transporter subunit beta | SLC51B | Cardiometabolic_II |
| Q8WZ42 | Titin | TTN | Cardiometabolic_II |
| Q9UFP1 | Golgi-associated kinase 1A | GASK1A | Cardiometabolic_II |
| Q99707 | Methionine synthase | MTR | Cardiometabolic_II |
| P21673 | Diamine acetyltransferase 1 | SAT1 | Cardiometabolic_II |
| O00425 | Insulin-like growth factor 2 mRNA-binding protein 3 | IGF2BP3 | Cardiometabolic_II |
| O43290 | U4/U6.U5 tri-snRNP-associated protein 1 | SART1 | Cardiometabolic_II |
| Q92935 | Exostosin-like 1 | EXTL1 | Cardiometabolic_II |
| Q8N8E3 | Centrosomal protein of 112 kDa | CEP112 | Cardiometabolic_II |
| P16066 | Atrial natriuretic peptide receptor 1 | NPR1 | Cardiometabolic_II |
| Q6NZY4 | Zinc finger CCHC domain-containing protein 8 | ZCCHC8 | Cardiometabolic_II |
| P14415 | Sodium/potassium-transporting ATPase subunit beta-2 | ATP1B2 | Cardiometabolic_II |
| Q96K76 | Ubiquitin carboxyl-terminal hydrolase 47 | USP47 | Cardiometabolic_II |
| P05976 | Myosin light chain 1/3, skeletal muscle isoform | MYL1 | Cardiometabolic_II |
| Q9Y2Y0 | ADP-ribosylation factor-like protein 2-binding protein | ARL2BP | Cardiometabolic_II |
| Q14088 | Ras-related protein Rab-33A | RAB33A | Cardiometabolic_II |
| P38935 | DNA-binding protein SMUBP-2 | IGHMBP2 | Cardiometabolic_II |
| P05026 | Sodium/potassium-transporting ATPase subunit beta-1 | ATP1B1 | Cardiometabolic_II |
| O15305 | Phosphomannomutase 2 | PMM2 | Cardiometabolic_II |
| Q9BW61 | DET1- and DDB1-associated protein 1 | DDA1 | Cardiometabolic_II |
| Q15370 | Elongin-B | ELOB | Cardiometabolic_II |
| Q8NET8 | Transient receptor potential cation channel subfamily V member 3 | TRPV3 | Cardiometabolic_II |
| P05000 | Interferon omega-1 | IFNW1 | Cardiometabolic_II |
| Q15018 | BRISC complex subunit Abraxas 2 | ABRAXAS2 | Cardiometabolic_II |
| P54296 | Myomesin-2 | MYOM2 | Cardiometabolic_II |
| Q16836 | Hydroxyacyl-coenzyme A dehydrogenase, mitochondrial | HADH | Cardiometabolic_II |
| Q14353 | Guanidinoacetate N-methyltransferase | GAMT | Cardiometabolic_II |
| P23511 | Nuclear transcription factor Y subunit alpha | NFYA | Cardiometabolic_II |
| Q07075 | Glutamyl aminopeptidase | ENPEP | Cardiometabolic_II |
| Q9BV94 | ER degradation-enhancing alpha-mannosidase-like protein 2 | EDEM2 | Cardiometabolic_II |
| Q02127 | Dihydroorotate dehydrogenase | DHODH | Cardiometabolic_II |
| P57078 | Receptor-interacting serine/threonine-protein kinase 4 | RIPK4 | Cardiometabolic_II |
| Q6ZN66 | Guanylate-binding protein 6 | GBP6 | Cardiometabolic_II |
| Q9BZL6 | Serine/threonine-protein kinase D2 | PRKD2 | Cardiometabolic_II |
| A6NHS7 | MANSC domain-containing protein 4 | MANSC4 | Cardiometabolic_II |
| O75521 | Enoyl-CoA delta isomerase 2 | ECI2 | Cardiometabolic_II |
| P12270 | Nucleoprotein TPR | TPR | Cardiometabolic_II |
| Q9NYX4 | Neuron-specific vesicular protein calcyon | CALY | Cardiometabolic_II |
| P37058 | Testosterone 17-beta-dehydrogenase 3 | HSD17B3 | Cardiometabolic_II |
| Q9BZC7 | ATP-binding cassette sub-family A member 2 | ABCA2 | Cardiometabolic_II |
| Q6P4F2 | Ferredoxin-2, mitochondrial | FDX2 | Cardiometabolic_II |
| Q16774 | Guanylate kinase | GUK1 | Cardiometabolic_II |
| Q9UNN8 | Endothelial protein C receptor | PROCR | Cardiometabolic_II |
| P10082 | Peptide YY | PYY | Cardiometabolic_II |
| O15018 | PDZ domain-containing protein 2 | PDZD2 | Cardiometabolic_II |
| Q16206 | Ecto-NOX disulfide-thiol exchanger 2 | ENOX2 | Cardiometabolic_II |
| P0C7L1 | Serine protease inhibitor Kazal-type 8 | SPINK8 | Cardiometabolic_II |
| Q7Z7H5 | Transmembrane emp24 domain-containing protein 4 | TMED4 | Cardiometabolic_II |
| Q9Y2L6 | FERM domain-containing protein 4B | FRMD4B | Cardiometabolic_II |
| P55010 | Eukaryotic translation initiation factor 5 | EIF5 | Cardiometabolic_II |
| Q01581 | Hydroxymethylglutaryl-CoA synthase, cytoplasmic | HMGCS1 | Cardiometabolic_II |
| Q12986 | Transcriptional repressor NF-X1 | NFX1 | Cardiometabolic_II |
| P22033 | Methylmalonyl-CoA mutase, mitochondrial | MMUT | Cardiometabolic_II |
| P19838 | Nuclear factor NF-kappa-B p105 subunit | NFKB1 | Cardiometabolic_II |
| Q01484 | Ankyrin-2 | ANK2 | Cardiometabolic_II |
| P59901 | Leukocyte immunoglobulin-like receptor subfamily A member 4 | LILRA4 | Cardiometabolic_II |
| O43896 | Kinesin-like protein KIF1C | KIF1C | Cardiometabolic_II |
| Q03013 | Glutathione S-transferase Mu 4 | GSTM4 | Cardiometabolic_II |
| O94766 | Galactosylgalactosylxylosylprotein 3-beta-glucuronosyltransferase 3 | B3GAT3 | Cardiometabolic_II |
| Q3SXY8 | ADP-ribosylation factor-like protein 13B | ARL13B | Cardiometabolic_II |
| O95670 | V-type proton ATPase subunit G 2 | ATP6V1G2 | Cardiometabolic_II |
| O00327 | Aryl hydrocarbon receptor nuclear translocator-like protein 1 | ARNTL | Cardiometabolic_II |
| P48668 | Keratin, type II cytoskeletal 6C | KRT6C | Cardiometabolic_II |
| P00966 | Argininosuccinate synthase | ASS1 | Cardiometabolic_II |
| Q96PU4 | E3 ubiquitin-protein ligase UHRF2 | UHRF2 | Cardiometabolic_II |
| P20382 | Pro-MCH | PMCH | Cardiometabolic_II |
| P35606 | Coatomer subunit beta' | COPB2 | Cardiometabolic_II |
| P13224 | Platelet glycoprotein Ib beta chain | GP1BB | Cardiometabolic_II |
| Q14807 | Kinesin-like protein KIF22 | KIF22 | Cardiometabolic_II |
| P50461 | Cysteine and glycine-rich protein 3 | CSRP3 | Cardiometabolic_II |
| Q14781 | Chromobox protein homolog 2 | CBX2 | Cardiometabolic_II |
| Q96A35 | 39S ribosomal protein L24, mitochondrial | MRPL24 | Cardiometabolic_II |
| Q58F21 | Bromodomain testis-specific protein | BRDT | Cardiometabolic_II |
| Q96EU7 | C1GALT1-specific chaperone 1 | C1GALT1C1 | Cardiometabolic_II |
| Q5VVQ6 | Ubiquitin thioesterase OTU1 | YOD1 | Cardiometabolic_II |
| A6NDB9 | Paralemmin-3 | PALM3 | Cardiometabolic_II |
| O75534 | Cold shock domain-containing protein E1 | CSDE1 | Cardiometabolic_II |
| Q13563 | Polycystin-2 | PKD2 | Cardiometabolic_II |
| Q99598 | Translin-associated protein X | TSNAX | Cardiometabolic_II |
| Q86VP3 | Phosphofurin acidic cluster sorting protein 2 | PACS2 | Cardiometabolic_II |
| Q5W0V3 | FHF complex subunit HOOK interacting protein 2A | FHIP2A | Cardiometabolic_II |
| P41227 | N-alpha-acetyltransferase 10 | NAA10 | Cardiometabolic_II |
| Q86VR7 | V-set and immunoglobulin domain-containing protein 10-like | VSIG10L | Cardiometabolic_II |
| Q93052 | Lipoma-preferred partner | LPP | Cardiometabolic_II |
| O75427 | Leucine-rich repeat and calponin homology domain-containing protein 4 | LRCH4 | Cardiometabolic_II |
| P35609 | Alpha-actinin-2 | ACTN2 | Cardiometabolic_II |
| P32241 | Vasoactive intestinal polypeptide receptor 1 | VIPR1 | Cardiometabolic_II |
| Q8ND90 | Paraneoplastic antigen Ma1 | PNMA1 | Cardiometabolic_II |
| Q5JTV8 | Torsin-1A-interacting protein 1 | TOR1AIP1 | Cardiometabolic_II |
| Q9UBV2 | Protein sel-1 homolog 1 | SEL1L | Cardiometabolic_II |
| P46926 | Glucosamine-6-phosphate isomerase 1 | GNPDA1 | Cardiometabolic_II |
| Q8NFP7 | Diphosphoinositol polyphosphate phosphohydrolase 3-alpha | NUDT10 | Cardiometabolic_II |
| Q14324 | Myosin-binding protein C, fast-type | MYBPC2 | Cardiometabolic_II |
| P35520 | Cystathionine beta-synthase | CBS | Cardiometabolic_II |
| O14841 | 5-oxoprolinase | OPLAH | Cardiometabolic_II |
| Q8WXC3 | Pyrin domain-containing protein 1 | PYDC1 | Cardiometabolic_II |
| O43423 | Acidic leucine-rich nuclear phosphoprotein 32 family member C | ANP32C | Cardiometabolic_II |
| Q9BQI0 | Allograft inflammatory factor 1-like | AIF1L | Cardiometabolic_II |
| Q8TER0 | Sushi, nidogen and EGF-like domain-containing protein 1 | SNED1 | Cardiometabolic_II |
| Q9BTK6 | PAXIP1-associated glutamate-rich protein 1 | PAGR1 | Cardiometabolic_II |
| Q9H173 | Nucleotide exchange factor SIL1 | SIL1 | Cardiometabolic_II |
| P20645 | Cation-dependent mannose-6-phosphate receptor | M6PR | Cardiometabolic_II |
| P13929 | Beta-enolase | ENO3 | Cardiometabolic_II |
| Q96ID5 | Immunoglobulin superfamily member 21 | IGSF21 | Cardiometabolic_II |
| P23327 | Sarcoplasmic reticulum histidine-rich calcium-binding protein | HRC | Cardiometabolic_II |
| P29536 | Leiomodin-1 | LMOD1 | Cardiometabolic_II |
| Q13316 | Dentin matrix acidic phosphoprotein 1 | DMP1 | Cardiometabolic_II |
| P35914 | Hydroxymethylglutaryl-CoA lyase, mitochondrial | HMGCL | Cardiometabolic_II |
| Q9Y5X3 | Sorting nexin-5 | SNX5 | Cardiometabolic_II |
| Q14643 | Inositol 1,4,5-trisphosphate receptor type 1 | ITPR1 | Cardiometabolic_II |
| Q99807 | 5-demethoxyubiquinone hydroxylase, mitochondrial | COQ7 | Cardiometabolic_II |
| Q99942 | E3 ubiquitin-protein ligase RNF5 | RNF5 | Cardiometabolic_II |
| P36776 | Lon protease homolog, mitochondrial | LONP1 | Cardiometabolic_II |
| Q14457 | Beclin-1 | BECN1 | Cardiometabolic_II |
| I3L3R5 | Coiled-coil domain-containing glutamate-rich protein 2 | CCER2 | Cardiometabolic_II |
| Q8N668 | COMM domain-containing protein 1 | COMMD1 | Cardiometabolic_II |
| P11532 | Dystrophin | DMD | Cardiometabolic_II |
| P05305 | Endothelin-1 | EDN1 | Cardiometabolic_II |
| Q14160 | Protein scribble homolog | SCRIB | Cardiometabolic_II |
| Q8WZ75 | Roundabout homolog 4 | ROBO4 | Cardiometabolic_II |
| P55809 | Succinyl-CoA:3-ketoacid coenzyme A transferase 1, mitochondrial | OXCT1 | Cardiometabolic_II |
| Q9BY49 | Peroxisomal trans-2-enoyl-CoA reductase | PECR | Cardiometabolic_II |
| Q9NZN3 | EH domain-containing protein 3 | EHD3 | Cardiometabolic_II |
| P14902 | Indoleamine 2,3-dioxygenase 1 | IDO1 | Cardiometabolic_II |
| Q96C92 | Endosome-associated-trafficking regulator 1 | ENTR1 | Cardiometabolic_II |
| O75506 | Heat shock factor-binding protein 1 | HSBP1 | Cardiometabolic_II |
| P01225 | Follitropin subunit beta | FSHB | Cardiometabolic_II |
| O95980 | Reversion-inducing cysteine-rich protein with Kazal motifs | RECK | Cardiometabolic_II |
| Q8NC42 | E3 ubiquitin-protein ligase RNF149 | RNF149 | Cardiometabolic_II |
| Q9H7C9 | Mth938 domain-containing protein | AAMDC | Cardiometabolic_II |
| Q8TAE8 | Growth arrest and DNA damage-inducible proteins-interacting protein 1 | GADD45GIP1 | Cardiometabolic_II |
| Q5GAN6 | Inactive ribonuclease-like protein 10 | RNASE10 | Cardiometabolic_II |
| P30084 | Enoyl-CoA hydratase, mitochondrial | ECHS1 | Cardiometabolic_II |
| Q5SW79 | Centrosomal protein of 170 kDa | CEP170 | Cardiometabolic_II |
| P50053 | Ketohexokinase | KHK | Cardiometabolic_II |
| O75348 | V-type proton ATPase subunit G 1 | ATP6V1G1 | Cardiometabolic_II |
| Q53T59 | HCLS1-binding protein 3 | HS1BP3 | Cardiometabolic_II |
| Q8IVF2 | Protein AHNAK2 | AHNAK2 | Cardiometabolic_II |
| P12829 | Myosin light chain 4 | MYL4 | Cardiometabolic_II |
| Q0VD83 | Apolipoprotein B receptor | APOBR | Cardiometabolic_II |
| O75061 | Putative tyrosine-protein phosphatase auxilin | DNAJC6 | Cardiometabolic_II |
| Q8WUF8 | Cotranscriptional regulator FAM172A | FAM172A | Cardiometabolic_II |
| Q13137 | Calcium-binding and coiled-coil domain-containing protein 2 | CALCOCO2 | Cardiometabolic_II |
| P58107 | Epiplakin | EPPK1 | Cardiometabolic_II |
| P08590 | Myosin light chain 3 | MYL3 | Cardiometabolic_II |
| Q86X76 | Deaminated glutathione amidase | NIT1 | Cardiometabolic_II |
| P0DPI2 | Glutamine amidotransferase-like class 1 domain-containing protein 3B, mitochondrial | GATD3 | Cardiometabolic_II |
| Q12841 | Follistatin-related protein 1 | FSTL1 | Cardiometabolic_II |
| Q9NR61 | Delta-like protein 4 | DLL4 | Cardiometabolic_II |
| Q6YN16 | Hydroxysteroid dehydrogenase-like protein 2 | HSDL2 | Cardiometabolic_II |
| Q6ZRY4 | RNA-binding protein with multiple splicing 2 | RBPMS2 | Cardiometabolic_II |
| P43487 | Ran-specific GTPase-activating protein | RANBP1 | Cardiometabolic_II |
| Q92835 | Phosphatidylinositol 3,4,5-trisphosphate 5-phosphatase 1 | INPP5D | Cardiometabolic_II |
| Q09666 | Neuroblast differentiation-associated protein AHNAK | AHNAK | Cardiometabolic_II |
| Q9NYZ4 | Sialic acid-binding Ig-like lectin 8 | SIGLEC8 | Cardiometabolic_II |
| O60476 | Mannosyl-oligosaccharide 1,2-alpha-mannosidase IB | MAN1A2 | Cardiometabolic_II |
| P07355 | Annexin A2 | ANXA2 | Cardiometabolic_II |
| P07492 | Gastrin-releasing peptide | GRP | Cardiometabolic_II |
| P21754 | Zona pellucida sperm-binding protein 3 | ZP3 | Cardiometabolic_II |
| P07098 | Gastric triacylglycerol lipase | LIPF | Cardiometabolic_II |
| Q9H3K6 | BolA-like protein 2 | BOLA2_BOLA2B | Cardiometabolic_II |
| Q16621 | Transcription factor NF-E2 45 kDa subunit | NFE2 | Cardiometabolic_II |
| O94979 | Protein transport protein Sec31A | SEC31A | Cardiometabolic_II |
| P20042 | Eukaryotic translation initiation factor 2 subunit 2 | EIF2S2 | Cardiometabolic_II |
| Q9UJ70 | N-acetyl-D-glucosamine kinase | NAGK | Cardiometabolic_II |
| O95825 | Quinone oxidoreductase-like protein 1 | CRYZL1 | Cardiometabolic_II |
| Q6UWR7 | Glycerophosphocholine cholinephosphodiesterase ENPP6 | ENPP6 | Cardiometabolic_II |
| Q9BV79 | Enoyl-[acyl-carrier-protein] reductase, mitochondrial | MECR | Cardiometabolic_II |
| Q6UY14 | ADAMTS-like protein 4 | ADAMTSL4 | Cardiometabolic_II |
| Q5FWE3 | Proline-rich transmembrane protein 3 | PRRT3 | Cardiometabolic_II |
| Q8IWT1 | Sodium channel subunit beta-4 | SCN4B | Cardiometabolic_II |
| Q9HB40 | Retinoid-inducible serine carboxypeptidase | SCPEP1 | Cardiometabolic_II |
| Q96DR5 | BPI fold-containing family A member 2 | BPIFA2 | Cardiometabolic_II |
| P07942 | Laminin subunit beta-1 | LAMB1 | Cardiometabolic_II |
| Q8NFL0 | UDP-GlcNAc:betaGal beta-1,3-N-acetylglucosaminyltransferase 7 | B3GNT7 | Cardiometabolic_II |
| P01189 | Pro-opiomelanocortin | POMC | Cardiometabolic_II |
| Q7L266 | Isoaspartyl peptidase/L-asparaginase | ASRGL1 | Cardiometabolic_II |
| P50914 | 60S ribosomal protein L14 | RPL14 | Cardiometabolic_II |
| P09543 | 2',3'-cyclic-nucleotide 3'-phosphodiesterase | CNP | Cardiometabolic_II |
| P02458 | Collagen alpha-1(II) chain | COL2A1 | Cardiometabolic_II |
| Q24JP5 | Transmembrane protein 132A | TMEM132A | Cardiometabolic_II |
| P09681 | Gastric inhibitory polypeptide | GIP | Cardiometabolic_II |
| Q9UBQ7 | Glyoxylate reductase/hydroxypyruvate reductase | GRHPR | Cardiometabolic_II |
| Q9BVM4 | Gamma-glutamylaminecyclotransferase | GGACT | Cardiometabolic_II |
| P33681 | T-lymphocyte activation antigen CD80 | CD80 | Cardiometabolic_II |
| Q12982 | BCL2/adenovirus E1B 19 kDa protein-interacting protein 2 | BNIP2 | Cardiometabolic_II |
| Q96DC8 | Enoyl-CoA hydratase domain-containing protein 3, mitochondrial | ECHDC3 | Cardiometabolic_II |
| Q8TCD5 | 5'(3')-deoxyribonucleotidase, cytosolic type | NT5C | Cardiometabolic_II |
| Q7Z7M9 | Polypeptide N-acetylgalactosaminyltransferase 5 | GALNT5 | Cardiometabolic_II |
| Q00872 | Myosin-binding protein C, slow-type | MYBPC1 | Cardiometabolic_II |
| Q14914 | Prostaglandin reductase 1 | PTGR1 | Cardiometabolic_II |
| Q6UW49 | Sperm equatorial segment protein 1 | SPESP1 | Cardiometabolic_II |
| P51511 | Matrix metalloproteinase-15 | MMP15 | Cardiometabolic_II |
| P08138 | Tumor necrosis factor receptor superfamily member 16 | NGFR | Cardiometabolic_II |
| P15502 | Elastin | ELN | Cardiometabolic_II |
| Q8NDI1 | EH domain-binding protein 1 | EHBP1 | Cardiometabolic_II |
| O43681 | ATPase GET3 | GET3 | Cardiometabolic_II |
| O75223 | Gamma-glutamylcyclotransferase | GGCT | Cardiometabolic_II |
| O43405 | Cochlin | COCH | Cardiometabolic_II |
| Q9Y2E5 | Epididymis-specific alpha-mannosidase | MAN2B2 | Cardiometabolic_II |
| P02008 | Hemoglobin subunit zeta | HBZ | Cardiometabolic_II |
| P23919 | Thymidylate kinase | DTYMK | Cardiometabolic_II |
| Q7Z304 | MAM domain-containing protein 2 | MAMDC2 | Cardiometabolic_II |
| P78539 | Sushi repeat-containing protein SRPX | SRPX | Cardiometabolic_II |
| Q8N4F0 | BPI fold-containing family B member 2 | BPIFB2 | Cardiometabolic_II |
| P54687 | Branched-chain-amino-acid aminotransferase, cytosolic | BCAT1 | Cardiometabolic_II |
| Q969H8 | Myeloid-derived growth factor | MYDGF | Cardiometabolic_II |
| Q96EM0 | Trans-3-hydroxy-L-proline dehydratase | L3HYPDH | Cardiometabolic_II |
| Q9BXN1 | Asporin | ASPN | Cardiometabolic_II |
| Q13428 | Treacle protein | TCOF1 | Cardiometabolic_II |
| P14854 | Cytochrome c oxidase subunit 6B1 | COX6B1 | Cardiometabolic_II |
| P16035 | Metalloproteinase inhibitor 2 | TIMP2 | Cardiometabolic_II |
| P53674 | Beta-crystallin B1 | CRYBB1 | Cardiometabolic_II |
| O14960 | Leukocyte cell-derived chemotaxin-2 | LECT2 | Cardiometabolic_II |
| O14933 | Ubiquitin/ISG15-conjugating enzyme E2 L6 | UBE2L6 | Cardiometabolic_II |
| Q8N436 | Inactive carboxypeptidase-like protein X2 | CPXM2 | Cardiometabolic_II |
| Q13442 | 28 kDa heat- and acid-stable phosphoprotein | PDAP1 | Cardiometabolic_II |
| P23467 | Receptor-type tyrosine-protein phosphatase beta | PTPRB | Cardiometabolic_II |
| O75154 | Rab11 family-interacting protein 3 | RAB11FIP3 | Cardiometabolic_II |
| Q6NUS6 | Tectonic-3 | TCTN3 | Cardiometabolic_II |
| Q96FZ7 | Charged multivesicular body protein 6 | CHMP6 | Cardiometabolic_II |
| P98161 | Polycystin-1 | PKD1 | Cardiometabolic_II |
| Q9BXD5 | N-acetylneuraminate lyase | NPL | Cardiometabolic_II |
| Q9P2J2 | Protein turtle homolog A | IGSF9 | Cardiometabolic_II |
| Q9BW04 | Specifically androgen-regulated gene protein | SARG | Cardiometabolic_II |
| Q8WWV6 | High affinity immunoglobulin alpha and immunoglobulin mu Fc receptor | FCAMR | Cardiometabolic_II |
| O75711 | Scrapie-responsive protein 1 | SCRG1 | Cardiometabolic_II |
| Q6UXI7 | Vitrin | VIT | Cardiometabolic_II |
| P29692 | Elongation factor 1-delta | EEF1D | Cardiometabolic_II |
| Q9NQR4 | Omega-amidase NIT2 | NIT2 | Cardiometabolic_II |
| Q9BQS7 | Hephaestin | HEPH | Cardiometabolic_II |
| Q9H3S4 | Thiamin pyrophosphokinase 1 | TPK1 | Cardiometabolic_II |
| Q96C24 | Synaptotagmin-like protein 4 | SYTL4 | Cardiometabolic_II |
| O60234 | Glia maturation factor gamma | GMFG | Cardiometabolic_II |
| P51688 | N-sulphoglucosamine sulphohydrolase | SGSH | Cardiometabolic_II |
| Q969X0 | RILP-like protein 2 | RILPL2 | Cardiometabolic_II |
| P23471 | Receptor-type tyrosine-protein phosphatase zeta | PTPRZ1 | Cardiometabolic_II |
| P52209 | 6-phosphogluconate dehydrogenase, decarboxylating | PGD | Cardiometabolic_II |
| P32320 | Cytidine deaminase | CDA | Cardiometabolic_II |
| Q6PI73 | Leukocyte immunoglobulin-like receptor subfamily A member 6 | LILRA6 | Cardiometabolic_II |
| P08582 | Melanotransferrin | MELTF | Cardiometabolic_II |
| Q96MK3 | Pseudokinase FAM20A | FAM20A | Cardiometabolic_II |
| Q8IZF2 | Adhesion G protein-coupled receptor F5 | ADGRF5 | Cardiometabolic_II |
| P49593 | Protein phosphatase 1F | PPM1F | Cardiometabolic_II |
| P05413 | Fatty acid-binding protein, heart | FABP3 | Cardiometabolic_II |
| Q9UBR1 | Beta-ureidopropionase | UPB1 | Cardiometabolic_II |
| Q15388 | Mitochondrial import receptor subunit TOM20 homolog | TOMM20 | Cardiometabolic_II |
| O00194 | Ras-related protein Rab-27B | RAB27B | Cardiometabolic_II |
| Q6ZMM2 | ADAMTS-like protein 5 | ADAMTSL5 | Cardiometabolic_II |
| P45954 | Short/branched chain specific acyl-CoA dehydrogenase, mitochondrial | ACADSB | Cardiometabolic_II |
| P61026 | Ras-related protein Rab-10 | RAB10 | Cardiometabolic_II |
| P62072 | Mitochondrial import inner membrane translocase subunit Tim10 | TIMM10 | Cardiometabolic_II |
| P07093 | Glia-derived nexin | SERPINE2 | Cardiometabolic_II |
| Q7Z7K0 | COX assembly mitochondrial protein homolog | CMC1 | Cardiometabolic_II |
| Q96AG4 | Leucine-rich repeat-containing protein 59 | LRRC59 | Cardiometabolic_II |
| P40199 | Carcinoembryonic antigen-related cell adhesion molecule 6 | CEACAM6 | Cardiometabolic_II |
| P16410 | Cytotoxic T-lymphocyte protein 4 | CTLA4 | Cardiometabolic_II |
| P07288 | Prostate-specific antigen | KLK3 | Cardiometabolic_II |
| Q6H9L7 | Isthmin-2 | ISM2 | Cardiometabolic_II |
| Q9Y303 | N-acetylglucosamine-6-phosphate deacetylase | AMDHD2 | Cardiometabolic_II |
| P13796 | Plastin-2 | LCP1 | Cardiometabolic_II |
| P02730 | Band 3 anion transport protein | SLC4A1 | Cardiometabolic_II |
| Q86TH1 | ADAMTS-like protein 2 | ADAMTSL2 | Cardiometabolic_II |
| P30046 | D-dopachrome decarboxylase | DDT | Cardiometabolic_II |
| P23560 | Brain-derived neurotrophic factor | BDNF | Cardiometabolic_II |
| Q9NRR1 | Cytokine-like protein 1 | CYTL1 | Cardiometabolic_II |
| P13667 | Protein disulfide-isomerase A4 | PDIA4 | Cardiometabolic_II |
| Q8N6C8 | Leukocyte immunoglobulin-like receptor subfamily A member 3 | LILRA3 | Cardiometabolic_II |
| Q02817 | Mucin-2 | MUC2 | Cardiometabolic_II |
| P98095 | Fibulin-2 | FBLN2 | Cardiometabolic_II |
| P02461 | Collagen alpha-1(III) chain | COL3A1 | Cardiometabolic_II |
| P08575 | Receptor-type tyrosine-protein phosphatase C | PTPRC | Cardiometabolic_II |
| P13727 | Bone marrow proteoglycan | PRG2 | Cardiometabolic_II |
| P35579 | Myosin-9 | MYH9 | Cardiometabolic_II |
| Q9Y2Y8 | Proteoglycan 3 | PRG3 | Cardiometabolic_II |
| P32971 | Tumor necrosis factor ligand superfamily member 8 | TNFSF8 | Cardiometabolic_II |
| P30405 | Peptidyl-prolyl cis-trans isomerase F, mitochondrial | PPIF | Cardiometabolic_II |
| Q6IBS0 | Twinfilin-2 | TWF2 | Cardiometabolic_II |
| Q8N114 | Protein shisa-5 | SHISA5 | Cardiometabolic_II |
| O43280 | Trehalase | TREH | Cardiometabolic_II |
| P02818 | Osteocalcin | BGLAP | Cardiometabolic_II |
| P47972 | Neuronal pentraxin-2 | NPTX2 | Cardiometabolic_II |
| Q96NZ9 | Proline-rich acidic protein 1 | PRAP1 | Cardiometabolic_II |
| Q96CG8 | Collagen triple helix repeat-containing protein 1 | CTHRC1 | Cardiometabolic_II |
| Q9HCU0 | Endosialin | CD248 | Cardiometabolic_II |
| Q9BYJ0 | Fibroblast growth factor-binding protein 2 | FGFBP2 | Cardiometabolic_II |
| P39059 | Collagen alpha-1(XV) chain | COL15A1 | Cardiometabolic_II |
| Q6UVK1 | Chondroitin sulfate proteoglycan 4 | CSPG4 | Cardiometabolic_II |
| Q6UWP8 | Suprabasin | SBSN | Cardiometabolic_II |
| O75339 | Cartilage intermediate layer protein 1 | CILP | Cardiometabolic_II |
| P12277 | Creatine kinase B-type | CKB | Cardiometabolic_II |
| Q8WWQ8 | Stabilin-2 | STAB2 | Cardiometabolic_II |
| Q8TDY8 | Immunoglobulin superfamily DCC subclass member 4 | IGDCC4 | Cardiometabolic_II |
| P10645 | Chromogranin-A | CHGA | Cardiometabolic_II |
| P55000 | Secreted Ly-6/uPAR-related protein 1 | SLURP1 | Cardiometabolic_II |
| Q9H2X3 | C-type lectin domain family 4 member M | CLEC4M | Cardiometabolic_II |
| Q9Y646 | Carboxypeptidase Q | CPQ | Cardiometabolic_II |
| Q04721 | Neurogenic locus notch homolog protein 2 | NOTCH2 | Cardiometabolic_II |
| O95965 | Integrin beta-like protein 1 | ITGBL1 | Cardiometabolic_II |
| Q9Y251 | Heparanase | HPSE | Cardiometabolic_II |
| Q15063 | Periostin | POSTN | Cardiometabolic_II |
| P08217 | Chymotrypsin-like elastase family member 2A | CELA2A | Cardiometabolic_II |
| Q9UQP3 | Tenascin-N | TNN | Cardiometabolic_II |
| P17900 | Ganglioside GM2 activator | GM2A | Cardiometabolic_II |
| P37837 | Transaldolase | TALDO1 | Cardiometabolic_II |
| Q13510 | Acid ceramidase | ASAH1 | Cardiometabolic_II |
| P11279 | Lysosome-associated membrane glycoprotein 1 | LAMP1 | Cardiometabolic_II |
| P17174 | Aspartate aminotransferase, cytoplasmic | GOT1 | Cardiometabolic_II |
| P61916 | NPC intracellular cholesterol transporter 2 | NPC2 | Cardiometabolic_II |
| P07602 | Prosaposin | PSAP | Cardiometabolic_II |
| P60568 | Interleukin-2 | IL2 | Inflammation |
| Q13651 | Interleukin-10 receptor subunit alpha | IL10RA | Inflammation |
| Q13219 | Pappalysin-1 | PAPPA | Inflammation |
| Q9UHF4 | Interleukin-20 receptor subunit alpha | IL20RA | Inflammation |
| P63241 | Eukaryotic translation initiation factor 5A-1 | EIF5A | Inflammation |
| P05412 | Transcription factor AP-1 | JUN | Inflammation |
| Q96AX2 | Ras-related protein Rab-37 | RAB37 | Inflammation |
| P05112 | Interleukin-4 | IL4 | Inflammation |
| P01584 | Interleukin-1 beta | IL1B | Inflammation |
| O95760 | Interleukin-33 | IL33 | Inflammation |
| Q8NHJ6 | Leukocyte immunoglobulin-like receptor subfamily B member 4 | LILRB4 | Inflammation |
| P35225 | Interleukin-13 | IL13 | Inflammation |
| P22301 | Interleukin-10 | IL10 | Inflammation |
| P27540 | Aryl hydrocarbon receptor nuclear translocator | ARNT | Inflammation |
| O95379 | Tumor necrosis factor alpha-induced protein 8 | TNFAIP8 | Inflammation |
| Q8WV07 | Protein LTO1 homolog | LTO1 | Inflammation |
| O43707 | Alpha-actinin-4 | ACTN4 | Inflammation |
| P28838 | Cytosol aminopeptidase | LAP3 | Inflammation |
| Q9NP70 | Ameloblastin | AMBN | Inflammation |
| Q6UXK5 | Leucine-rich repeat neuronal protein 1 | LRRN1 | Inflammation |
| Q9HCU5 | Prolactin regulatory element-binding protein | PREB | Inflammation |
| Q13007 | Interleukin-24 | IL24 | Inflammation |
| Q9UPV0 | Centrosomal protein of 164 kDa | CEP164 | Inflammation |
| O60934 | Nibrin | NBN | Inflammation |
| Q96P31 | Fc receptor-like protein 3 | FCRL3 | Inflammation |
| Q9Y478 | 5'-AMP-activated protein kinase subunit beta-1 | PRKAB1 | Inflammation |
| Q8TCS8 | Polyribonucleotide nucleotidyltransferase 1, mitochondrial | PNPT1 | Inflammation |
| Q5T4W7 | Artemin | ARTN | Inflammation |
| Q5R372 | Rab GTPase-activating protein 1-like | RABGAP1L | Inflammation |
| Q969V3 | Nicalin | NCLN | Inflammation |
| Q8N6P7 | Interleukin-22 receptor subunit alpha-1 | IL22RA1 | Inflammation |
| P14784 | Interleukin-2 receptor subunit beta | IL2RB | Inflammation |
| Q13459 | Unconventional myosin-IXb | MYO9B | Inflammation |
| P19801 | Amiloride-sensitive amine oxidase [copper-containing] | AOC1 | Inflammation |
| Q9NYY1 | Interleukin-20 | IL20 | Inflammation |
| P57771 | Regulator of G-protein signaling 8 | RGS8 | Inflammation |
| P20809 | Interleukin-11 | IL11 | Inflammation |
| Q96PD4 | Interleukin-17F | IL17F | Inflammation |
| O76038 | Secretagogin | SCGN | Inflammation |
| O95715 | C-X-C motif chemokine 14 | CXCL14 | Inflammation |
| Q03426 | Mevalonate kinase | MVK | Inflammation |
| O14904 | Protein Wnt-9a | WNT9A | Inflammation |
| P26951 | Interleukin-3 receptor subunit alpha | IL3RA | Inflammation |
| Q9Y3P8 | Signaling threshold-regulating transmembrane adapter 1 | SIT1 | Inflammation |
| Q96DB9 | FXYD domain-containing ion transport regulator 5 | FXYD5 | Inflammation |
| P48061 | Stromal cell-derived factor 1 | CXCL12 | Inflammation |
| Q99748 | Neurturin | NRTN | Inflammation |
| Q13574 | Diacylglycerol kinase zeta | DGKZ | Inflammation |
| Q9Y2J8 | Protein-arginine deiminase type-2 | PADI2 | Inflammation |
| Q04759 | Protein kinase C theta type | PRKCQ | Inflammation |
| Q16552 | Interleukin-17A | IL17A | Inflammation |
| Q12968 | Nuclear factor of activated T-cells, cytoplasmic 3 | NFATC3 | Inflammation |
| Q14435 | Polypeptide N-acetylgalactosaminyltransferase 3 | GALNT3 | Inflammation |
| P05113 | Interleukin-5 | IL5 | Inflammation |
| P01375 | Tumor necrosis factor | TNF | Inflammation |
| Q92844 | TRAF family member-associated NF-kappa-B activator | TANK | Inflammation |
| O43597 | Protein sprouty homolog 2 | SPRY2 | Inflammation |
| P13693 | Translationally-controlled tumor protein | TPT1 | Inflammation |
| Q9P0M4 | Interleukin-17C | IL17C | Inflammation |
| Q7Z739 | YTH domain-containing family protein 3 | YTHDF3 | Inflammation |
| P42768 | Wiskott-Aldrich syndrome protein | WAS | Inflammation |
| Q96RJ3 | Tumor necrosis factor receptor superfamily member 13C | TNFRSF13C | Inflammation |
| Q8TAD2 | Interleukin-17D | IL17D | Inflammation |
| Q7Z6M3 | Allergin-1 | MILR1 | Inflammation |
| P30048 | Thioredoxin-dependent peroxide reductase, mitochondrial | PRDX3 | Inflammation |
| Q05084 | Islet cell autoantigen 1 | ICA1 | Inflammation |
| P51617 | Interleukin-1 receptor-associated kinase 1 | IRAK1 | Inflammation |
| P42701 | Interleukin-12 receptor subunit beta-1 | IL12RB1 | Inflammation |
| Q9HB29 | Interleukin-1 receptor-like 2 | IL1RL2 | Inflammation |
| P01583 | Interleukin-1 alpha | IL1A | Inflammation |
| P32456 | Guanylate-binding protein 2 | GBP2 | Inflammation |
| P12034 | Fibroblast growth factor 5 | FGF5 | Inflammation |
| P09919 | Granulocyte colony-stimulating factor | CSF3 | Inflammation |
| Q9BXJ7 | Protein amnionless | AMN | Inflammation |
| P18564 | Integrin beta-6 | ITGB6 | Inflammation |
| P01591 | Immunoglobulin J chain | JCHAIN | Inflammation |
| P01579 | Interferon gamma | IFNG | Inflammation |
| Q13291 | Signaling lymphocytic activation molecule | SLAMF1 | Inflammation |
| Q8N8S7 | Protein enabled homolog | ENAH | Inflammation |
| Q13261 | Interleukin-15 receptor subunit alpha | IL15RA | Inflammation |
| P09874 | Poly [ADP-ribose] polymerase 1 | PARP1 | Inflammation |
| Q0Z7S8 | Fatty acid-binding protein 9 | FABP9 | Inflammation |
| P78362 | SRSF protein kinase 2 | SRPK2 | Inflammation |
| P09038 | Fibroblast growth factor 2 | FGF2 | Inflammation |
| O43736 | Integral membrane protein 2A | ITM2A | Inflammation |
| O14867 | Transcription regulator protein BACH1 | BACH1 | Inflammation |
| Q8IU57 | Interferon lambda receptor 1 | IFNLR1 | Inflammation |
| Q12933 | TNF receptor-associated factor 2 | TRAF2 | Inflammation |
| Q06520 | Bile salt sulfotransferase | SULT2A1 | Inflammation |
| O60575 | Serine protease inhibitor Kazal-type 4 | SPINK4 | Inflammation |
| Q9Y5A7 | NEDD8 ultimate buster 1 | NUB1 | Inflammation |
| O60542 | Persephin | PSPN | Inflammation |
| P30838 | Aldehyde dehydrogenase, dimeric NADP-preferring | ALDH3A1 | Inflammation |
| O43521-2 | Bcl-2-like protein 11, Isoform BimL | BCL2L11 | Inflammation |
| O60880 | SH2 domain-containing protein 1A | SH2D1A | Inflammation |
| Q12778 | Forkhead box protein O1 | FOXO1 | Inflammation |
| Q7L8A9 | Tubulinyl-Tyr carboxypeptidase 1 | VASH1 | Inflammation |
| P55957 | BH3-interacting domain death agonist | BID | Inflammation |
| Q6UB28 | Methionine aminopeptidase 1D, mitochondrial | METAP1D | Inflammation |
| P01903 | HLA class II histocompatibility antigen, DR alpha chain | HLA-DRA | Inflammation |
| Q92609 | TBC1 domain family member 5 | TBC1D5 | Inflammation |
| P01588 | Erythropoietin | EPO | Inflammation |
| P80098 | C-C motif chemokine 7 | CCL7 | Inflammation |
| Q9UN19 | Dual adapter for phosphotyrosine and 3-phosphotyrosine and 3-phosphoinositide | DAPP1 | Inflammation |
| Q9UNE0 | Tumor necrosis factor receptor superfamily member EDAR | EDAR | Inflammation |
| Q9C035 | Tripartite motif-containing protein 5 | TRIM5 | Inflammation |
| Q8N608 | Inactive dipeptidyl peptidase 10 | DPP10 | Inflammation |
| P23229 | Integrin alpha-6 | ITGA6 | Inflammation |
| Q6DN72 | Fc receptor-like protein 6 | FCRL6 | Inflammation |
| P33241 | Lymphocyte-specific protein 1 | LSP1 | Inflammation |
| Q9UNK0 | Syntaxin-8 | STX8 | Inflammation |
| P13747 | HLA class I histocompatibility antigen, alpha chain E | HLA-E | Inflammation |
| P19474 | E3 ubiquitin-protein ligase TRIM21 | TRIM21 | Inflammation |
| O75475 | PC4 and SFRS1-interacting protein | PSIP1 | Inflammation |
| P13232 | Interleukin-7 | IL7 | Inflammation |
| Q8IVG5 | Sterile alpha motif domain-containing protein 9-like | SAMD9L | Inflammation |
| Q96LC7 | Sialic acid-binding Ig-like lectin 10 | SIGLEC10 | Inflammation |
| B1AKI9 | Isthmin-1 | ISM1 | Inflammation |
| Q6ZMH5 | Zinc transporter ZIP5 | SLC39A5 | Inflammation |
| P78410 | Butyrophilin subfamily 3 member A2 | BTN3A2 | Inflammation |
| P12872 | Promotilin | MLN | Inflammation |
| Q12765 | Secernin-1 | SCRN1 | Inflammation |
| P58294 | Prokineticin-1 | PROK1 | Inflammation |
| Q9Y6K9 | NF-kappa-B essential modulator | IKBKG | Inflammation |
| O95644 | Nuclear factor of activated T-cells, cytoplasmic 1 | NFATC1 | Inflammation |
| Q9Y258 | C-C motif chemokine 26 | CCL26 | Inflammation |
| Q8WTT0 | C-type lectin domain family 4 member C | CLEC4C | Inflammation |
| Q3KPI0 | Carcinoembryonic antigen-related cell adhesion molecule 21 | CEACAM21 | Inflammation |
| Q9BT73 | Proteasome assembly chaperone 3 | PSMG3 | Inflammation |
| P20849 | Collagen alpha-1(IX) chain | COL9A1 | Inflammation |
| Q9HD26 | Golgi-associated PDZ and coiled-coil motif-containing protein | GOPC | Inflammation |
| P52564 | Dual specificity mitogen-activated protein kinase kinase 6 | MAP2K6 | Inflammation |
| Q9H0P0 | Cytosolic 5'-nucleotidase 3A | NT5C3A | Inflammation |
| Q9NZN5 | Rho guanine nucleotide exchange factor 12 | ARHGEF12 | Inflammation |
| P42575 | Caspase-2 | CASP2 | Inflammation |
| Q9UHC6 | Contactin-associated protein-like 2 | CNTNAP2 | Inflammation |
| P45984 | Mitogen-activated protein kinase 9 | MAPK9 | Inflammation |
| P11274 | Breakpoint cluster region protein | BCR | Inflammation |
| Q9UDT6 | CAP-Gly domain-containing linker protein 2 | CLIP2 | Inflammation |
| Q14242 | P-selectin glycoprotein ligand 1 | SELPLG | Inflammation |
| P78310 | Coxsackievirus and adenovirus receptor | CXADR | Inflammation |
| P40933 | Interleukin-15 | IL15 | Inflammation |
| P05231 | Interleukin-6 | IL6 | Inflammation |
| P24071 | Immunoglobulin alpha Fc receptor | FCAR | Inflammation |
| Q01151 | CD83 antigen | CD83 | Inflammation |
| O76036 | Natural cytotoxicity triggering receptor 1 | NCR1 | Inflammation |
| P19878 | Neutrophil cytosol factor 2 | NCF2 | Inflammation |
| P23582 | C-type natriuretic peptide | NPPC | Inflammation |
| Q9NRJ3 | C-C motif chemokine 28 | CCL28 | Inflammation |
| P26022 | Pentraxin-related protein PTX3 | PTX3 | Inflammation |
| Q03431 | Parathyroid hormone/parathyroid hormone-related peptide receptor | PTH1R | Inflammation |
| Q9GZT9 | Egl nine homolog 1 | EGLN1 | Inflammation |
| Q9UMR7 | C-type lectin domain family 4 member A | CLEC4A | Inflammation |
| P13725 | Oncostatin-M | OSM | Inflammation |
| P28845 | Corticosteroid 11-beta-dehydrogenase isozyme 1 | HSD11B1 | Inflammation |
| P24394 | Interleukin-4 receptor subunit alpha | IL4R | Inflammation |
| Q9NWZ3 | Interleukin-1 receptor-associated kinase 4 | IRAK4 | Inflammation |
| Q14773 | Intercellular adhesion molecule 4 | ICAM4 | Inflammation |
| Q16698 | 2,4-dienoyl-CoA reductase, mitochondrial | DECR1 | Inflammation |
| O60449 | Lymphocyte antigen 75 | LY75 | Inflammation |
| Q9UKX5 | Integrin alpha-11 | ITGA11 | Inflammation |
| O15169 | Axin-1 | AXIN1 | Inflammation |
| P50995 | Annexin A11 | ANXA11 | Inflammation |
| P01730 | T-cell surface glycoprotein CD4 | CD4 | Inflammation |
| Q9NRM6 | Interleukin-17 receptor B | IL17RB | Inflammation |
| P01374 | Lymphotoxin-alpha | LTA | Inflammation |
| Q6ZUJ8 | Phosphoinositide 3-kinase adapter protein 1 | PIK3AP1 | Inflammation |
| P16455 | Methylated-DNA--protein-cysteine methyltransferase | MGMT | Inflammation |
| O94992 | Protein HEXIM1 | HEXIM1 | Inflammation |
| Q6UXB4 | C-type lectin domain family 4 member G | CLEC4G | Inflammation |
| P20783 | Neurotrophin-3 | NTF3 | Inflammation |
| O14788 | Tumor necrosis factor ligand superfamily member 11 | TNFSF11 | Inflammation |
| Q29980_Q29983 | MHC class I polypeptide-related sequence A_MHC class I polypeptide-related sequence B | MICB_MICA | Inflammation |
| Q8NDB2 | B-cell scaffold protein with ankyrin repeats | BANK1 | Inflammation |
| Q8TD46 | Cell surface glycoprotein CD200 receptor 1 | CD200R1 | Inflammation |
| P08727 | Keratin, type I cytoskeletal 19 | KRT19 | Inflammation |
| Q9HCM2 | Plexin-A4 | PLXNA4 | Inflammation |
| P28827 | Receptor-type tyrosine-protein phosphatase mu | PTPRM | Inflammation |
| P32970 | CD70 antigen | CD70 | Inflammation |
| P01135 | Protransforming growth factor alpha | TGFA | Inflammation |
| Q01344 | Interleukin-5 receptor subunit alpha | IL5RA | Inflammation |
| Q9NQ25 | SLAM family member 7 | SLAMF7 | Inflammation |
| P41217 | OX-2 membrane glycoprotein | CD200 | Inflammation |
| P10144 | Granzyme B | GZMB | Inflammation |
| P24001 | Interleukin-32 | IL32 | Inflammation |
| P42702 | Leukemia inhibitory factor receptor | LIFR | Inflammation |
| Q9UIB8 | SLAM family member 5 | CD84 | Inflammation |
| P20340 | Ras-related protein Rab-6A | RAB6A | Inflammation |
| Q8WXI8 | C-type lectin domain family 4 member D | CLEC4D | Inflammation |
| P10147 | C-C motif chemokine 3 | CCL3 | Inflammation |
| P50591 | Tumor necrosis factor ligand superfamily member 10 | TNFSF10 | Inflammation |
| O15455 | Toll-like receptor 3 | TLR3 | Inflammation |
| P80162 | C-X-C motif chemokine 6 | CXCL6 | Inflammation |
| Q08174 | Protocadherin-1 | PCDH1 | Inflammation |
| P37235 | Hippocalcin-like protein 1 | HPCAL1 | Inflammation |
| P29965 | CD40 ligand | CD40LG | Inflammation |
| Q07065 | Cytoskeleton-associated protein 4 | CKAP4 | Inflammation |
| P68106 | Peptidyl-prolyl cis-trans isomerase FKBP1B | FKBP1B | Inflammation |
| P22304 | Iduronate 2-sulfatase | IDS | Inflammation |
| O00273 | DNA fragmentation factor subunit alpha | DFFA | Inflammation |
| P01137 | Transforming growth factor beta-1 proprotein | TGFB1 | Inflammation |
| Q6UXB2 | C-X-C motif chemokine 17 | CXCL17 | Inflammation |
| Q9Y266 | Nuclear migration protein nudC | NUDC | Inflammation |
| O43508 | Tumor necrosis factor ligand superfamily member 12 | TNFSF12 | Inflammation |
| Q04637 | Eukaryotic translation initiation factor 4 gamma 1 | EIF4G1 | Inflammation |
| P35613 | Basigin | BSG | Inflammation |
| O60884 | DnaJ homolog subfamily A member 2 | DNAJA2 | Inflammation |
| Q9BZW8 | Natural killer cell receptor 2B4 | CD244 | Inflammation |
| Q12918 | Killer cell lectin-like receptor subfamily B member 1 | KLRB1 | Inflammation |
| P50452 | Serpin B8 | SERPINB8 | Inflammation |
| P10145 | Interleukin-8 | CXCL8 | Inflammation |
| Q13241 | Natural killer cells antigen CD94 | KLRD1 | Inflammation |
| Q14005 | Pro-interleukin-16 | IL16 | Inflammation |
| O94856 | Neurofascin | NFASC | Inflammation |
| P40259 | B-cell antigen receptor complex-associated protein beta chain | CD79B | Inflammation |
| Q9BXN2 | C-type lectin domain family 7 member A | CLEC7A | Inflammation |
| P20273 | B-cell receptor CD22 | CD22 | Inflammation |
| Q9UQV4 | Lysosome-associated membrane glycoprotein 3 | LAMP3 | Inflammation |
| Q96LA5 | Fc receptor-like protein 2 | FCRL2 | Inflammation |
| O43561 | Linker for activation of T-cells family member 1 | LAT | Inflammation |
| P36959 | GMP reductase 1 | GMPR | Inflammation |
| Q15661 | Tryptase alpha/beta-1 | TPSAB1 | Inflammation |
| Q96SB3 | Neurabin-2 | PPP1R9B | Inflammation |
| P30044 | Peroxiredoxin-5, mitochondrial | PRDX5 | Inflammation |
| P00813 | Adenosine deaminase | ADA | Inflammation |
| Q9Y6Q6 | Tumor necrosis factor receptor superfamily member 11A | TNFRSF11A | Inflammation |
| O95971 | CD160 antigen | CD160 | Inflammation |
| P14317 | Hematopoietic lineage cell-specific protein | HCLS1 | Inflammation |
| P30203 | T-cell differentiation antigen CD6 | CD6 | Inflammation |
| P15692 | Vascular endothelial growth factor A | VEGFA | Inflammation |
| O75077 | Disintegrin and metalloproteinase domain-containing protein 23 | ADAM23 | Inflammation |
| Q13478 | Interleukin-18 receptor 1 | IL18R1 | Inflammation |
| P43489 | Tumor necrosis factor receptor superfamily member 4 | TNFRSF4 | Inflammation |
| P02745 | Complement C1q subcomponent subunit A | C1QA | Inflammation |
| Q99616 | C-C motif chemokine 13 | CCL13 | Inflammation |
| P14210 | Hepatocyte growth factor | HGF | Inflammation |
| Q12866 | Tyrosine-protein kinase Mer | MERTK | Inflammation |
| O00253 | Agouti-related protein | AGRP | Inflammation |
| P43234 | Cathepsin O | CTSO | Inflammation |
| P49771 | Fms-related tyrosine kinase 3 ligand | FLT3LG | Inflammation |
| O43915 | Vascular endothelial growth factor D | VEGFD | Inflammation |
| P12544 | Granzyme A | GZMA | Inflammation |
| Q9H4D0 | Calsyntenin-2 | CLSTN2 | Inflammation |
| P48023 | Tumor necrosis factor ligand superfamily member 6 | FASLG | Inflammation |
| P29460 | Interleukin-12 subunit beta | IL12B | Inflammation |
| Q15517 | Corneodesmosin | CDSN | Inflammation |
| P51671 | Eotaxin | CCL11 | Inflammation |
| Q16719 | Kynureninase | KYNU | Inflammation |
| Q9HBG7 | T-lymphocyte surface antigen Ly-9 | LY9 | Inflammation |
| P78556 | C-C motif chemokine 20 | CCL20 | Inflammation |
| P03956 | Interstitial collagenase | MMP1 | Inflammation |
| P49763 | Placenta growth factor | PGF | Inflammation |
| O15444 | C-C motif chemokine 25 | CCL25 | Inflammation |
| Q07325 | C-X-C motif chemokine 9 | CXCL9 | Inflammation |
| Q92484 | Acid sphingomyelinase-like phosphodiesterase 3a | SMPDL3A | Inflammation |
| P21709 | Ephrin type-A receptor 1 | EPHA1 | Inflammation |
| P15260 | Interferon gamma receptor 1 | IFNGR1 | Inflammation |
| P53634 | Dipeptidyl peptidase 1 | CTSC | Inflammation |
| Q5ZPR3 | CD276 antigen | CD276 | Inflammation |
| Q9UJU6 | Drebrin-like protein | DBNL | Inflammation |
| P30613 | Pyruvate kinase PKLR | PKLR | Inflammation |
| O43639 | Cytoplasmic protein NCK2 | NCK2 | Inflammation |
| P35625 | Metalloproteinase inhibitor 3 | TIMP3 | Inflammation |
| P47712 | Cytosolic phospholipase A2 | PLA2G4A | Inflammation |
| O00585 | C-C motif chemokine 21 | CCL21 | Inflammation |
| P09238 | Stromelysin-2 | MMP10 | Inflammation |
| P11684 | Uteroglobin | SCGB1A1 | Inflammation |
| Q6UWV6 | Ectonucleotide pyrophosphatase/phosphodiesterase family member 7 | ENPP7 | Inflammation |
| Q9BZZ2 | Sialoadhesin | SIGLEC1 | Inflammation |
| P25116 | Proteinase-activated receptor 1 | F2R | Inflammation |
| P09326 | CD48 antigen | CD48 | Inflammation |
| P55773 | C-C motif chemokine 23 | CCL23 | Inflammation |
| Q14116 | Interleukin-18 | IL18 | Inflammation |
| P13236 | C-C motif chemokine 4 | CCL4 | Inflammation |
| Q08334 | Interleukin-10 receptor subunit beta | IL10RB | Inflammation |
| P02778 | C-X-C motif chemokine 10 | CXCL10 | Inflammation |
| P01133 | Pro-epidermal growth factor | EGF | Inflammation |
| O75462 | Cytokine receptor-like factor 1 | CRLF1 | Inflammation |
| P18510 | Interleukin-1 receptor antagonist protein | IL1RN | Inflammation |
| Q9NZV1 | Cysteine-rich motor neuron 1 protein | CRIM1 | Inflammation |
| O14836 | Tumor necrosis factor receptor superfamily member 13B | TNFRSF13B | Inflammation |
| Q9BY76 | Angiopoietin-related protein 4 | ANGPTL4 | Inflammation |
| O76096 | Cystatin-F | CST7 | Inflammation |
| P21860 | Receptor tyrosine-protein kinase erbB-3 | ERBB3 | Inflammation |
| Q99435 | Protein kinase C-binding protein NELL2 | NELL2 | Inflammation |
| P55145 | Mesencephalic astrocyte-derived neurotrophic factor | MANF | Inflammation |
| Q14210 | Lymphocyte antigen 6D | LY6D | Inflammation |
| P29279 | CCN family member 2 | CCN2 | Inflammation |
| Q9HC38 | Glyoxalase domain-containing protein 4 | GLOD4 | Inflammation |
| Q99685 | Monoglyceride lipase | MGLL | Inflammation |
| Q8NFT8 | Delta and Notch-like epidermal growth factor-related receptor | DNER | Inflammation |
| Q7KYR7 | Butyrophilin subfamily 2 member A1 | BTN2A1 | Inflammation |
| P34896 | Serine hydroxymethyltransferase, cytosolic | SHMT1 | Inflammation |
| O95750 | Fibroblast growth factor 19 | FGF19 | Inflammation |
| P19256 | Lymphocyte function-associated antigen 3 | CD58 | Inflammation |
| P29350 | Tyrosine-protein phosphatase non-receptor type 6 | PTPN6 | Inflammation |
| P0DMV8 | Heat shock 70 kDa protein 1A | HSPA1A | Inflammation |
| P09603 | Macrophage colony-stimulating factor 1 | CSF1 | Inflammation |
| O43291 | Kunitz-type protease inhibitor 2 | SPINT2 | Inflammation |
| P12532 | Creatine kinase U-type, mitochondrial | CKMT1A_CKMT1B | Inflammation |
| Q9Y3D6 | Mitochondrial fission 1 protein | FIS1 | Inflammation |
| Q96PL1 | Secretoglobin family 3A member 2 | SCGB3A2 | Inflammation |
| P25942 | Tumor necrosis factor receptor superfamily member 5 | CD40 | Inflammation |
| Q99983 | Osteomodulin | OMD | Inflammation |
| Q9UKU9 | Angiopoietin-related protein 2 | ANGPTL2 | Inflammation |
| P22466 | Galanin peptides | GAL | Inflammation |
| Q8WXD2 | Secretogranin-3 | SCG3 | Inflammation |
| Q9NR12 | PDZ and LIM domain protein 7 | PDLIM7 | Inflammation |
| Q9H3U7 | SPARC-related modular calcium-binding protein 2 | SMOC2 | Inflammation |
| Q9NZC2 | Triggering receptor expressed on myeloid cells 2 | TREM2 | Inflammation |
| Q8WU39 | Marginal zone B- and B1-cell-specific protein | MZB1 | Inflammation |
| O75888 | Tumor necrosis factor ligand superfamily member 13 | TNFSF13 | Inflammation |
| Q9Y6N7 | Roundabout homolog 1 | ROBO1 | Inflammation |
| O00300 | Tumor necrosis factor receptor superfamily member 11B | TNFRSF11B | Inflammation |
| Q9UJA9 | Ectonucleotide pyrophosphatase/phosphodiesterase family member 5 | ENPP5 | Inflammation |
| Q6GTX8 | Leukocyte-associated immunoglobulin-like receptor 1 | LAIR1 | Inflammation |
| Q5KU26 | Collectin-12 | COLEC12 | Inflammation |
| O00241 | Signal-regulatory protein beta-1 | SIRPB1 | Inflammation |
| Q15389 | Angiopoietin-1 | ANGPT1 | Inflammation |
| P01127 | Platelet-derived growth factor subunit B | PDGFB | Inflammation |
| P46109 | Crk-like protein | CRKL | Inflammation |
| P16422 | Epithelial cell adhesion molecule | EPCAM | Inflammation |
| O43598 | 2'-deoxynucleoside 5'-phosphate N-hydrolase 1 | DNPH1 | Inflammation |
| Q92583 | C-C motif chemokine 17 | CCL17 | Inflammation |
| Q96KG7 | Multiple epidermal growth factor-like domains protein 10 | MEGF10 | Inflammation |
| P24387 | Corticotropin-releasing factor-binding protein | CRHBP | Inflammation |
| P56470 | Galectin-4 | LGALS4 | Inflammation |
| Q9H008 | Phospholysine phosphohistidine inorganic pyrophosphate phosphatase | LHPP | Inflammation |
| O14773 | Tripeptidyl-peptidase 1 | TPP1 | Inflammation |
| Q6UXH1 | Protein disulfide isomerase CRELD2 | CRELD2 | Inflammation |
| Q99895 | Chymotrypsin-C | CTRC | Inflammation |
| Q9NQ76 | Matrix extracellular phosphoglycoprotein | MEPE | Inflammation |
| Q4KMG0 | Cell adhesion molecule-related/down-regulated by oncogenes | CDON | Inflammation |
| Q9UHX3 | Adhesion G protein-coupled receptor E2 | ADGRE2 | Inflammation |
| Q15109 | Advanced glycosylation end product-specific receptor | AGER | Inflammation |
| P27930 | Interleukin-1 receptor type 2 | IL1R2 | Inflammation |
| Q9NQ30 | Endothelial cell-specific molecule 1 | ESM1 | Inflammation |
| Q9HCB6 | Spondin-1 | SPON1 | Inflammation |
| Q9UII2 | ATPase inhibitor, mitochondrial | ATP5IF1 | Inflammation |
| O75563 | Src kinase-associated phosphoprotein 2 | SKAP2 | Inflammation |
| P09341 | Growth-regulated alpha protein | CXCL1 | Inflammation |
| Q16651 | Prostasin | PRSS8 | Inflammation |
| Q03405 | Urokinase plasminogen activator surface receptor | PLAUR | Inflammation |
| O00626 | C-C motif chemokine 22 | CCL22 | Inflammation |
| P51888 | Prolargin | PRELP | Inflammation |
| O00339 | Matrilin-2 | MATN2 | Inflammation |
| P36941 | Tumor necrosis factor receptor superfamily member 3 | LTBR | Inflammation |
| Q16363 | Laminin subunit alpha-4 | LAMA4 | Inflammation |
| P19883 | Follistatin | FST | Inflammation |
| Q9BU40 | Chordin-like protein 1 | CHRDL1 | Inflammation |
| O00175 | C-C motif chemokine 24 | CCL24 | Inflammation |
| Q99538 | Legumain | LGMN | Inflammation |
| Q14118 | Dystroglycan | DAG1 | Inflammation |
| P54317 | Pancreatic lipase-related protein 2 | PNLIPRP2 | Inflammation |
| Q8IYS5 | Osteoclast-associated immunoglobulin-like receptor | OSCAR | Inflammation |
| Q15166 | Serum paraoxonase/lactonase 3 | PON3 | Inflammation |
| P07148 | Fatty acid-binding protein, liver | FABP1 | Inflammation |
| O95866 | Megakaryocyte and platelet inhibitory receptor G6b | MPIG6B | Inflammation |
| P15291 | Beta-1,4-galactosyltransferase 1 | B4GALT1 | Inflammation |
| O00182 | Galectin-9 | LGALS9 | Inflammation |
| O95633 | Follistatin-related protein 3 | FSTL3 | Inflammation |
| Q92956 | Tumor necrosis factor receptor superfamily member 14 | TNFRSF14 | Inflammation |
| Q9BYZ8 | Regenerating islet-derived protein 4 | REG4 | Inflammation |
| Q8TEU8 | WAP, Kazal, immunoglobulin, Kunitz and NTR domain-containing protein 2 | WFIKKN2 | Inflammation |
| O00468 | Agrin | AGRN | Inflammation |
| Q03403 | Trefoil factor 2 | TFF2 | Inflammation |
| P19876 | C-X-C motif chemokine 3 | CXCL3 | Inflammation |
| Q13232 | Nucleoside diphosphate kinase 3 | NME3 | Inflammation |
| Q8N907 | DAN domain family member 5 | DAND5 | Inflammation |
| P24530 | Endothelin receptor type B | EDNRB | Inflammation |
| Q99665 | Interleukin-12 receptor subunit beta-2 | IL12RB2 | Inflammation |
| Q7Z698 | Sprouty-related, EVH1 domain-containing protein 2 | SPRED2 | Inflammation |
| Q9NRR2 | Tryptase gamma | TPSG1 | Inflammation |
| Q16665 | Hypoxia-inducible factor 1-alpha | HIF1A | Inflammation |
| Q9Y4C1 | Lysine-specific demethylase 3A | KDM3A | Inflammation |
| Q9H832 | Ubiquitin-conjugating enzyme E2 Z | UBE2Z | Inflammation |
| O00206 | Toll-like receptor 4 | TLR4 | Inflammation |
| P10070 | Zinc finger protein GLI2 | GLI2 | Inflammation |
| Q04609 | Glutamate carboxypeptidase 2 | FOLH1 | Inflammation |
| Q9NQI0 | Probable ATP-dependent RNA helicase DDX4 | DDX4 | Inflammation |
| Q13190 | Syntaxin-5 | STX5 | Inflammation |
| Q6R327 | Rapamycin-insensitive companion of mTOR | RICTOR | Inflammation |
| P03372 | Estrogen receptor | ESR1 | Inflammation |
| P41273 | Tumor necrosis factor ligand superfamily member 9 | TNFSF9 | Inflammation |
| Q9BY41 | Histone deacetylase 8 | HDAC8 | Inflammation |
| P43378 | Tyrosine-protein phosphatase non-receptor type 9 | PTPN9 | Inflammation |
| Q09472 | Histone acetyltransferase p300 | EP300 | Inflammation |
| Q96EB6 | NAD-dependent protein deacetylase sirtuin-1 | SIRT1 | Inflammation |
| P24928 | DNA-directed RNA polymerase II subunit RPB1 | POLR2A | Inflammation |
| Q9HBE5 | Interleukin-21 receptor | IL21R | Inflammation |
| Q92185 | Alpha-N-acetylneuraminide alpha-2,8-sialyltransferase | ST8SIA1 | Inflammation |
| P10767 | Fibroblast growth factor 6 | FGF6 | Inflammation |
| Q8WX93 | Palladin | PALLD | Inflammation |
| Q9UPW0 | Forkhead box protein J3 | FOXJ3 | Inflammation |
| P25490 | Transcriptional repressor protein YY1 | YY1 | Inflammation |
| Q6UXZ4 | Netrin receptor UNC5D | UNC5D | Inflammation |
| P09693 | T-cell surface glycoprotein CD3 gamma chain | CD3G | Inflammation |
| Q05329 | Glutamate decarboxylase 2 | GAD2 | Inflammation |
| Q6UXM1 | Leucine-rich repeats and immunoglobulin-like domains protein 3 | LRIG3 | Inflammation |
| P15531 | Nucleoside diphosphate kinase A | NME1 | Inflammation |
| P62834 | Ras-related protein Rap-1A | RAP1A | Inflammation |
| P15927 | Replication protein A 32 kDa subunit | RPA2 | Inflammation |
| P61328 | Fibroblast growth factor 12 | FGF12 | Inflammation |
| P46013 | Proliferation marker protein Ki-67 | MKI67 | Inflammation |
| P24864 | G1/S-specific cyclin-E1 | CCNE1 | Inflammation |
| P85299 | Proline-rich protein 5 | PRR5 | Inflammation |
| P49715 | CCAAT/enhancer-binding protein alpha | CEBPA | Inflammation |
| Q9NP95 | Fibroblast growth factor 20 | FGF20 | Inflammation |
| P06401 | Progesterone receptor | PGR | Inflammation |
| Q6UXL0 | Interleukin-20 receptor subunit beta | IL20RB | Inflammation |
| Q9NP85 | Podocin | NPHS2 | Inflammation |
| Q01201 | Transcription factor RelB | RELB | Inflammation |
| Q9NZS2 | Killer cell lectin-like receptor subfamily F member 1 | KLRF1 | Inflammation |
| Q9Y2I7 | 1-phosphatidylinositol 3-phosphate 5-kinase | PIKFYVE | Inflammation |
| Q9NWV8 | BRISC and BRCA1-A complex member 1 | BABAM1 | Inflammation |
| O00401 | Neural Wiskott-Aldrich syndrome protein | WASL | Inflammation |
| Q04837 | Single-stranded DNA-binding protein, mitochondrial | SSBP1 | Inflammation |
| O75365 | Protein tyrosine phosphatase type IVA 3 | PTP4A3 | Inflammation |
| Q12888 | TP53-binding protein 1 | TP53BP1 | Inflammation |
| O75173 | A disintegrin and metalloproteinase with thrombospondin motifs 4 | ADAMTS4 | Inflammation |
| Q9UHA7 | Interleukin-36 alpha | IL36A | Inflammation |
| Q96EP0 | E3 ubiquitin-protein ligase RNF31 | RNF31 | Inflammation |
| O95429 | BAG family molecular chaperone regulator 4 | BAG4 | Inflammation |
| Q8NI17 | Interleukin-31 receptor subunit alpha | IL31RA | Inflammation |
| Q9NS62 | Thrombospondin type-1 domain-containing protein 1 | THSD1 | Inflammation |
| Q9NY59 | Sphingomyelin phosphodiesterase 3 | SMPD3 | Inflammation |
| Q92574 | Hamartin | TSC1 | Inflammation |
| Q13114-2 | TNF receptor-associated factor 3, Isoform 2 | TRAF3 | Inflammation |
| P54274 | Telomeric repeat-binding factor 1 | TERF1 | Inflammation |
| Q96MM7 | Heparan-sulfate 6-O-sulfotransferase 2 | HS6ST2 | Inflammation |
| P35219 | Carbonic anhydrase-related protein | CA8 | Inflammation |
| Q9GZN4 | Brain-specific serine protease 4 | PRSS22 | Inflammation |
| Q9NQ66 | 1-phosphatidylinositol 4,5-bisphosphate phosphodiesterase beta-1 | PLCB1 | Inflammation |
| P16671 | Platelet glycoprotein 4 | CD36 | Inflammation |
| Q8NBK3 | Formylglycine-generating enzyme | SUMF1 | Inflammation |
| Q6EBC2 | Interleukin-31 | IL31 | Inflammation |
| Q8NHP1 | Aflatoxin B1 aldehyde reductase member 4 | AKR7L | Inflammation |
| Q9Y3D3 | 28S ribosomal protein S16, mitochondrial | MRPS16 | Inflammation |
| P98170 | E3 ubiquitin-protein ligase XIAP | XIAP | Inflammation |
| O43184 | Growth/differentiation factor 15 | ADAM12 | Inflammation |
| P48730 | Casein kinase I isoform delta | CSNK1D | Inflammation |
| O43583 | Density-regulated protein | DENR | Inflammation |
| O00148 | ATP-dependent RNA helicase DDX39A | DDX39A | Inflammation |
| P36551 | Oxygen-dependent coproporphyrinogen-III oxidase, mitochondrial | CPOX | Inflammation |
| Q96D71 | RalBP1-associated Eps domain-containing protein 1 | REPS1 | Inflammation |
| P09923 | Intestinal-type alkaline phosphatase | ALPI | Inflammation |
| Q5TBC7 | Bcl-2-like protein 15 | BCL2L15 | Inflammation |
| Q9UHN6 | Cell surface hyaluronidase | CEMIP2 | Inflammation |
| Q6B9Z1 | Insulin growth factor-like family member 4 | IGFL4 | Inflammation |
| Q8IX19 | Mast cell-expressed membrane protein 1 | MCEMP1 | Inflammation |
| P36897 | TGF-beta receptor type-1 | TGFBR1 | Inflammation |
| Q14511 | Enhancer of filamentation 1 | NEDD9 | Inflammation |
| Q8WVV4 | Protein POF1B | POF1B | Inflammation |
| Q9UIK4 | Death-associated protein kinase 2 | DAPK2 | Inflammation |
| Q8IYW5 | E3 ubiquitin-protein ligase RNF168 | RNF168 | Inflammation |
| Q93062 | RNA-binding protein with multiple splicing | RBPMS | Inflammation |
| Q15697 | Zinc finger protein 174 | ZNF174 | Inflammation |
| P17050 | Alpha-N-acetylgalactosaminidase | NAGA | Inflammation |
| Q96PX8 | SLIT and NTRK-like protein 1 | SLITRK1 | Inflammation |
| P56645 | Period circadian protein homolog 3 | PER3 | Inflammation |
| Q9H0U9 | Testis-specific Y-encoded-like protein 1 | TSPYL1 | Inflammation |
| P26436 | Acrosomal protein SP-10 | ACRV1 | Inflammation |
| Q5JS54 | Proteasome assembly chaperone 4 | PSMG4 | Inflammation |
| Q7Z5L3 | Complement C1q-like protein 2 | C1QL2 | Inflammation |
| Q15465 | Sonic hedgehog protein | SHH | Inflammation |
| P17643 | 5,6-dihydroxyindole-2-carboxylic acid oxidase | TYRP1 | Inflammation |
| Q8IV38 | Ankyrin repeat and MYND domain-containing protein 2 | ANKMY2 | Inflammation |
| O60447 | Ecotropic viral integration site 5 protein homolog | EVI5 | Inflammation |
| Q16718 | NADH dehydrogenase [ubiquinone] 1 alpha subcomplex subunit 5 | NDUFA5 | Inflammation |
| Q96PU5 | E3 ubiquitin-protein ligase NEDD4-like | NEDD4L | Inflammation |
| O94916 | Nuclear factor of activated T-cells 5 | NFAT5 | Inflammation |
| O95835 | Serine/threonine-protein kinase LATS1 | LATS1 | Inflammation |
| P24666 | Low molecular weight phosphotyrosine protein phosphatase | ACP1 | Inflammation |
| O60238 | BCL2/adenovirus E1B 19 kDa protein-interacting protein 3-like | BNIP3L | Inflammation |
| Q9H171 | Z-DNA-binding protein 1 | ZBP1 | Inflammation |
| Q99062 | Granulocyte colony-stimulating factor receptor | CSF3R | Inflammation |
| Q9UHI8 | A disintegrin and metalloproteinase with thrombospondin motifs 1 | ADAMTS1 | Inflammation |
| Q99584 | Protein S100-A13 | S100A13 | Inflammation |
| P55211 | Caspase-9 | CASP9 | Inflammation |
| Q9H3T2 | Semaphorin-6C | SEMA6C | Inflammation |
| Q15399 | Toll-like receptor 1 | TLR1 | Inflammation |
| Q9Y2X7 | ARF GTPase-activating protein GIT1 | GIT1 | Inflammation |
| P81534 | Beta-defensin 103 | DEFB103A_DEFB103B | Inflammation |
| O43320 | Fibroblast growth factor 16 | FGF16 | Inflammation |
| P52630 | Signal transducer and activator of transcription 2 | STAT2 | Inflammation |
| P20701 | Integrin alpha-L | ITGAL | Inflammation |
| Q86UE4 | Protein LYRIC | MTDH | Inflammation |
| Q96PL5 | Erythroid membrane-associated protein | ERMAP | Inflammation |
| O60500 | Nephrin | NPHS1 | Inflammation |
| Q8WV28 | B-cell linker protein | BLNK | Inflammation |
| P48546 | Gastric inhibitory polypeptide receptor | GIPR | Inflammation |
| P09564 | T-cell antigen CD7 | CD7 | Inflammation |
| P11487 | Fibroblast growth factor 3 | FGF3 | Inflammation |
| Q15223 | Nectin-1 | NECTIN1 | Inflammation |
| P23743 | Diacylglycerol kinase alpha | DGKA | Inflammation |
| P29536 | Leiomodin-1 | LMOD1 | Inflammation |
| O75688 | Protein phosphatase 1B | PPM1B | Inflammation |
| Q16643 | Drebrin | DBN1 | Inflammation |
| Q5T2W1 | Na(+)/H(+) exchange regulatory cofactor NHE-RF3 | PDZK1 | Inflammation |
| Q9NZH8 | Interleukin-36 gamma | IL36G | Inflammation |
| Q5QGZ9 | C-type lectin domain family 12 member A | CLEC12A | Inflammation |
| P32927 | Cytokine receptor common subunit beta | CSF2RB | Inflammation |
| P49765 | Vascular endothelial growth factor B | VEGFB | Inflammation |
| Q9BV40 | Vesicle-associated membrane protein 8 | VAMP8 | Inflammation |
| Q15762 | CD226 antigen | CD226 | Inflammation |
| P06730 | Eukaryotic translation initiation factor 4E | EIF4E | Inflammation |
| Q14160 | Protein scribble homolog | SCRIB | Inflammation |
| Q02880 | DNA topoisomerase 2-beta | TOP2B | Inflammation |
| O15400 | Syntaxin-7 | STX7 | Inflammation |
| Q9H7Z7 | Prostaglandin E synthase 2 | PTGES2 | Inflammation |
| P14902 | Indoleamine 2,3-dioxygenase 1 | IDO1 | Inflammation |
| O60437 | Periplakin | PPL | Inflammation |
| O95157 | Neurexophilin-3 | NXPH3 | Inflammation |
| P06213 | Insulin receptor | INSR | Inflammation |
| P11234 | Ras-related protein Ral-B | RALB | Inflammation |
| P49757 | Protein numb homolog | NUMB | Inflammation |
| Q8N556 | Actin filament-associated protein 1 | AFAP1 | Inflammation |
| P17301 | Integrin alpha-2 | ITGA2 | Inflammation |
| Q02952 | A-kinase anchor protein 12 | AKAP12 | Inflammation |
| Q4VCS5 | Angiomotin | AMOT | Inflammation |
| P30101 | Protein disulfide-isomerase A3 | PDIA3 | Inflammation |
| P10912 | Growth hormone receptor | GHR | Inflammation |
| O95393 | Bone morphogenetic protein 10 | BMP10 | Inflammation |
| Q9UGN4 | CMRF35-like molecule 8 | CD300A | Inflammation |
| Q96QR1 | Secretoglobin family 3A member 1 | SCGB3A1 | Inflammation |
| P30040 | Endoplasmic reticulum resident protein 29 | ERP29 | Inflammation |
| Q9ULI3 | Protein HEG homolog 1 | HEG1 | Inflammation |
| Q8TEA8 | D-aminoacyl-tRNA deacylase 1 | DTD1 | Inflammation |
| P01037 | Cystatin-SN | CST1 | Inflammation |
| Q9H7Y0 | Divergent protein kinase domain 2B | DIPK2B | Inflammation |
| Q9BRK3 | Matrix remodeling-associated protein 8 | MXRA8 | Inflammation |
| O76074 | cGMP-specific 3',5'-cyclic phosphodiesterase | PDE5A | Inflammation |
| P22303 | Acetylcholinesterase | ACHE | Inflammation |
| Q9BX67 | Junctional adhesion molecule C | JAM3 | Inflammation |
| P20908 | Collagen alpha-1(V) chain | COL5A1 | Inflammation |
| Q6QNK2 | Adhesion G-protein coupled receptor D1 | ADGRD1 | Inflammation |
| Q02223 | Tumor necrosis factor receptor superfamily member 17 | TNFRSF17 | Inflammation |
| Q8NEU8 | DCC-interacting protein 13-beta | APPL2 | Inflammation |
| Q9BUH6 | Protein PAXX | PAXX | Inflammation |
| Q8NDA2 | Hemicentin-2 | HMCN2 | Inflammation |
| Q9HCU4 | Cadherin EGF LAG seven-pass G-type receptor 2 | CELSR2 | Inflammation |
| P06280 | Alpha-galactosidase A | GLA | Inflammation |
| Q9BUN1 | Protein MENT | MENT | Inflammation |
| Q9H939 | Proline-serine-threonine phosphatase-interacting protein 2 | PSTPIP2 | Inflammation |
| Q8TDQ7 | Glucosamine-6-phosphate isomerase 2 | GNPDA2 | Inflammation |
| P55083 | Microfibril-associated glycoprotein 4 | MFAP4 | Inflammation |
| Q9NS98 | Semaphorin-3G | SEMA3G | Inflammation |
| P04406 | Glyceraldehyde-3-phosphate dehydrogenase | GAPDH | Inflammation |
| P04090 | Prorelaxin H2 | RLN2 | Inflammation |
| Q5TDH0 | Protein DDI1 homolog 2 | DDI2 | Inflammation |
| P06132 | Uroporphyrinogen decarboxylase | UROD | Inflammation |
| Q99574 | Neuroserpin | SERPINI1 | Inflammation |
| P27348 | 14-3-3 protein theta | YWHAQ | Inflammation |
| Q9UI42 | Carboxypeptidase A4 | CPA4 | Inflammation |
| Q16378 | Proline-rich protein 4 | PRR4 | Inflammation |
| Q6P5S2 | Protein LEG1 homolog | LEG1 | Inflammation |
| O43399 | Tumor protein D54 | TPD52L2 | Inflammation |
| P61457 | Pterin-4-alpha-carbinolamine dehydratase | PCBD1 | Inflammation |
| P40197 | Platelet glycoprotein V | GP5 | Inflammation |
| P00325 | All-trans-retinol dehydrogenase | ADH1B | Inflammation |
| O75190 | DnaJ homolog subfamily B member 6 | DNAJB6 | Inflammation |
| Q5JS37 | NHL repeat-containing protein 3 | NHLRC3 | Inflammation |
| Q8N8U9 | BMP-binding endothelial regulator protein | BMPER | Inflammation |
| O75347 | Tubulin-specific chaperone A | TBCA | Inflammation |
| P04083 | Annexin A1 | ANXA1 | Inflammation |
| Q9BXJ0 | Complement C1q tumor necrosis factor-related protein 5 | C1QTNF5 | Inflammation |
| Q96HD1 | Protein disulfide isomerase CRELD1 | CRELD1 | Inflammation |
| P0C862 | Complement C1q and tumor necrosis factor-related protein 9A | C1QTNF9 | Inflammation |
| Q13976 | cGMP-dependent protein kinase 1 | PRKG1 | Inflammation |
| P54764 | Ephrin type-A receptor 4 | EPHA4 | Inflammation |
| Q9P2T1 | GMP reductase 2 | GMPR2 | Inflammation |
| P50502 | Hsc70-interacting protein | ST13 | Inflammation |
| P09529 | Inhibin beta B chain | INHBB | Inflammation |
| A2VDF0 | Fucose mutarotase | FUOM | Inflammation |
| P20155 | Serine protease inhibitor Kazal-type 2 | SPINK2 | Inflammation |
| P14091 | Cathepsin E | CTSE | Inflammation |
| Q6P589 | Tumor necrosis factor alpha-induced protein 8-like protein 2 | TNFAIP8L2 | Inflammation |
| O14745 | Na(+)/H(+) exchange regulatory cofactor NHE-RF1 | SLC9A3R1 | Inflammation |
| P30047 | GTP cyclohydrolase 1 feedback regulatory protein | GCHFR | Inflammation |
| Q53FA7 | Quinone oxidoreductase PIG3 | TP53I3 | Inflammation |
| Q5VTT5 | Myomesin-3 | MYOM3 | Inflammation |
| Q8IUZ5 | 5-phosphohydroxy-L-lysine phospho-lyase | PHYKPL | Inflammation |
| P52758 | 2-iminobutanoate/2-iminopropanoate deaminase | RIDA | Inflammation |
| Q96A49 | Synapse-associated protein 1 | SYAP1 | Inflammation |
| Q9NQ48 | Leucine zipper transcription factor-like protein 1 | LZTFL1 | Inflammation |
| O96007 | Molybdopterin synthase catalytic subunit | MOCS2 | Inflammation |
| Q8NHV1 | GTPase IMAP family member 7 | GIMAP7 | Inflammation |
| Q96AJ9 | Vesicle transport through interaction with t-SNAREs homolog 1A | VTI1A | Inflammation |
| P21854 | B-cell differentiation antigen CD72 | CD72 | Inflammation |
| P07311 | Acylphosphatase-1 | ACYP1 | Inflammation |
| Q9UK23 | N-acetylglucosamine-1-phosphodiester alpha-N-acetylglucosaminidase | NAGPA | Inflammation |
| Q9NRS6 | Sorting nexin-15 | SNX15 | Inflammation |
| P37840 | Alpha-synuclein | SNCA | Inflammation |
| P22455 | Fibroblast growth factor receptor 4 | FGFR4 | Inflammation |
| Q15276 | Rab GTPase-binding effector protein 1 | RABEP1 | Inflammation |
| Q6FHJ7 | Secreted frizzled-related protein 4 | SFRP4 | Inflammation |
| Q9HB71 | Calcyclin-binding protein | CACYBP | Inflammation |
| O60279 | Sushi domain-containing protein 5 | SUSD5 | Inflammation |
| A6NC86 | phospholipase A2 inhibitor and Ly6/PLAUR domain-containing protein | PINLYP | Inflammation |
| P49862 | Kallikrein-7 | KLK7 | Inflammation |
| Q92619 | Rho GTPase-activating protein 45 | ARHGAP45 | Inflammation |
| Q04323 | UBX domain-containing protein 1 | UBXN1 | Inflammation |
| P35611 | Alpha-adducin | ADD1 | Inflammation |
| Q8N0X7 | Spartin | SPART | Inflammation |
| P32321 | Deoxycytidylate deaminase | DCTD | Inflammation |
| P56192 | Methionine--tRNA ligase, cytoplasmic | MARS1 | Inflammation |
| Q6GMV3 | Putative peptidyl-tRNA hydrolase PTRHD1 | PTRHD1 | Inflammation |
| O15335 | Chondroadherin | CHAD | Inflammation |
| P25686 | DnaJ homolog subfamily B member 2 | DNAJB2 | Inflammation |
| Q58EX2 | Protein sidekick-2 | SDK2 | Inflammation |
| Q86SX6 | Glutaredoxin-related protein 5, mitochondrial | GLRX5 | Inflammation |
| Q9Y4D1 | Disheveled-associated activator of morphogenesis 1 | DAAM1 | Inflammation |
| Q6UX06 | Olfactomedin-4 | OLFM4 | Inflammation |
| P01210 | Proenkephalin-A | PENK | Inflammation |
| P0DML2 | Chorionic somatomammotropin hormone 1 | CSH1 | Inflammation |
| P32119 | Peroxiredoxin-2 | PRDX2 | Inflammation |
| P40925 | Malate dehydrogenase, cytoplasmic | MDH1 | Inflammation |
| P10599 | Thioredoxin | TXN | Inflammation |
| P0DN86 | Choriogonadotropin subunit beta 3 | CGB3_CGB5_CGB8 | Inflammation |
| P07998 | Ribonuclease pancreatic | RNASE1 | Inflammation |
| P04114 | Apolipoprotein B-100 | APOB | Inflammation |
| Q92686 | Neurogranin | NRGN | Inflammation |
| P16442 | Histo-blood group ABO system transferase | ABO | Inflammation |
| Q6UXB8 | Peptidase inhibitor 16 | PI16 | Inflammation |
| P04180 | Phosphatidylcholine-sterol acyltransferase | LCAT | Inflammation |
| Q0ZGT2 | Nexilin | NEXN | Inflammation |
| P07307 | Asialoglycoprotein receptor 2 | ASGR2 | Inflammation |
| P06744 | Glucose-6-phosphate isomerase | GPI | Inflammation |
| P07333 | Macrophage colony-stimulating factor 1 receptor | CSF1R | Inflammation |
| Q86YW5 | Trem-like transcript 1 protein | TREML1 | Inflammation |
| P08294 | Extracellular superoxide dismutase | SOD3 | Inflammation |
| P00742 | Coagulation factor X | F10 | Inflammation |
| P04278 | Sex hormone-binding globulin | SHBG | Inflammation |
| P02652 | Apolipoprotein A-II | APOA2 | Inflammation |
| P20061 | Transcobalamin-1 | TCN1 | Inflammation |
| P12955 | Xaa-Pro dipeptidase | PEPD | Inflammation |
| Q93091 | Ribonuclease K6 | RNASE6 | Inflammation |
| O00602 | Ficolin-1 | FCN1 | Inflammation |
| O43493 | Trans-Golgi network integral membrane protein 2 | TGOLN2 | Inflammation |
| P50552 | Vasodilator-stimulated phosphoprotein | VASP | Inflammation |
| P16233 | Pancreatic triacylglycerol lipase | PNLIP | Inflammation |
| P22897 | Macrophage mannose receptor 1 | MRC1 | Inflammation |
| P12821 | Angiotensin-converting enzyme | ACE | Inflammation |
| P17927 | Complement receptor type 1 | CR1 | Inflammation |
| P02748 | Complement component C9 | C9 | Inflammation |
| P0DJD7 | Pepsin A-4 | PGA4 | Inflammation |
| P00390 | Glutathione reductase, mitochondrial | GSR | Inflammation |
| Q13790 | Apolipoprotein F | APOF | Inflammation |
| Q08830 | Fibrinogen-like protein 1 | FGL1 | Inflammation |
| P09172 | Dopamine beta-hydroxylase | DBH | Inflammation |
| P27169 | Serum paraoxonase/arylesterase 1 | PON1 | Inflammation |
| P34096 | Ribonuclease 4 | RNASE4 | Inflammation |
| P02765 | Alpha-2-HS-glycoprotein | AHSG | Inflammation |
| P0DUB6_P0DTE7_P0DTE8 | Alpha-amylase 1A_Alpha-amylase 1B_Alpha-amylase 1C | AMY1A_AMY1B_AMY1C | Inflammation |
| O14791 | Apolipoprotein L1 | APOL1 | Inflammation |
| P01019 | Angiotensinogen | AGT | Inflammation |
| Q01459 | Di-N-acetylchitobiase | CTBS | Inflammation |
| O95497 | Pantetheinase | VNN1 | Inflammation |
| P04040 | Catalase | CAT | Inflammation |
| P26927 | Hepatocyte growth factor-like protein | MST1 | Inflammation |
| P06396 | Gelsolin | GSN | Inflammation |
| P54108 | Cysteine-rich secretory protein 3 | CRISP3 | Inflammation |
| Q9BXR6 | Complement factor H-related protein 5 | CFHR5 | Inflammation |
| P06276 | Cholinesterase | BCHE | Inflammation |
| Q04756 | Hepatocyte growth factor activator | HGFAC | Inflammation |
| P07358 | Complement component C8 beta chain | C8B | Inflammation |
| P36980 | Complement factor H-related protein 2 | CFHR2 | Inflammation |
| Q9NZP8 | Complement C1r subcomponent-like protein | C1RL | Inflammation |
| P19827 | Inter-alpha-trypsin inhibitor heavy chain H1 | ITIH1 | Inflammation |
| P00734 | Prothrombin | F2 | Inflammation |
| P05543 | Thyroxine-binding globulin | SERPINA7 | Inflammation |
| P61769 | Beta-2-microglobulin | B2M | Inflammation |
| P35542 | Serum amyloid A-4 protein | SAA4 | Inflammation |
| P02649 | Apolipoprotein E | APOE | Inflammation |
| Q9Y5Y7 | Lymphatic vessel endothelial hyaluronic acid receptor 1 | LYVE1 | Inflammation |
| O00391 | Sulfhydryl oxidase 1 | QSOX1 | Inflammation |
| P20742 | Pregnancy zone protein | PZP | Inflammation |
| P09871 | Complement C1s subcomponent | C1S | Inflammation |
| P10909 | Clusterin | CLU | Inflammation |
| P06727 | Apolipoprotein A-IV | APOA4 | Inflammation |
| Q96IY4 | Carboxypeptidase B2 | CPB2 | Inflammation |
| O75882-2 | Attractin, Isoform 2 | ATRN | Inflammation |
| Q16610 | Extracellular matrix protein 1 | ECM1 | Inflammation |
| P07225 | Vitamin K-dependent protein S | PROS1 | Inflammation |
| P00746 | Complement factor D | CFD | Inflammation |
| P00748 | Coagulation factor XII | F12 | Inflammation |
| P0DOY2 | Immunoglobulin lambda constant 2 | IGLC2 | Inflammation |
| P43652 | Afamin | AFM | Inflammation |
| Q96PD5 | N-acetylmuramoyl-L-alanine amidase | PGLYRP2 | Inflammation |
| P08185 | Corticosteroid-binding globulin | SERPINA6 | Inflammation |
| P01031 | Complement C5 | C5 | Inflammation |
| P49908 | Selenoprotein P | SELENOP | Inflammation |
| P05090 | Apolipoprotein D | APOD | Inflammation |
| P08519 | Apolipoprotein(a) | LPA | Inflammation |
| Q15848 | Adiponectin | ADIPOQ | Inflammation |
| P02654 | Apolipoprotein C-I | APOC1 | Inflammation |
| P01009 | Alpha-1-antitrypsin | SERPINA1 | Inflammation |
| P02750 | Leucine-rich alpha-2-glycoprotein | LRG1 | Inflammation |
| P14151 | L-selectin | SELL | Inflammation |
| P00736 | Complement C1r subcomponent | C1R | Inflammation |
| P43251 | Biotinidase | BTD | Inflammation |
| P00751 | Complement factor B | CFB | Inflammation |
| P05452 | Tetranectin | CLEC3B | Inflammation |
| P36955 | Pigment epithelium-derived factor | SERPINF1 | Inflammation |
| Q92496 | Complement factor H-related protein 4 | CFHR4 | Inflammation |
| P11226 | Mannose-binding protein C | MBL2 | Inflammation |
| P03952 | Plasma kallikrein | KLKB1 | Inflammation |
| P02775 | Platelet basic protein | PPBP | Inflammation |
| Q14624 | Inter-alpha-trypsin inhibitor heavy chain H4 | ITIH4 | Inflammation |
| P05154 | Plasma serine protease inhibitor | SERPINA5 | Inflammation |
| P02776 | Platelet factor 4 | PF4 | Inflammation |
| Q08380 | Galectin-3-binding protein | LGALS3BP | Inflammation |
| P10643 | Complement component C7 | C7 | Inflammation |
| P05546 | Heparin cofactor 2 | SERPIND1 | Inflammation |
| P02763 | Alpha-1-acid glycoprotein 1 | ORM1 | Inflammation |
| P02647 | Apolipoprotein A-I | APOA1 | Inflammation |
| P04196 | Histidine-rich glycoprotein | HRG | Inflammation |
| P04217 | Alpha-1B-glycoprotein | A1BG | Inflammation |
| P05156 | Complement factor I | CFI | Inflammation |
| P03951 | Coagulation factor XI | F11 | Inflammation |
| P29622 | Kallistatin | SERPINA4 | Inflammation |
| O43866 | CD5 antigen-like | CD5L | Inflammation |
| P01024 | Complement C3 | C3 | Inflammation |
| P05155 | Plasma protease C1 inhibitor | SERPING1 | Inflammation |
| P02743 | Serum amyloid P-component | APCS | Inflammation |
| P08697 | Alpha-2-antiplasmin | SERPINF2 | Inflammation |
| P02774 | Vitamin D-binding protein | GC | Inflammation |
| P05160 | Coagulation factor XIII B chain | F13B | Inflammation |
| P02766 | Transthyretin | TTR | Inflammation |
| P02787 | Serotransferrin | TF | Inflammation |
| P27918 | Properdin | CFP | Inflammation |
| P00747 | Plasminogen | PLG | Inflammation |
| P01011 | Alpha-1-antichymotrypsin | SERPINA3 | Inflammation |
| P02751 | Fibronectin | FN1 | Inflammation |
| P02671 | Fibrinogen alpha chain | FGA | Inflammation |
| P01008 | Antithrombin-III | SERPINC1 | Inflammation |
| P08603 | Complement factor H | CFH | Inflammation |

**Caption:** Protein description, including their UniProt ID, protein name, gene name and the explore panel to which they belong.

**Supplementary table 3.** Linear mixed-effects models to assess associations of proteins at ~62 years old with BMI changes during adulthood (from 24 to 62 years old).

| **Protein description** | **Protein ID** | **Estimate** | **SE** | **p value** | | Protein  panel |
| --- | --- | --- | --- | --- | --- | --- |
|  |  |  |  | **Nominal** | **Bonferroni** |  |
| Ectonucleotide pyrophosphatase/phosphodiesterase family member 7 | Q6UWV6 | 0.024 | 0.004 | 8.42e-08 | 0.000104 | Inflammation |
| Coagulation factor IX | P00740 | 0.108 | 0.02 | 8.75e-08 | 0.000108 | Cardiometabolic |
| Thrombospondin-4 | P35443 | 0.046 | 0.009 | 1.12e-07 | 0.000138 | Cardiometabolic |
| Lysosomal Pro-X carboxypeptidase | P42785 | 0.072 | 0.013 | 1.32e-07 | 0.000163 | Cardiometabolic |
| Platelet glycoprotein 4 | P16671 | 0.073 | 0.014 | 1.41e-07 | 0.000174 | Inflammation |
| C-C motif chemokine 16 | O15467 | 0.051 | 0.01 | 1.61e-07 | 0.000198 | Cardiometabolic |
| Complement factor D | P00746 | 0.108 | 0.02 | 1.84e-07 | 0.000227 | Inflammation |
| Angiotensin-converting enzyme 2 | Q9BYF1 | 0.039 | 0.007 | 2.29e-07 | 0.000282 | Cardiometabolic |
| SLIT and NTRK-like protein 1 | Q96PX8 | -0.069 | 0.013 | 2.57e-07 | 0.000317 | Inflammation |
| Angiomotin | Q4VCS5 | 0.047 | 0.009 | 2.72e-07 | 0.000335 | Inflammation |
| Macrophage colony-stimulating factor 1 | P09603 | 0.091 | 0.017 | 2.82e-07 | 0.000347 | Inflammation |
| Fructose-1,6-bisphosphatase 1 | P09467 | 0.027 | 0.005 | 2.96e-07 | 0.000365 | Cardiometabolic |
| Neurexophilin-3 | O95157 | -0.067 | 0.013 | 3.64e-07 | 0.000448 | Inflammation |
| BPI fold-containing family B member 1 | Q8TDL5 | -0.033 | 0.006 | 4.09e-07 | 0.000504 | Cardiometabolic |
| N-acetylneuraminate lyase | Q9BXD5 | 0.047 | 0.009 | 4.12e-07 | 0.000508 | Cardiometabolic |
| Collectin-12 | Q5KU26 | 0.095 | 0.018 | 4.19e-07 | 0.000516 | Inflammation |
| Dickkopf-related protein 3 | Q9UBP4 | -0.067 | 0.013 | 4.31e-07 | 0.000532 | Cardiometabolic |
| Macrophage colony-stimulating factor 1 receptor | P07333 | 0.047 | 0.009 | 4.64e-07 | 0.000572 | Inflammation |
| Glycerophosphocholine cholinephosphodiesterase ENPP6 | Q6UWR7 | -0.057 | 0.011 | 4.77e-07 | 0.000587 | Cardiometabolic |
| Leptin receptor | P48357 | -0.08 | 0.016 | 5.66e-07 | 0.000698 | Cardiometabolic |
| Somatotropin | P01241 | -0.012 | 0.002 | 5.91e-07 | 0.000728 | Cardiometabolic |
| Tissue-type plasminogen activator | P00750 | 0.03 | 0.006 | 6.45e-07 | 0.000795 | Cardiometabolic |
| Gamma-glutamyl hydrolase | Q92820 | 0.057 | 0.011 | 6.64e-07 | 0.000818 | Cardiometabolic |
| Phospholipid transfer protein | P55058 | -0.063 | 0.012 | 7.34e-07 | 0.000904 | Cardiometabolic |
| Dermatopontin | Q07507 | 0.08 | 0.016 | 7.55e-07 | 0.00093 | Cardiometabolic |
| Alpha-amylase 1A_Alpha-amylase 1B_Alpha-amylase 1C | P0DUB6_P0DTE7_P0DTE8 | -0.047 | 0.009 | 8.46e-07 | 0.00104 | Inflammation |
| Cytidine deaminase | P32320 | 0.054 | 0.011 | 8.61e-07 | 0.00106 | Cardiometabolic |
| Alpha-amylase 2B | P19961 | -0.044 | 0.009 | 1.07e-06 | 0.00131 | Cardiometabolic |
| Pantetheinase | O95497 | 0.028 | 0.006 | 1.21e-06 | 0.00149 | Inflammation |
| Pancreatic alpha-amylase | P04746 | -0.044 | 0.009 | 1.26e-06 | 0.00155 | Cardiometabolic |
| Uromodulin | P07911 | -0.039 | 0.008 | 1.49e-06 | 0.00183 | Cardiometabolic |
| BPI fold-containing family A member 2 | Q96DR5 | -0.02 | 0.004 | 1.82e-06 | 0.00225 | Cardiometabolic |
| B-cell differentiation antigen CD72 | P21854 | 0.049 | 0.01 | 2.63e-06 | 0.00324 | Inflammation |
| Egl nine homolog 1 | Q9GZT9 | 0.042 | 0.009 | 3.32e-06 | 0.00409 | Inflammation |
| Secretoglobin family 3A member 2 | Q96PL1 | -0.024 | 0.005 | 4.25e-06 | 0.00524 | Inflammation |
| Ficolin-2 | Q15485 | 0.049 | 0.01 | 4.63e-06 | 0.00571 | Cardiometabolic |
| Complement factor B | P00751 | 0.065 | 0.014 | 4.8e-06 | 0.00591 | Inflammation |
| C-C motif chemokine 27 | Q9Y4X3 | -0.041 | 0.009 | 4.86e-06 | 0.00598 | Cardiometabolic |
| Protein tyrosine phosphatase type IVA 3 | O75365 | 0.036 | 0.008 | 5.17e-06 | 0.00637 | Inflammation |
| Thimet oligopeptidase | P52888 | 0.064 | 0.014 | 5.39e-06 | 0.00664 | Cardiometabolic |
| D-dopachrome decarboxylase | P30046 | 0.046 | 0.01 | 5.89e-06 | 0.00726 | Cardiometabolic |
| Mevalonate kinase | Q03426 | 0.023 | 0.005 | 6.25e-06 | 0.0077 | Inflammation |
| Fatty acid-binding protein, heart | P05413 | 0.041 | 0.009 | 6.6e-06 | 0.00813 | Cardiometabolic |
| Collagen triple helix repeat-containing protein 1 | Q96CG8 | 0.061 | 0.013 | 7.06e-06 | 0.0087 | Cardiometabolic |
| Agrin | O00468 | 0.062 | 0.014 | 9.63e-06 | 0.0119 | Inflammation |
| Plasminogen activator inhibitor 1 | P05121 | 0.031 | 0.007 | 1e-05 | 0.0124 | Cardiometabolic |
| Tectonic-3 | Q6NUS6 | 0.056 | 0.013 | 1.02e-05 | 0.0126 | Cardiometabolic |
| Ganglioside GM2 activator | P17900 | 0.066 | 0.015 | 1.06e-05 | 0.0131 | Cardiometabolic |
| Delta and Notch-like epidermal growth factor-related receptor | Q8NFT8 | -0.073 | 0.016 | 1.11e-05 | 0.0137 | Inflammation |
| Cell surface hyaluronidase | Q9UHN6 | 0.059 | 0.013 | 1.17e-05 | 0.0144 | Inflammation |
| Receptor-type tyrosine-protein phosphatase zeta | P23471 | -0.053 | 0.012 | 1.18e-05 | 0.0145 | Cardiometabolic |
| Kynureninase | Q16719 | 0.043 | 0.01 | 1.2e-05 | 0.0148 | Inflammation |
| Interleukin-12 subunit beta | P29460 | 0.033 | 0.007 | 1.36e-05 | 0.0168 | Inflammation |
| Insulin-like growth factor-binding protein-like 1 | Q8WX77 | 0.062 | 0.014 | 1.47e-05 | 0.0181 | Cardiometabolic |
| Aflatoxin B1 aldehyde reductase member 4 | Q8NHP1 | 0.02 | 0.005 | 1.62e-05 | 0.02 | Inflammation |
| Serine--pyruvate aminotransferase | P21549 | 0.025 | 0.006 | 1.67e-05 | 0.0206 | Cardiometabolic |
| Stabilin-2 | Q8WWQ8 | 0.067 | 0.015 | 1.78e-05 | 0.0219 | Cardiometabolic |
| Multiple epidermal growth factor-like domains protein 9 | Q9H1U4 | 0.091 | 0.021 | 1.95e-05 | 0.024 | Cardiometabolic |
| Corticosteroid 11-beta-dehydrogenase isozyme 1 | P28845 | -0.055 | 0.013 | 2.01e-05 | 0.0247 | Inflammation |
| Leukocyte immunoglobulin-like receptor subfamily A member 5 | A6NI73 | 0.055 | 0.013 | 2.7e-05 | 0.0332 | Cardiometabolic |
| Secretogranin-3 | Q8WXD2 | -0.058 | 0.014 | 2.8e-05 | 0.0345 | Inflammation |
| Pseudokinase FAM20A | Q96MK3 | 0.051 | 0.012 | 2.93e-05 | 0.0361 | Cardiometabolic |
| Advanced glycosylation end product-specific receptor | Q15109 | -0.041 | 0.01 | 3e-05 | 0.037 | Inflammation |
| Angiopoietin-related protein 2 | Q9UKU9 | 0.042 | 0.01 | 3.02e-05 | 0.0372 | Inflammation |
| Interleukin-10 receptor subunit beta | Q08334 | 0.067 | 0.016 | 3.7e-05 | 0.0455 | Inflammation |
| Antithrombin-III | P01008 | -0.098 | 0.023 | 3.92e-05 | 0.0483 | Inflammation |
| Na(+)/H(+) exchange regulatory cofactor NHE-RF3 | Q5T2W1 | 0.044 | 0.006 | 8.24e-14 | 1.01e-10 | Inflammation |
| Sialoadhesin | Q9BZZ2 | 0.068 | 0.011 | 8.32e-10 | 1.02e-06 | Inflammation |
| Ketohexokinase | P50053 | 0.047 | 0.007 | 8.45e-10 | 1.04e-06 | Cardiometabolic |
| Leukocyte-associated immunoglobulin-like receptor 1 | Q6GTX8 | 0.082 | 0.012 | 8.41e-11 | 1.04e-07 | Inflammation |
| WAP, Kazal, immunoglobulin, Kunitz and NTR domain-containing protein 2 | Q8TEU8 | -0.072 | 0.012 | 8.64e-09 | 1.06e-05 | Inflammation |
| Carbonic anhydrase 5A, mitochondrial | P35218 | 0.025 | 0.004 | 9.02e-10 | 1.11e-06 | Cardiometabolic |
| Creatine kinase B-type | P12277 | -0.057 | 0.007 | 9.04e-16 | 1.11e-12 | Cardiometabolic |
| Proline-rich acidic protein 1 | Q96NZ9 | 0.079 | 0.009 | 9.55e-17 | 1.18e-13 | Cardiometabolic |
| Scavenger receptor cysteine-rich domain-containing group B protein | Q8WTU2 | 0.021 | 0.003 | 9.88e-11 | 1.22e-07 | Cardiometabolic |
| Bile salt sulfotransferase | Q06520 | 0.031 | 0.005 | 1.03e-08 | 1.27e-05 | Inflammation |
| Interleukin-18 receptor 1 | Q13478 | 0.081 | 0.011 | 1.03e-11 | 1.27e-08 | Inflammation |
| Insulin-like growth factor-binding protein 1 | P08833 | -0.041 | 0.004 | 1.05e-17 | 1.29e-14 | Cardiometabolic |
| BPI fold-containing family B member 2 | Q8N4F0 | 0.028 | 0.005 | 1.13e-08 | 1.39e-05 | Cardiometabolic |
| Galectin-9 | O00182 | 0.08 | 0.013 | 1.17e-09 | 1.44e-06 | Inflammation |
| Afamin | P43652 | 0.106 | 0.014 | 1.17e-13 | 1.44e-10 | Inflammation |
| Beta-glucuronidase | P08236 | 0.038 | 0.006 | 1.18e-10 | 1.46e-07 | Cardiometabolic |
| Interleukin-1 receptor antagonist protein | P18510 | 0.061 | 0.005 | 1.2e-25 | 1.48e-22 | Inflammation |
| Tumor necrosis factor receptor superfamily member 11A | Q9Y6Q6 | 0.069 | 0.01 | 1.23e-11 | 1.51e-08 | Inflammation |
| Complement factor H | P08603 | 0.131 | 0.018 | 1.24e-12 | 1.53e-09 | Inflammation |
| Galectin-3-binding protein | Q08380 | 0.065 | 0.009 | 1.29e-11 | 1.58e-08 | Inflammation |
| Scavenger receptor cysteine-rich type 1 protein M130 | Q86VB7 | 0.052 | 0.009 | 1.4e-08 | 1.73e-05 | Cardiometabolic |
| Pigment epithelium-derived factor | P36955 | 0.186 | 0.023 | 1.51e-14 | 1.86e-11 | Inflammation |
| Retinoic acid receptor responder protein 2 | Q99969 | 0.063 | 0.01 | 1.46e-09 | 1.8e-06 | Cardiometabolic |
| Collagen alpha-3(VI) chain | P12111 | 0.078 | 0.013 | 8.15e-09 | 1e-05 | Cardiometabolic |
| Leptin | P41159 | 0.052 | 0.003 | 1.66e-51 | 2.04e-48 | Cardiometabolic |
| Galectin-1 | P09382 | 0.086 | 0.014 | 1.84e-09 | 2.27e-06 | Cardiometabolic |
| Serum paraoxonase/lactonase 3 | Q15166 | -0.142 | 0.016 | 1.86e-16 | 2.29e-13 | Cardiometabolic |
| Spondin-2 | Q9BUD6 | 0.085 | 0.015 | 1.9e-08 | 2.34e-05 | Cardiometabolic |
| ADAMTS-like protein 2 | Q86TH1 | 0.073 | 0.011 | 1.91e-10 | 2.35e-07 | Cardiometabolic |
| Leukocyte immunoglobulin-like receptor subfamily B member 4 | Q8NHJ6 | 0.063 | 0.011 | 2.03e-08 | 2.5e-05 | Inflammation |
| Serum amyloid P-component | P02743 | 0.063 | 0.011 | 2.12e-08 | 2.62e-05 | Inflammation |
| Growth/differentiation factor 15 | O43184 | 0.069 | 0.009 | 2.18e-13 | 2.68e-10 | Inflammation |
| Prostaglandin reductase 1 | Q14914 | 0.032 | 0.005 | 2.29e-11 | 2.82e-08 | Cardiometabolic |
| Ribonuclease pancreatic | P07998 | 0.119 | 0.016 | 2.32e-12 | 2.86e-09 | Inflammation |
| Insulin-like growth factor-binding protein 2 | P18065 | -0.057 | 0.006 | 2.47e-17 | 3.04e-14 | Cardiometabolic |
| Glutathione S-transferase A1 | P08263 | 0.031 | 0.003 | 2.51e-17 | 3.09e-14 | Cardiometabolic |
| All-trans-retinol dehydrogenase [NAD(+)] ADH4 | P08319 | 0.036 | 0.005 | 2.59e-12 | 3.19e-09 | Cardiometabolic |
| 2-iminobutanoate/2-iminopropanoate deaminase | P52758 | 0.051 | 0.006 | 2.59e-15 | 3.19e-12 | Inflammation |
| Zinc transporter ZIP5 | Q6ZMH5 | 0.043 | 0.007 | 2.64e-09 | 3.26e-06 | Inflammation |
| Matrix remodeling-associated protein 8 | Q9BRK3 | -0.072 | 0.013 | 2.66e-08 | 3.27e-05 | Inflammation |
| Growth hormone receptor | P10912 | 0.091 | 0.011 | 3.06e-14 | 3.77e-11 | Inflammation |
| Cathepsin D | P07339 | 0.075 | 0.01 | 3.09e-13 | 3.81e-10 | Cardiometabolic |
| Liver carboxylesterase 1 | P23141 | 0.035 | 0.005 | 3.15e-12 | 3.88e-09 | Cardiometabolic |
| All-trans-retinol dehydrogenase | P00325 | 0.033 | 0.004 | 3.37e-12 | 4.15e-09 | Inflammation |
| Apolipoprotein D | P05090 | -0.072 | 0.012 | 3.39e-09 | 4.18e-06 | Inflammation |
| Beta-ureidopropionase | Q9UBR1 | 0.025 | 0.004 | 3.57e-10 | 4.39e-07 | Cardiometabolic |
| Protein turtle homolog A | Q9P2J2 | 0.036 | 0.004 | 3.59e-18 | 4.42e-15 | Cardiometabolic |
| NHL repeat-containing protein 3 | Q5JS37 | 0.11 | 0.016 | 3.62e-11 | 4.46e-08 | Inflammation |
| Sex hormone-binding globulin | P04278 | -0.053 | 0.008 | 3.79e-11 | 4.67e-08 | Inflammation |
| Low-density lipoprotein receptor | P01130 | 0.048 | 0.009 | 3.88e-08 | 4.78e-05 | Cardiometabolic |
| Protein disulfide isomerase CRELD1 | Q96HD1 | 0.095 | 0.014 | 3.92e-11 | 4.82e-08 | Inflammation |
| Cadherin-2 | P19022 | 0.068 | 0.012 | 4.36e-08 | 5.37e-05 | Cardiometabolic |
| Complement C3 | P01024 | 0.071 | 0.01 | 4.48e-11 | 5.52e-08 | Inflammation |
| Apolipoprotein F | Q13790 | -0.148 | 0.02 | 4.77e-13 | 5.88e-10 | Inflammation |
| E-selectin | P16581 | 0.042 | 0.008 | 5.13e-08 | 6.32e-05 | Cardiometabolic |
| Pterin-4-alpha-carbinolamine dehydratase | P61457 | 0.047 | 0.007 | 5.16e-11 | 6.36e-08 | Inflammation |
| High affinity immunoglobulin alpha and immunoglobulin mu Fc receptor | Q8WWV6 | 0.042 | 0.006 | 5.2e-13 | 6.4e-10 | Cardiometabolic |
| Sialic acid-binding Ig-like lectin 7 | Q9Y286 | 0.086 | 0.014 | 5.7e-09 | 7.03e-06 | Cardiometabolic |
| Hepatocyte growth factor | P14210 | 0.07 | 0.01 | 5.92e-12 | 7.29e-09 | Inflammation |
| Fatty acid-binding protein, liver | P07148 | 0.027 | 0.004 | 5.96e-09 | 7.34e-06 | Inflammation |
| Complement factor I | P05156 | 0.152 | 0.025 | 6.06e-09 | 7.47e-06 | Inflammation |
| Phospholipase A2 | P04054 | -0.048 | 0.009 | 6.07e-08 | 7.48e-05 | Cardiometabolic |
| Follistatin-related protein 3 | O95633 | 0.081 | 0.013 | 6.33e-10 | 7.8e-07 | Inflammation |
| CD59 glycoprotein | P13987 | 0.12 | 0.02 | 6.41e-09 | 7.9e-06 | Cardiometabolic |
| Fatty acid-binding protein, adipocyte | P15090 | 0.073 | 0.007 | 6.97e-23 | 8.59e-20 | Cardiometabolic |
| Basement membrane-specific heparan sulfate proteoglycan core protein | P98160 | 0.112 | 0.018 | 7.27e-10 | 8.95e-07 | Cardiometabolic |
| Microfibrillar-associated protein 5 | Q13361 | 0.069 | 0.012 | 7.78e-09 | 9.59e-06 | Cardiometabolic |
| Aminoacylase-1 | Q03154 | 0.052 | 0.007 | 7.83e-14 | 9.64e-11 | Cardiometabolic |
| Semaphorin-3F | Q13275 | 0.106 | 0.018 | 8.07e-09 | 9.94e-06 | Cardiometabolic |

**Caption:** Only significant associations are displayed. LME models were as follow: Change_BMI ~ Sex + Zygosity + Age at blood sample + Protein + BMI_baseline + (1|Family ID). **Abbreviations:** SE: Standard error.

**Supplementary table 4.** Significant biological pathways of the proteins at ~62 years old significantly associated with BMI changes during adulthood (24 to 62 years old).

| Pathway identifier | Pathway name | Entities found | Entities FDR |
| --- | --- | --- | --- |
| R-HSA-381426 | Regulation of Insulin-like Growth Factor (IGF) transport and uptake by Insulin-like Growth Factor Binding Proteins (IGFBPs) | 12 | 1,1818729414136975E-5 |
| R-HSA-8957275 | Post-translational protein phosphorylation | 11 | 1,252673862572884E-5 |
| R-HSA-173736 | Alternative complement activation | 3 | 0,004228706119397407 |
| R-HSA-449836 | Other interleukin signaling | 4 | 0,019852023965578613 |

**Caption:** The names of the biological pathways of the proteins at ~62 years old previously found to be significantly associated with changes in BMI during adulthood are displayed along with the pathway reference, the number of proteins belonging to the pathway and the p-value for that pathway. **Abbreviations:** FDR: False discovery rate; BMI: Body mass index.

**Supplementary table** **5.** Linear mixed-effects models to assess associations of proteins at ~62 years old with BMI changes during adulthood (from 24 to 62 years old) including only individuals with BMI less than 30 kg/m^2^.

|  |  |  |  | **p value** | | **Protein** |
| --- | --- | --- | --- | --- | --- | --- |
| **Proteindescription** | **Protein ID** | **Estimate** | **SE** | **Nominal** | **Bonferroni** | **panel** |
| Leptin | P41159 | 0,039841 | 0,002664 | 2,36E-35 | 2,90E-32 | Cardiometabolic |
| Fatty acid-binding protein, adipocyte | P15090 | 0,048604 | 0,00645 | 1,14E-12 | 1,40E-09 | Cardiometabolic |
| Interleukin-1 receptor antagonist protein | P18510 | 0,041266 | 0,005556 | 2,29E-12 | 2,82E-09 | Inflammation |
| Insulin-like growth factor-binding protein 2 | P18065 | -0,04031 | 0,00579 | 3,52E-11 | 4,34E-08 | Cardiometabolic |
| Insulin-like growth factor-binding protein 1 | P08833 | -0,02827 | 0,004105 | 5,55E-11 | 6,84E-08 | Cardiometabolic |
| Creatine kinase B-type | P12277 | -0,04053 | 0,005983 | 1,07E-10 | 1,31E-07 | Cardiometabolic |
| Sex hormone-binding globulin | P04278 | -0,04146 | 0,006639 | 2,11E-09 | 2,60E-06 | Inflammation |
| Proline-rich acidic protein 1 | Q96NZ9 | 0,047757 | 0,00807 | 1,20E-08 | 1,48E-05 | Cardiometabolic |
| Growth hormone receptor | P10912 | 0,058409 | 0,009905 | 1,35E-08 | 1,67E-05 | Inflammation |
| Serum paraoxonase/lactonase 3 | Q15166 | -0,0963 | 0,017241 | 6,83E-08 | 8,41E-05 | Cardiometabolic |
| Tissue-type plasminogen activator | P00750 | 0,027459 | 0,005075 | 1,60E-07 | 0,000197 | Cardiometabolic |
| Protein turtle homolog A | Q9P2J2 | 0,019812 | 0,003696 | 2,10E-07 | 0,000259 | Cardiometabolic |
| Phospholipid transfer protein | P55058 | -0,05719 | 0,010869 | 3,49E-07 | 0,00043 | Cardiometabolic |
| Pigment epithelium-derived factor | P36955 | 0,110446 | 0,021757 | 8,04E-07 | 0,00099 | Inflammation |
| Disintegrin and metalloproteinase domain-containing protein 12 | O43184 | 0,043626 | 0,008622 | 8,71E-07 | 0,001073 | Inflammation |
| 2-iminobutanoate/2-iminopropanoate deaminase | P52758 | 0,03051 | 0,006062 | 1,06E-06 | 0,001305 | Inflammation |
| Golgi-associated kinase 1A | Q9UFP1 | 0,036852 | 0,007546 | 1,96E-06 | 0,002418 | Cardiometabolic |
| Galectin-9 | O00182 | 0,054034 | 0,011177 | 2,46E-06 | 0,003032 | Inflammation |
| Scavenger receptor cysteine-rich domain-containing group B protein | Q8WTU2 | 0,013885 | 0,002893 | 2,96E-06 | 0,003643 | Cardiometabolic |
| Carbonic anhydrase 5A, mitochondrial | P35218 | 0,016813 | 0,003557 | 4,03E-06 | 0,004963 | Cardiometabolic |
| Zinc transporter ZIP5 | Q6ZMH5 | 0,029943 | 0,006349 | 4,19E-06 | 0,005162 | Inflammation |
| Glutathione S-transferase A1 | P08263 | 0,016872 | 0,003579 | 4,37E-06 | 0,005383 | Cardiometabolic |
| WAP, Kazal, immunoglobulin, Kunitz and NTR domain-containing protein 2 | Q8TEU8 | -0,04882 | 0,010392 | 4,93E-06 | 0,006071 | Inflammation |
| Low-density lipoprotein receptor | P01130 | 0,034603 | 0,007534 | 7,24E-06 | 0,008914 | Cardiometabolic |
| CD59 glycoprotein | P13987 | 0,08474 | 0,018654 | 9,05E-06 | 0,011153 | Cardiometabolic |
| Apolipoprotein D | P05090 | -0,04685 | 0,010295 | 9,11E-06 | 0,011228 | Inflammation |
| Semaphorin-3F | Q13275 | 0,069431 | 0,015304 | 9,33E-06 | 0,011498 | Cardiometabolic |
| NHL repeat-containing protein 3 | Q5JS37 | 0,067235 | 0,015 | 1,18E-05 | 0,014526 | Inflammation |
| Ribonuclease pancreatic | P07998 | 0,067757 | 0,015307 | 1,49E-05 | 0,018332 | Inflammation |
| Uromodulin | P07911 | -0,0319 | 0,007257 | 1,77E-05 | 0,021796 | Cardiometabolic |
| Neurexophilin-3 | O95157 | -0,04645 | 0,010585 | 1,79E-05 | 0,022055 | Inflammation |
| Interleukin-18 receptor 1 | Q13478 | 0,045494 | 0,010426 | 1,94E-05 | 0,023916 | Inflammation |
| Leukocyte immunoglobulin-like receptor subfamily B member 4 | Q8NHJ6 | 0,04071 | 0,009366 | 2,11E-05 | 0,025985 | Inflammation |
| Serum amyloid P-component | P02743 | 0,040887 | 0,009419 | 2,14E-05 | 0,026326 | Inflammation |
| SLIT and NTRK-like protein 1 | Q96PX8 | -0,04738 | 0,010921 | 2,18E-05 | 0,026856 | Inflammation |
| Secretoglobin family 3A member 1 | Q96QR1 | -0,04407 | 0,01018 | 2,25E-05 | 0,027674 | Inflammation |
| Phospholipase A2 | P04054 | -0,03207 | 0,007531 | 3,01E-05 | 0,037138 | Cardiometabolic |
| Afamin | P43652 | 0,054438 | 0,01281 | 3,12E-05 | 0,038437 | Inflammation |
| Complement C3 | P01024 | 0,039021 | 0,009283 | 3,88E-05 | 0,04777 | Inflammation |
| Sialoadhesin | Q9BZZ2 | 0,042594 | 0,010167 | 4,01E-05 | 0,049401 | Inflammation |

**Caption:** significant association results displayed from the use of LME model: Change_BMI ~ Sex + Zygosity + Age at blood sample + Protein + BMI_baseline + (1|Family ID) after removing samples with BMI>30 kg/m2. **Abbreviations:** SE: Standard error.

**Supplementary table 6.** Linear mixed-effects models to assess associations of proteins at ~62 years old with BMI changes during adulthood (from 24 to 62 years old) including the intake of anti-hypertensive medication, physical activity and diet as a covariate.

| **Protein name** | **Protein** |  |  | **p value** | | **Protein**  **Panel** |
| --- | --- | --- | --- | --- | --- | --- |
|  | **ID** | **Estimate** | **SE** | **Nominal** | **Bonferroni** |  |
| Pigment epithelium-derived factor | P36955 | 0,175126 | 0,022766 | 2,30E-13 | 2,83E-10 | Inflammation |
| Complement factor I | P05156 | 0,147101 | 0,025046 | 1,18E-08 | 1,45E-05 | Inflammation |
| Apolipoprotein F | Q13790 | -0,139492 | 0,019080 | 3,05E-12 | 3,76E-09 | Inflammation |
| Serum paraoxonase/lactonase 3 | Q15166 | -0,132317 | 0,016278 | 1,77E-14 | 2,17E-11 | Inflammation |
| Complement factor H | P08603 | 0,121555 | 0,017579 | 2,97E-11 | 3,66E-08 | Inflammation |
| CD59 glycoprotein | P13987 | 0,117299 | 0,020011 | 1,24E-08 | 1,53E-05 | Cardiometabolic |
| Ribonuclease pancreatic | P07998 | 0,114954 | 0,016196 | 9,82E-12 | 1,21E-08 | Inflammation |
| Semaphorin-3F | Q13275 | 0,109858 | 0,017671 | 1,76E-09 | 2,17E-06 | Cardiometabolic |
| Basement membrane-specific heparan sulfate proteoglycan core protein | P98160 | 0,102668 | 0,017659 | 1,60E-08 | 1,97E-05 | Cardiometabolic |
| Coagulation factor IX | P00740 | 0,100877 | 0,019666 | 5,33E-07 | 6,57E-04 | Cardiometabolic |
| NHL repeat-containing protein 3 | Q5JS37 | 0,099939 | 0,016000 | 1,50E-09 | 1,85E-06 | Inflammation |
| Afamin | P43652 | 0,098124 | 0,013755 | 7,89E-12 | 9,71E-09 | Inflammation |
| Complement factor D | P00746 | 0,093646 | 0,020527 | 7,51E-06 | 9,24E-03 | Inflammation |
| Growth hormone receptor | P10912 | 0,090457 | 0,011338 | 3,49E-14 | 4,30E-11 | Inflammation |
| Macrophage colony-stimulating factor 1 | P09603 | 0,089385 | 0,016782 | 2,04E-07 | 2,51E-04 | Inflammation |
| Protein disulfide isomerase CRELD1 | Q96HD1 | 0,089221 | 0,013677 | 3,12E-10 | 3,84E-07 | Inflammation |
| Collectin-12 | Q5KU26 | 0,087923 | 0,018341 | 2,62E-06 | 3,23E-03 | Inflammation |
| Galectin-1 | P09382 | 0,082016 | 0,013762 | 7,30E-09 | 8,99E-06 | Cardiometabolic |
| Sialic acid-binding Ig-like lectin 7 | Q9Y286 | 0,080960 | 0,014533 | 5,78E-08 | 7,11E-05 | Cardiometabolic |
| Dermatopontin | Q07507 | 0,080638 | 0,016133 | 1,01E-06 | 1,24E-03 | Cardiometabolic |
| Leukocyte-associated immunoglobulin-like receptor 1 | Q6GTX8 | 0,079259 | 0,012526 | 1,01E-09 | 1,24E-06 | Inflammation |
| Spondin-2 | Q9BUD6 | 0,078824 | 0,014447 | 1,05E-07 | 1,29E-04 | Cardiometabolic |
| Follistatin-related protein 3 | O95633 | 0,077231 | 0,012655 | 3,33E-09 | 4,10E-06 | Inflammation |
| Collagen alpha-3(VI) chain | P12111 | 0,075286 | 0,013193 | 2,88E-08 | 3,54E-05 | Cardiometabolic |
| Interleukin-18 receptor 1 | Q13478 | 0,074749 | 0,011431 | 2,78E-10 | 3,42E-07 | Inflammation |
| Proline-rich acidic protein 1 | Q96NZ9 | 0,073373 | 0,009077 | 1,75E-14 | 2,15E-11 | Cardiometabolic |
| Galectin-9 | O00182 | 0,072739 | 0,012793 | 3,17E-08 | 3,90E-05 | Inflammation |
| Fatty acid-binding protein, adipocyte | P15090 | 0,072610 | 0,007021 | 1,52E-21 | 1,87E-18 | Cardiometabolic |
| Lysosomal Pro-X carboxypeptidase | P42785 | 0,072095 | 0,012920 | 5,50E-08 | 6,77E-05 | Cardiometabolic |
| Leptin receptor | P48357 | -0,071957 | 0,015685 | 6,96E-06 | 8,57E-03 | Cardiometabolic |
| Cathepsin D | P07339 | 0,071032 | 0,009758 | 3,35E-12 | 4,13E-09 | Cardiometabolic |
| WAP, Kazal, immunoglobulin, Kunitz and NTR domain-containing protein 2 | Q8TEU8 | -0,070360 | 0,012088 | 1,77E-08 | 2,18E-05 | Inflammation |
| Stabilin-2 | Q8WWQ8 | 0,069183 | 0,015201 | 7,94E-06 | 9,77E-03 | Cardiometabolic |
| Platelet glycoprotein 4 | P16671 | 0,068867 | 0,013231 | 3,67E-07 | 4,52E-04 | Inflammation |
| Apolipoprotein D | P05090 | -0,068578 | 0,011876 | 2,07E-08 | 2,55E-05 | Inflammation |
| Interleukin-10 receptor subunit beta | Q08334 | 0,068447 | 0,015608 | 1,63E-05 | 2,00E-02 | Inflammation |
| Ganglioside GM2 activator | P17900 | 0,066838 | 0,014678 | 7,86E-06 | 9,67E-03 | Cardiometabolic |
| Hepatocyte growth factor | P14210 | 0,066525 | 0,009520 | 2,01E-11 | 2,48E-08 | Inflammation |
| Sialoadhesin | Q9BZZ2 | 0,066207 | 0,010872 | 3,67E-09 | 4,52E-06 | Inflammation |
| Dickkopf-related protein 3 | Q9UBP4 | -0,065399 | 0,012786 | 5,97E-07 | 7,35E-04 | Cardiometabolic |
| Complement C3 | P01024 | 0,065304 | 0,010398 | 1,30E-09 | 1,61E-06 | Inflammation |
| Tumor necrosis factor receptor superfamily member 11A | Q9Y6Q6 | 0,064870 | 0,009882 | 2,39E-10 | 2,94E-07 | Inflammation |
| Microfibrillar-associated protein 5 | Q13361 | 0,064169 | 0,011641 | 7,81E-08 | 9,62E-05 | Cardiometabolic |
| Serum amyloid P-component | P02743 | 0,063986 | 0,010726 | 7,16E-09 | 8,81E-06 | Inflammation |
| Disintegrin and metalloproteinase domain-containing protein 12 | O43184 | 0,063949 | 0,008894 | 5,89E-12 | 7,25E-09 | Inflammation |
| Neurexophilin-3 | O95157 | -0,063750 | 0,012802 | 1,10E-06 | 1,36E-03 | Inflammation |
| ADAMTS-like protein 2 | Q86TH1 | 0,063171 | 0,011233 | 4,49E-08 | 5,53E-05 | Cardiometabolic |
| Matrix remodeling-associated protein 8 | Q9BRK3 | -0,062963 | 0,012827 | 1,55E-06 | 1,91E-03 | Inflammation |
| Collagen triple helix repeat-containing protein 1 | Q96CG8 | 0,062058 | 0,013432 | 5,77E-06 | 7,10E-03 | Cardiometabolic |
| Cadherin-2 | P19022 | 0,061798 | 0,011990 | 4,71E-07 | 5,80E-04 | Cardiometabolic |
| Gamma-glutamyl hydrolase | Q92820 | 0,061276 | 0,011025 | 6,38E-08 | 7,85E-05 | Cardiometabolic |
| Phospholipid transfer protein | P55058 | -0,061009 | 0,012336 | 1,34E-06 | 1,66E-03 | Cardiometabolic |
| Galectin-3-binding protein | Q08380 | 0,060530 | 0,009198 | 2,18E-10 | 2,68E-07 | Inflammation |
| SLIT and NTRK-like protein 1 | Q96PX8 | -0,060131 | 0,013107 | 6,70E-06 | 8,25E-03 | Inflammation |
| Complement factor B | P00751 | 0,059784 | 0,013771 | 1,96E-05 | 2,41E-02 | Inflammation |
| Cell surface hyaluronidase | Q9UHN6 | 0,059241 | 0,012857 | 6,19E-06 | 7,62E-03 | Inflammation |
| Thimet oligopeptidase | P52888 | 0,058482 | 0,013823 | 3,12E-05 | 3,84E-02 | Cardiometabolic |
| Retinoic acid receptor responder protein 2 | Q99969 | 0,057991 | 0,010073 | 2,19E-08 | 2,70E-05 | Cardiometabolic |
| Interleukin-1 receptor antagonist protein | P18510 | 0,057469 | 0,005353 | 8,71E-23 | 1,07E-19 | Inflammation |
| Glycerophosphocholine cholinephosphodiesterase ENPP6 | Q6UWR7 | -0,056609 | 0,011090 | 6,00E-07 | 7,39E-04 | Cardiometabolic |
| Leukocyte immunoglobulin-like receptor subfamily B member 4 | Q8NHJ6 | 0,055102 | 0,010970 | 9,03E-07 | 1,11E-03 | Inflammation |
| Leukocyte immunoglobulin-like receptor subfamily A member 5 | A6NI73 | 0,054244 | 0,012862 | 3,34E-05 | 4,11E-02 | Cardiometabolic |
| Insulin-like growth factor-binding protein 2 | P18065 | -0,053540 | 0,006303 | 1,16E-15 | 1,43E-12 | Cardiometabolic |
| Creatine kinase B-type | P12277 | -0,053386 | 0,006697 | 3,70E-14 | 4,56E-11 | Cardiometabolic |
| Secreted frizzled-related protein 4 | Q6FHJ7 | 0,053337 | 0,012336 | 2,11E-05 | 2,60E-02 | Inflammation |
| Cytidine deaminase | P32320 | 0,053299 | 0,010577 | 8,28E-07 | 1,02E-03 | Cardiometabolic |
| Corticosteroid 11-beta-dehydrogenase isozyme 1 | P28845 | -0,052866 | 0,012398 | 2,75E-05 | 3,38E-02 | Inflammation |
| Leptin | P41159 | 0,051247 | 0,002864 | 3,34E-48 | 4,12E-45 | Cardiometabolic |
| Scavenger receptor cysteine-rich type 1 protein M130 | Q86VB7 | 0,051112 | 0,008926 | 2,60E-08 | 3,20E-05 | Cardiometabolic |
| Sex hormone-binding globulin | P04278 | -0,049880 | 0,007770 | 5,55E-10 | 6,84E-07 | Inflammation |
| B-cell differentiation antigen CD72 | P21854 | 0,047344 | 0,010208 | 5,32E-06 | 6,55E-03 | Inflammation |
| Thrombospondin-4 | P35443 | 0,047094 | 0,008311 | 3,56E-08 | 4,38E-05 | Cardiometabolic |
| Aminoacylase-1 | Q03154 | 0,046055 | 0,007026 | 2,54E-10 | 3,13E-07 | Cardiometabolic |
| Ficolin-2 | Q15485 | 0,045906 | 0,010425 | 1,56E-05 | 1,92E-02 | Cardiometabolic |
| Phospholipase A2 | P04054 | -0,045212 | 0,008681 | 3,61E-07 | 4,45E-04 | Cardiometabolic |
| 2-iminobutanoate/2-iminopropanoate deaminase | P52758 | 0,045054 | 0,006326 | 9,49E-12 | 1,17E-08 | Inflammation |
| Macrophage colony-stimulating factor 1 receptor | P07333 | 0,044883 | 0,009150 | 1,56E-06 | 1,91E-03 | Inflammation |
| Low-density lipoprotein receptor | P01130 | 0,044214 | 0,008505 | 3,84E-07 | 4,72E-04 | Cardiometabolic |
| Angiomotin | Q4VCS5 | 0,043993 | 0,008888 | 1,26E-06 | 1,55E-03 | Inflammation |
| C-C motif chemokine 16 | O15467 | 0,043968 | 0,009799 | 1,05E-05 | 1,29E-02 | Cardiometabolic |
| N-acetylneuraminate lyase | Q9BXD5 | 0,043891 | 0,009077 | 2,18E-06 | 2,68E-03 | Cardiometabolic |
| C-C motif chemokine 27 | Q9Y4X3 | -0,043606 | 0,008724 | 1,05E-06 | 1,29E-03 | Cardiometabolic |
| Alpha-amylase 1A_Alpha-amylase 1B_Alpha-amylase 1C | P0DUB6_P0DTE7_P0DTE8 | -0,042376 | 0,009402 | 9,64E-06 | 1,19E-02 | Inflammation |
| Ketohexokinase | P50053 | 0,041101 | 0,007409 | 6,56E-08 | 8,07E-05 | Cardiometabolic |
| Pterin-4-alpha-carbinolamine dehydratase | P61457 | 0,041071 | 0,007111 | 2,07E-08 | 2,55E-05 | Inflammation |
| Pancreatic alpha-amylase | P04746 | -0,040920 | 0,009084 | 9,79E-06 | 1,21E-02 | Cardiometabolic |
| High affinity immunoglobulin alpha and immunoglobulin mu Fc receptor | Q8WWV6 | 0,040917 | 0,005602 | 2,71E-12 | 3,33E-09 | Cardiometabolic |
| Insulin-like growth factor-binding protein 1 | P08833 | -0,040789 | 0,004377 | 3,23E-18 | 3,97E-15 | Cardiometabolic |
| Alpha-amylase 2B | P19961 | -0,040526 | 0,008940 | 8,67E-06 | 1,07E-02 | Cardiometabolic |
| E-selectin | P16581 | 0,039265 | 0,007474 | 2,90E-07 | 3,57E-04 | Cardiometabolic |
| Na(+)/H(+) exchange regulatory cofactor NHE-RF3 | Q5T2W1 | 0,038678 | 0,005820 | 1,57E-10 | 1,94E-07 | Inflammation |
| Zinc transporter ZIP5 | Q6ZMH5 | 0,037792 | 0,007165 | 2,60E-07 | 3,20E-04 | Inflammation |
| Beta-glucuronidase | P08236 | 0,036563 | 0,005579 | 2,55E-10 | 3,14E-07 | Cardiometabolic |
| Angiotensin-converting enzyme 2 | Q9BYF1 | 0,033677 | 0,007519 | 1,08E-05 | 1,33E-02 | Cardiometabolic |
| Protein turtle homolog A | Q9P2J2 | 0,033542 | 0,003999 | 2,18E-15 | 2,68E-12 | Cardiometabolic |
| Chordin-like protein 2 | Q6WN34 | 0,033407 | 0,007584 | 1,54E-05 | 1,89E-02 | Cardiometabolic |
| Liver carboxylesterase 1 | P23141 | 0,031910 | 0,004774 | 1,19E-10 | 1,46E-07 | Cardiometabolic |
| All-trans-retinol dehydrogenase [NAD(+)] ADH4 | P08319 | 0,031739 | 0,005123 | 2,05E-09 | 2,52E-06 | Cardiometabolic |
| BPI fold-containing family B member 1 | Q8TDL5 | -0,031331 | 0,006284 | 1,08E-06 | 1,33E-03 | Cardiometabolic |
| BPI fold-containing family B member 2 | Q8N4F0 | 0,028983 | 0,004655 | 1,69E-09 | 2,07E-06 | Cardiometabolic |
| Plasminogen activator inhibitor 1 | P05121 | 0,028721 | 0,006873 | 3,89E-05 | 4,79E-02 | Cardiometabolic |
| Prostaglandin reductase 1 | Q14914 | 0,028644 | 0,004677 | 2,96E-09 | 3,64E-06 | Cardiometabolic |
| Glutathione S-transferase A1 | P08263 | 0,028474 | 0,003680 | 1,75E-13 | 2,16E-10 | Cardiometabolic |
| All-trans-retinol dehydrogenase | P00325 | 0,028098 | 0,004677 | 5,85E-09 | 7,20E-06 | Inflammation |
| Pantetheinase | O95497 | 0,026785 | 0,005544 | 2,36E-06 | 2,90E-03 | Inflammation |
| Bile salt sulfotransferase | Q06520 | 0,025436 | 0,005350 | 3,18E-06 | 3,92E-03 | Inflammation |
| Fructose-1,6-bisphosphatase 1 | P09467 | 0,022932 | 0,005174 | 1,36E-05 | 1,67E-02 | Cardiometabolic |
| Ectonucleotide pyrophosphatase/phosphodiesterase family member 7 | Q6UWV6 | 0,022721 | 0,004290 | 2,44E-07 | 3,00E-04 | Inflammation |
| Secretoglobin family 3A member 2 | Q96PL1 | -0,022713 | 0,005036 | 9,44E-06 | 1,16E-02 | Inflammation |
| Fatty acid-binding protein, liver | P07148 | 0,022530 | 0,004624 | 1,85E-06 | 2,28E-03 | Inflammation |
| Beta-ureidopropionase | Q9UBR1 | 0,021549 | 0,003978 | 1,29E-07 | 1,59E-04 | Cardiometabolic |
| Scavenger receptor cysteine-rich domain-containing group B protein | Q8WTU2 | 0,021286 | 0,003117 | 5,20E-11 | 6,40E-08 | Cardiometabolic |
| Carbonic anhydrase 5A, mitochondrial | P35218 | 0,021272 | 0,003914 | 1,18E-07 | 1,45E-04 | Cardiometabolic |
| Interleukin-6 | P05231 | 0,020108 | 0,004634 | 1,98E-05 | 2,44E-02 | Cardiometabolic |
| Somatotropin | P01241 | -0,012284 | 0,002305 | 2,01E-07 | 2,47E-04 | Cardiometabolic |

**Caption:** significant association results displayed from the use of LME model: Change_BMI ~ Sex + Zygosity + Age at blood sample + Protein + BMI_baseline + Intake of Anti-hypertensive medicament + Physical activity + Diet + (1|Family ID) after adding as a confounder the intake of anti-hypertensive medicaments. **Abbreviations:** SE: Standard error.

**Supplementary table 7.** Significant biological pathways of the proteins at ~62 years old significantly associated with BMI fluctuations during adulthood (24 to 62 years old).

| **Pathway identifier** | **Pathway name** | **Entities found** | **Entities FDR** |
| --- | --- | --- | --- |
| R-HSA-381340 | Transcriptional regulation of white adipocyte differentiation | 3 | 0,01898849876892761 |
| R-HSA-9843745 | Adipogenesis | 3 | 0,021138654209823304 |

**Caption:** The names of the biological pathways of the proteins at ~62 years old previously found to be significantly associated with fluctuations in BMI during adulthood are displayed along with the pathway reference, the number of proteins belonging to the pathway and the p-value for that pathway. **Abbreviations:** FDR: False discovery rate; BMI: Body mass index.

**Supplementary table 8.** Linear mixed-effects models to assess associations of BMI fluctuation and proteins at ~62 years old including BMI slope and BMI intercept as covariates.

| **Protein Description** | **Protein ID** | **Estimate** | **SE** | **p value** | | **Protein panel** |
| --- | --- | --- | --- | --- | --- | --- |
|  |  |  |  | **Nominal** | **Bonferroni** |  |
| Leptin receptor | P48357 | 0,76 | 0,34 | 0,03 | 1 | Cardiometabolic |
| Interleukin-1 receptor antagonist protein | P18510 | 0,35 | 0,15 | 0,02 | 1 | Inflammation |
| Fatty acid-binding protein, adipocyte | P15090 | 0,31 | 0,19 | 0,1 | 1 | Cardiometabolic |
| Angiopoietin-related protein 4 | Q9BY76 | 0,7 | 0,23 | 2,10E-03 | 1 | Inflammation |
| Disintegrin and metalloproteinase domain-containing protein 12 | O43184 | 0,43 | 0,21 | 0,04 | 1 | Inflammation |
| Na(+)/H(+) exchange regulatory cofactor NHE-RF3 | Q5T2W1 | 0,36 | 0,13 | 6,50E-03 | 1 | Inflammation |
| Growth/differentiation factor 15 | Q99988 | 0,59 | 0,22 | 7,00E-03 | 1 | Inflammation |
| Bile salt sulfotransferase | Q06520 | 0,34 | 0,11 | 2,80E-03 | 1 | Inflammation |
| 2-iminobutanoate/2-iminopropanoate deaminase | P52758 | 0,35 | 0,15 | 0,02 | 1 | Inflammation |
| Coiled-coil domain-containing protein 80 | Q76M96 | 0,82 | 0,21 | 1,23E-04 | 0,15 | Cardiometabolic |
| Interleukin-10 receptor subunit beta | Q08334 | 0,97 | 0,33 | 3,21E-03 | 1 | Inflammation |
| Aflatoxin B1 aldehyde reductase member 4 | Q8NHP1 | 0,29 | 0,1 | 2,94E-03 | 1 | Inflammation |
| Pterin-4-alpha-carbinolamine dehydratase | P61457 | 0,41 | 0,16 | 0,01 | 1 | Inflammation |
| Scavenger receptor cysteine-rich type 1 protein M130 | Q86VB7 | 0,54 | 0,19 | 5,26E-03 | 1 | Cardiometabolic |
| Pantetheinase | O95497 | 0,31 | 0,12 | 9,44E-03 | 1 | Inflammation |
| Glutathione S-transferase A1 | P08263 | 0,15 | 0,09 | 0,08 | 1 | Cardiometabolic |
| E-selectin | P16581 | 0,34 | 0,16 | 0,04 | 1 | Cardiometabolic |

**Caption:** significant association results displayed from the use of LME model: BMI_fluctuation ~ Sex + Zygosity + Age at blood sample + Protein + (1|Family ID) after adding BMI baseline and changes in BMI during adulthood (i.e. slope) as a fixed effect as part of sensitivity test. **Abbreviations:** SE: Standard error.

**Supplementary table 9.** Linear mixed-effects models to assess associations of BMI changes and fluctuations with polygenic risk score (PRS) for BMI.

| **Variables comparison** | **Estimate** | **SE** | **p value** | **95%CI** | |
| --- | --- | --- | --- | --- | --- |
|  |  |  |  | **LB** | **UB** |
| BMI changes – PRS_BMI_ | 0,01 | 4,80E-03 | 0,01 | 2,00E-03 | 0,02 |
| BMI fluctuations – PRS_BMI_ | -0,09 | 0,09 | 0,33 | -0,22 | 0,35 |

**Caption:** Significant association results displayed from the use of LME model: Change_BMI (or BMI_fluctuation) ~ Sex + Zygosity + Age at blood sample + PRS_BMI_ + BMI_baseline + (1|Family ID) where changes in BMI is the outcome and the protein level at ~62 years old, sex, the age of blood sampling, the baseline BMI (~24 years old) and the Polygenic risk score were fixed effects. **Abbreviations:** SE: Standard error; LB: Lower bound; UB: Upper bound.

**Supplementary table 10.** Within-pair analysis assessing which of the previously identified associations between proteins and BMI changes during adulthood remained significant when controlling for all genetic confounding.

| **Protein Description** | **Protein ID** | **Estimate** | **SE** | **p value** | | **Protein**  **panel** |
| --- | --- | --- | --- | --- | --- | --- |
|  |  |  |  | **Nominal** | **Bonferroni** |  |
| Leptin | P41159 | 0,05 | 0,007991747 | 7,78896E-07 | 0,000105151 | Cardiometabolic |
| Apolipoprotein F | Q13790 | -0,31 | 0,061949825 | 1,19526E-05 | 0,001613605 | Inflammation |
| Growth hormone receptor | P10912 | 0,14 | 0,028998154 | 2,97011E-05 | 0,004009651 | Inflammation |
| High affinity immunoglobulin alpha and immunoglobulin mu Fc receptor | Q8WWV6 | 0,07 | 0,016135343 | 4,79332E-05 | 0,006470979 | Cardiometabolic |
| Insulin-like growth factor-binding protein 2 | P18065 | -0,07 | 0,015954507 | 8,74034E-05 | 0,011799463 | Cardiometabolic |
| Creatine kinase B-type | P12277 | -0,07 | 0,015566715 | 9,99464E-05 | 0,013492762 | Cardiometabolic |
| Somatotropin | P01241 | -0,02 | 0,004534068 | 0,000126662 | 0,01709935 | Cardiometabolic |
| BPI fold-containing family B member 1 | Q8TDL5 | -0,07 | 0,01731678 | 0,000271315 | 0,036627512 | Cardiometabolic |
| Ectonucleotide pyrophosphatase/phosphodiesterase family member 7 | Q6UWV6 | 0,06 | 0,016291831 | 0,000293549 | 0,03962906 | Inflammation |
| Insulin-like growth factor-binding protein 1 | P08833 | -0,04 | 0,009657006 | 0,000351033 | 0,047389496 | Cardiometabolic |

**Caption:** significant association results displayed from the use of Linear regression model: ∆ Change_BMI ~ sex of the pair + Age at blood sample + ∆ Protein levels + mean Baseline_BMI. **Abbreviations:** SE: Standard error.

**Supplementary table 11.** Within-pair analysis assessing which of the previously identified associations between proteins and BMI fluctuation during adulthood remained significant when controlling for genetic confounding.

| **Protein Description** | **Protein ID** | **Estimate** | **SE** | **p value** | | **Protein panel** |
| --- | --- | --- | --- | --- | --- | --- |
|  |  |  |  | **Nominal** | **Bonferroni** |  |
| Na(+)/H(+) exchange regulatory cofactor NHE-RF3 | Q5T2W1 | 1,128874475 | 0,325847505 | 0,00116087 | 0,018573917 | Inflammation |
| E-selectin | P16581 | 1,972037277 | 0,631986687 | 0,003116475 | 0,049863598 | Cardiometabolic |

**Caption:** significant association results displayed from the use of Linear regression model: ∆ BMI_fluctuation ~ sex of the pair + Age at blood sample + ∆ Protein levels. **Abbreviations:** SE: Standard error.

**Supplementary table 12.** Information on diet and physical activity in the included sample by sex and in total

| Diet | | | |
| --- | --- | --- | --- |
| Portion size | Men | Women | All |
| Small | 10 (9%) | 52 (28%) | 62 (20%) |
| Moderate | 72 (61%) | 121 (65%) | 193 (63%) |
| Fairly Large | 28 (24%) | 11 (6%) | 39 (13%) |
| Large | 7 (7%) | 3 (1%) | 10 (4%) |
| Total | 117 (100%) | 187 (100%) | 304 (100%) |
| Physical activity | | | |
| Never | 7 (6%) | 9 (5%) | 16 (5%) |
| Rarely | 13 (11%) | 16 (9%) | 29 (9%) |
| Sometimes | 35 (30%) | 28 (16%) | 63 (21%) |
| Often | 48 (41%) | 95 (51%) | 143 (47%) |
| Regularly | 15 (12%) | 39 (19%) | 54 (18%) |
| Total | 117 (100%) | 187 (100%) | 304 (100%) |

**Caption****:** Sex specific and total number and-percentage of individuals in the different categories of the diet and physical activity confounders.

Interview items used to assess diet:

What is the typical size of your meal portion? (In the image, the portion has been taken from a ready meal tray onto a plate to be eaten)

1 – small (e.g. 1/3 of a 400g ready meal tray)

2 – moderate (e.g. 2/3 of a 400g ready meal tray)

3 – fairly large (e.g. 1 full 400g ready meal tray)

4 – large (e.g. 1 and 1/3 of a 400g ready meal tray)


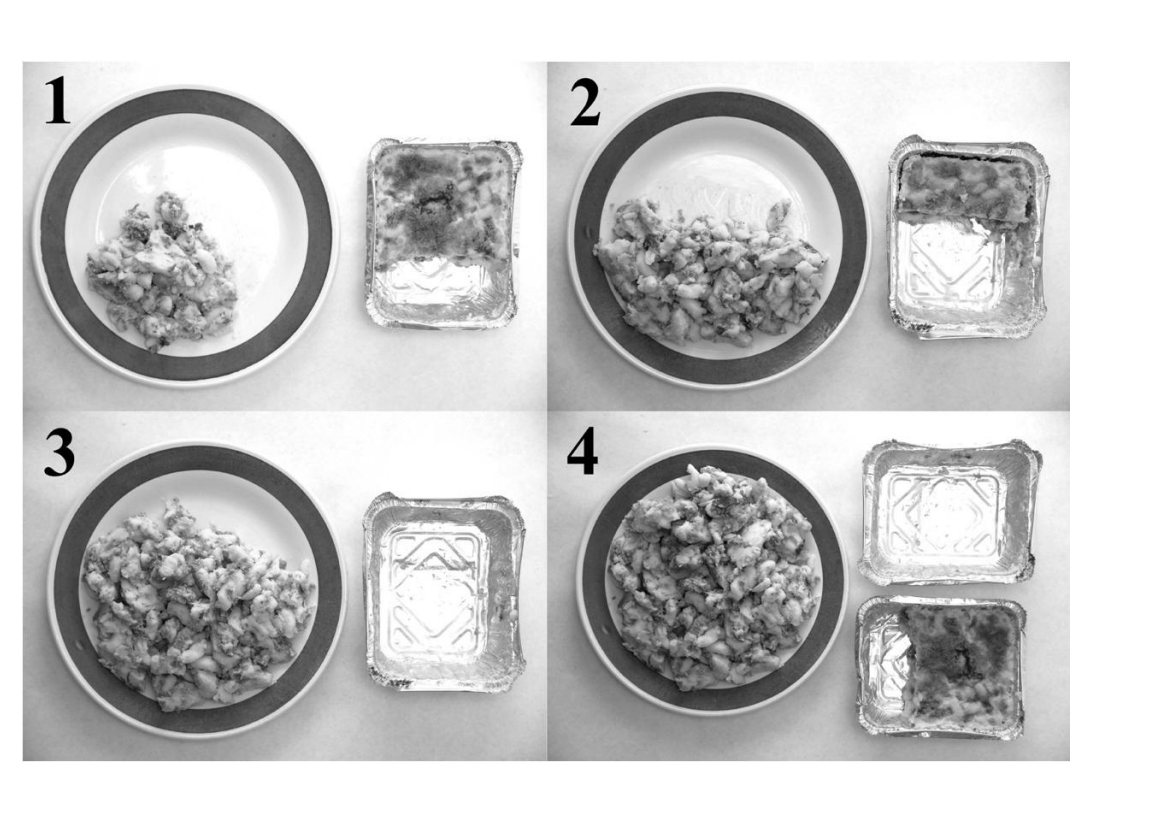


In my free time I do sports/exercise:

1 – never

2 – rarely

3 – sometimes

4 – often

5 – very often

**Supplementary table 13.** Systolic and Diastolic blood pressure mean and SD in the sample of the study and FINRISK 2012 by sex.

|  | Men | | | | Women | | | |
| --- | --- | --- | --- | --- | --- | --- | --- | --- |
|  | Current study (N=118) | | FINRISK 2012  (N= 2,774) | | Current study (N=186) | | FINRISK 2012  (N= 3,046) | |
|  | Mean | SD | Mean | SD | Mean | SD | Mean | SD |
| Systolic Blood Pressure | 148,8 | 14,44 | 140,8 | 17,9 | 140,6 | 18,29 | 137,1 | 17,9 |
| Diastolic blood pressure | 85,88 | 9,13 | 86,1 | 10,1 | 82,34 | 9,44 | 82,1 | 9,3 |

**Caption:** Mean and standard deviation of Systolic and Diastolic blood pressure for men and women from individuals of the current study are compared with those obtained from FINRISK in 2012. **Abbreviations:** N: Number of individuals; SD: Standard deviation.
